# RWRtoolkit: multi-omic network analysis using random walks on multiplex networks in any species

**DOI:** 10.1093/gigascience/giaf028

**Published:** 2025-04-24

**Authors:** David Kainer, Matthew Lane, Kyle A Sullivan, J Izaak Miller, Mikaela Cashman, Mallory Morgan, Ashley Cliff, Jonathon Romero, Angelica Walker, D Dakota Blair, Hari Chhetri, Yongqin Wang, Mirko Pavicic, Anna Furches, Jaclyn Noshay, Meghan Drake, A J Ireland, Ali Missaoui, Yun Kang, John C Sedbrook, Paramvir Dehal, Shane Canon, Daniel Jacobson

**Affiliations:** Computational and Predictive Biology Group, Oak Ridge National Laboratory, 1 Bethel Valley Rd, Oak Ridge, TN 37830, USA; Centre of Excellence for Plant Success in Nature and Agriculture, University of Queensland, St Lucia Campus Mansfield Place St Lucia QLD 4072, Australia; Computational and Predictive Biology Group, Oak Ridge National Laboratory, 1 Bethel Valley Rd, Oak Ridge, TN 37830, USA; The Bredesen Center for Interdisciplinary Research and Graduate Education, University of Tennessee Knoxville, 310 Ferris Hall 1508 Middle Dr Knoxville, TN 37996, USA; Computational and Predictive Biology Group, Oak Ridge National Laboratory, 1 Bethel Valley Rd, Oak Ridge, TN 37830, USA; Computational and Predictive Biology Group, Oak Ridge National Laboratory, 1 Bethel Valley Rd, Oak Ridge, TN 37830, USA; Computational and Predictive Biology Group, Oak Ridge National Laboratory, 1 Bethel Valley Rd, Oak Ridge, TN 37830, USA; Environmental Genomics and Systems Biology Division, Lawrence Berkeley National Laboratory Berkeley, Lawrence Berkeley National Laboratory 1 Cyclotron Road Berkeley, CA 94720, USA; Computational and Predictive Biology Group, Oak Ridge National Laboratory, 1 Bethel Valley Rd, Oak Ridge, TN 37830, USA; Computational and Predictive Biology Group, Oak Ridge National Laboratory, 1 Bethel Valley Rd, Oak Ridge, TN 37830, USA; The Bredesen Center for Interdisciplinary Research and Graduate Education, University of Tennessee Knoxville, 310 Ferris Hall 1508 Middle Dr Knoxville, TN 37996, USA; Computational and Predictive Biology Group, Oak Ridge National Laboratory, 1 Bethel Valley Rd, Oak Ridge, TN 37830, USA; The Bredesen Center for Interdisciplinary Research and Graduate Education, University of Tennessee Knoxville, 310 Ferris Hall 1508 Middle Dr Knoxville, TN 37996, USA; Computational and Predictive Biology Group, Oak Ridge National Laboratory, 1 Bethel Valley Rd, Oak Ridge, TN 37830, USA; The Bredesen Center for Interdisciplinary Research and Graduate Education, University of Tennessee Knoxville, 310 Ferris Hall 1508 Middle Dr Knoxville, TN 37996, USA; Computational Science Initiative, Brookhaven National Laboratory, PO Box 5000 Upton, NY 11973, USA; Computational and Predictive Biology Group, Oak Ridge National Laboratory, 1 Bethel Valley Rd, Oak Ridge, TN 37830, USA; Computational Science Initiative, Brookhaven National Laboratory, PO Box 5000 Upton, NY 11973, USA; Computational and Predictive Biology Group, Oak Ridge National Laboratory, 1 Bethel Valley Rd, Oak Ridge, TN 37830, USA; Computational and Predictive Biology Group, Oak Ridge National Laboratory, 1 Bethel Valley Rd, Oak Ridge, TN 37830, USA; The Bredesen Center for Interdisciplinary Research and Graduate Education, University of Tennessee Knoxville, 310 Ferris Hall 1508 Middle Dr Knoxville, TN 37996, USA; Computational and Predictive Biology Group, Oak Ridge National Laboratory, 1 Bethel Valley Rd, Oak Ridge, TN 37830, USA; Computational and Predictive Biology Group, Oak Ridge National Laboratory, 1 Bethel Valley Rd, Oak Ridge, TN 37830, USA; Department of Crop and Soil Sciences, University of Georgia, Miller Plant Sciences Building 120 Carlton Street Athens GA 30602, USA; Computational Science Initiative, Brookhaven National Laboratory, PO Box 5000 Upton, NY 11973, USA; Noble Research Institute, 2510 Sam Noble Parkway Ardmore, OK 73401, USA; Driscoll’s Inc., 345 Westridge Dr, Watsonville, CA 95076, USA; School of Biological Sciences, Illinois State University, School of Biological Sciences Illinois State University Campus Box 4120 Normal, IL 61790, USA; Department of Crop and Soil Sciences, University of Georgia, Miller Plant Sciences Building 120 Carlton Street Athens GA 30602, USA; Department of Crop and Soil Sciences, University of Georgia, Miller Plant Sciences Building 120 Carlton Street Athens GA 30602, USA; Computational and Predictive Biology Group, Oak Ridge National Laboratory, 1 Bethel Valley Rd, Oak Ridge, TN 37830, USA

**Keywords:** multiplex network, systems biology, random walk with restart, multi-omic, software package

## Abstract

We introduce RWRtoolkit, a multiplex generation, exploration, and statistical package built for R and command-line users. RWRtoolkit enables the efficient exploration of large and highly complex biological networks generated from custom experimental data and/or from publicly available datasets, and is species agnostic. A range of functions can be used to find topological distances between biological entities, determine relationships within sets of interest, search for topological context around sets of interest, and statistically evaluate the strength of relationships within and between sets. The command-line interface is designed for parallelization on high-performance cluster systems, which enables high-throughput analysis such as permutation testing. Several tools in the package have also been made available for use in reproducible workflows via the KBase web application.

## Background

Biological studies are increasingly pursuing and obtaining data on larger scales and at multiple levels in the molecular hierarchy of the study system. One approach to dealing with the multiplicity of data in modern biology is to represent the relationships in the data as a network [1]. Each entity in a dataset (e.g., each gene) becomes a node, and an edge between two nodes represents a relationship that has been measured or predicted between those nodes (e.g., their coexpression in a population, sharing of common protein domains, or similarity of methylation state). Once in network form, a great variety of network analysis methods becomes available [2,3]. Genes that are strongly connected to each other are topologically more likely to be functionally relevant to each other than more distal or loosely connected genes in the network. Machine-learning algorithms can be used to efficiently explore entire networks and find such relationships with respect to a set of starting genes, often called seeds or anchors. This approach is particularly useful for exploring the functional context around sets of genes, such as those produced from genome-wide association studies (GWAS), quantitative trait loci (QTL) mapping, differential expression analysis, or case/control proteomics.

To analyze a set of genes in a network context, a network and an algorithm to traverse that network are required. The underlying network may be as simple as a single-layer of nodes and edges generated from one experimental dataset that predicts relationships between genes, such as a co/predictive expression relationship determined from RNAseq results [4,5].

There exist many types of relationships in biological systems because many types of measurements exist, such as proteomic assays, bulk and single-cell RNAseq transcription, metabolomic profiling, and more. These different omics “layers” offer distinct insights into the mechanisms within that layer of questioning. Relationships within biological systems, however, are not isolated within the layer in which their data were measured. The internal mechanisms of the cell interact not only with similar layer elements, but also these mechanisms are heavily involved in interlayer interaction as well [6].

Combining these differing omics layers for downstream analysis can be a difficult process. Commonly, the multiple layers of input networks are aggregated into a single layer by summing or averaging multiple edges between the same pair of nodes into one composite edge. The result is a single adjacency matrix representation of the data, also known as a monoplex network. The aggregated network is a summarization of the input layers, and as such has lost the unique topological information carried by each layer.

A more robust approach to combining these heterogeneous data layers is to incorporate these differing lines of evidence into a multigraph, which are networks that can contain multiple edges between two vertices. [7]. A multigraph network potentially fills in relationship gaps that exist in any given single layer and enables simultaneous exploration of multi-omic data because one can traverse the network from node to node using edges from all layers. When relationships between specific nodes are present in multiple layers, a multilayer network presents multiple lines-of-evidence (LOE) that those genes are functionally related [8].

**Figure 1:**
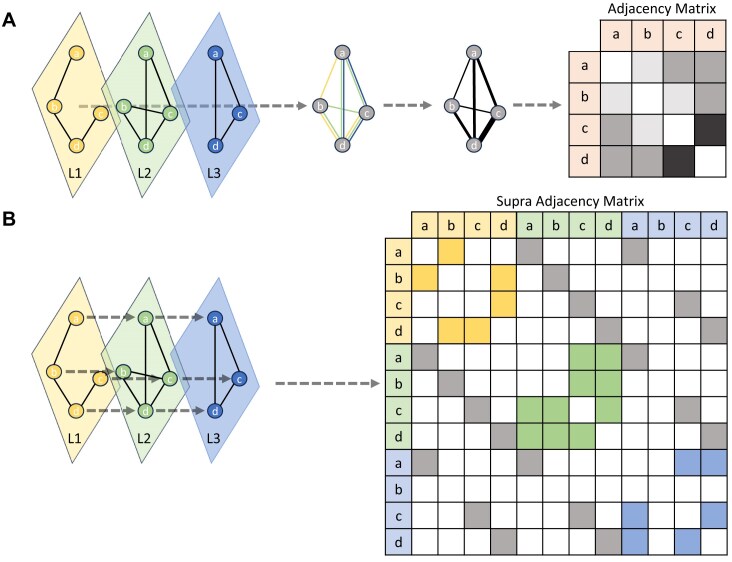
Aggregated monoplex network vs multiplex network. In this example there are three small input network layers (L1, L2, L3), with a union set of nodes of size *n* = 4, with which to generate a multilayer network. (a) In the aggregated approach the layers are merged into one. If multiple edges occur between any pair of nodes, their weights are aggregated to produce the final adjacency matrix of size *n* × *n*. (b) In the multiplex approach each layer is kept separate via a supra-adjacency matrix of size (*n* × *L*) × (*n* × *L*), where *L* is the number of layers. Nodes that are common across layers are connected by virtual edges (gray arrows). The diagonal blocks of the supra-adjacency matrix represent the standard adjacency matrices within each individual layer. Connectivity between layers is represented in the off-diagonal blocks, with virtual edges shown in gray. Note that layer L3 (blue) does not contain node ‘b’, so there are no interlayer virtual edges from L1–L3 or L2–L3 for node ‘b’.

Multiplex networks are types of multigraphs that are structured such that, for all possible types of connections, there exists a corresponding layer for those types of edges to exist and connections between all nodes of the same kind between layers [9]. This multiplex structure is achieved through the incorporation of individual layers’ adjacency matrices along the diagonal of the “supra-adjacency” matrix, with interlayer edges connecting corresponding nodes across layers (Fig. 1). Multiplex network representations of multi-omic systems have been demonstrated to outperform other methods of network aggregation [10–13]. Although the predictive capability of the multiplex networks does exceed that of monoplex networks, the supra-adjacency matrix is more difficult to build and adds an extra level of complexity to network exploration algorithms.

A wide variety of algorithms exist for ranking genes according to their topological connectivity to the seed (candidate) genes in the underlying network. One simple approach, known as neighbor voting [14], scores each gene by counting their outgoing edges that directly connect to the seeds. However, by only looking at immediate connections to the seeds, the influence and importance of genes farther away is ignored. Other traditional algorithms for exploring biological networks include calculating clustering coefficients, centrality metrics, and network density [15]. More advanced propagation approaches, such as diffusion and random walk with restart (RWR), use the entire network topology to score and rank every node, and have been shown to be generally superior in their ability to find true positive relationships [16–18]. A random walk can be described conceptually as a “walker” which proceeds to wander outwards from a starting seed gene, choosing which edge to take with a probability equal to 1/*d*, where *d* is the degree of the current gene (*d* is the out-degree for directed networks). Over multiple iterations, the walker explores the network in this manner so the proportion of time spent at each gene forms a probability distribution that represents how accessible every gene in the network is when starting from one or more seed gene(s). With RWR, at each iteration the walker teleports back to the starting point with restart probability *r* to prevent the walker from wandering too far in the global topology, or getting stuck in various topological structures.

The R package RandomWalkRestartMH provides the essential functionality for implementing RWR on multiplex networks, but focuses primarily on executing the algorithm itself [13]. Therefore it lacks many of the higher-level features often needed in sophisticated biological analyses, leaving researchers to piece together additional tools for tasks such as multiplex network construction, network validation, parallelized permutation testing, and neighborhood analysis. The use of the RandomWalkRestartMH package additionally assumes a passing knowledge of the R environment for users.

Here we introduce RWRtoolkit [19], an R package that builds on RandomWalkRestartMH and fills these gaps through functionalities with a corresponding set of command-line tools designed for ease of use, enabling the easy construction of multiplex networks from any set of data layers, followed by analysis of candidate gene sets within the networks using the RWR algorithm. We have additionally integrated RWRtoolkit with a graphical user interface (GUI) into the Department of Energy’s (DOE) Knowledge Base (KBase) system [20].

RWRtoolkit extends and updates the RandomWalkRestartMH R package [13], which provides the core functionality to generate multiplex networks from a set of input network layers, and implements the RWR algorithm on a supra-adjacency matrix. Once a multiplex network has been generated, the RWRtoolkit provides commands to rank all genes in the overall network according to their connectivity to a set of seed genes, use cross-validation to assess the network’s predictive ability or determine the topological similarity of a set of genes, and find shortest paths between sets of seed genes. The RWRtoolkit R package outputs detailed tables of ranked genes as well as statistics of predictive accuracy (AUROC, AUPRC, etc.), plots, and network visualizations of the multi-omic neighborhood around the seed genes. Furthermore, RWRtoolkit commands can be run from the command-line interface, which enables high-throughput parallelized analysis (such as permutation testing) on compute clusters.

To date, multi-omic networks have been made publicly available in a range of model species: AraNet, PopGenie, StringDB, YeastNet. However these networks are often aggregated into a single layer rather than multiplexed, and it is difficult or impossible for the user to customize their choice of input layers or include custom layers generated from their own experimental results or algorithms. RWRtoolkit enables network analysis of multi-omic data for any species, allowing researchers to use their own datasets and networks and/or pre-existing networks. We demonstrate this by generating a custom *Arabidopsis thaliana* multiplex network and using it to analyze gene sets from a novel GWAS study and a published gene knockout study [21] (see Supplemental Case Studies). RWRtoolkit is available with installation instructions, user guide, and sample data at http://github.com/dkainer/RWRtoolkit [22].

## Data description

The RWRtoolkit codebase provides functions for multiplex network generation, running random walks starting from given seed sets, validation functions for seed sets as well as network layers, and general multiplex network statistics. These functions are made available via a cross-platform R package with command-line interface commands and a web GUI. We have provided multiple tutorials in the R vignette format for users to explore at their own leisure. Our methods were generated with gene relationships as a focus, but these random walk methods can be applicable to any data type in network format. Example methods can be seen in Table 1 and example input and output can be seen in Supplementary Tables S1–S18.

**Table 1:**
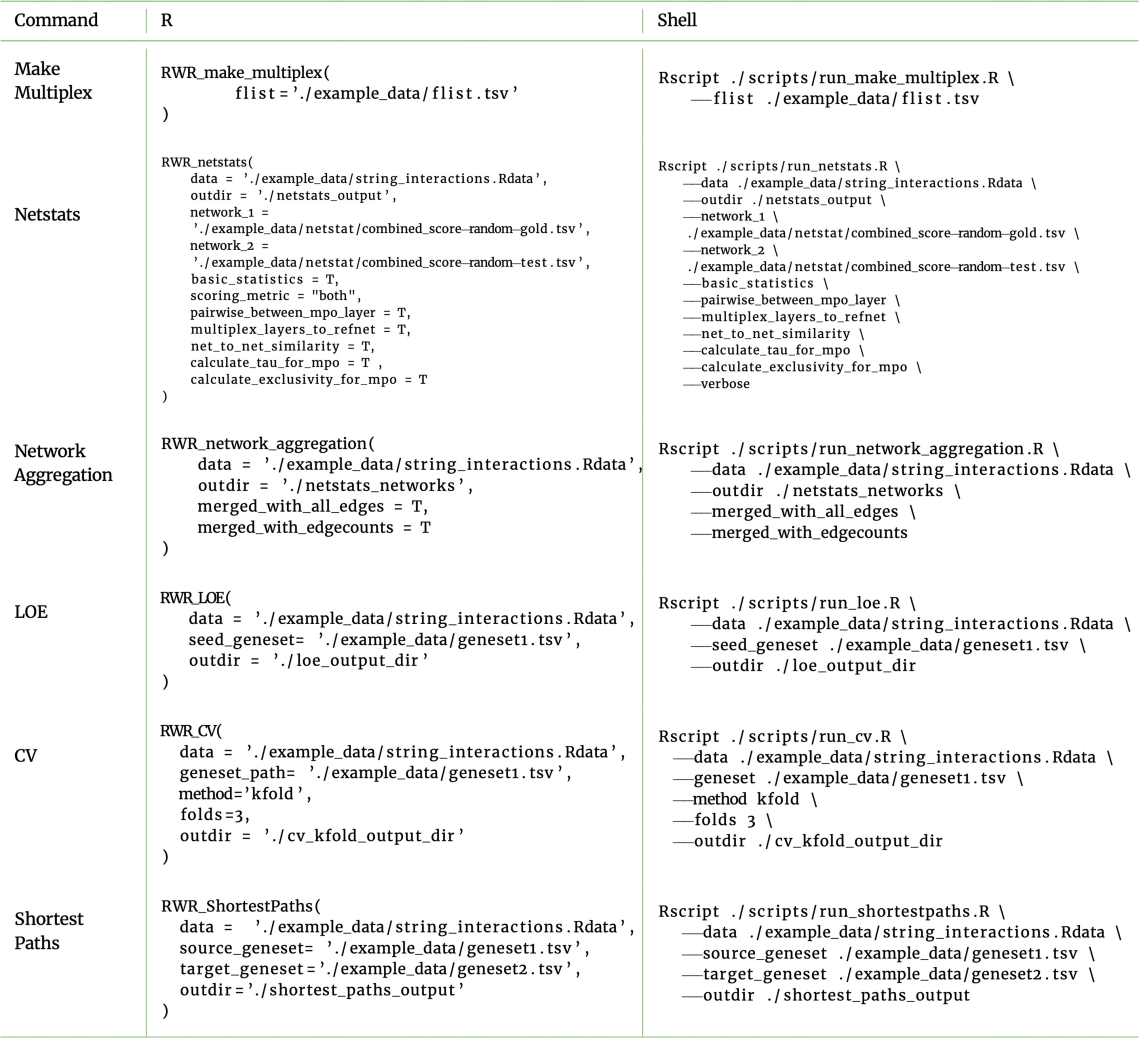
RWRtoolkit function calls from either an R environment or the command line.

**Figure 2:**
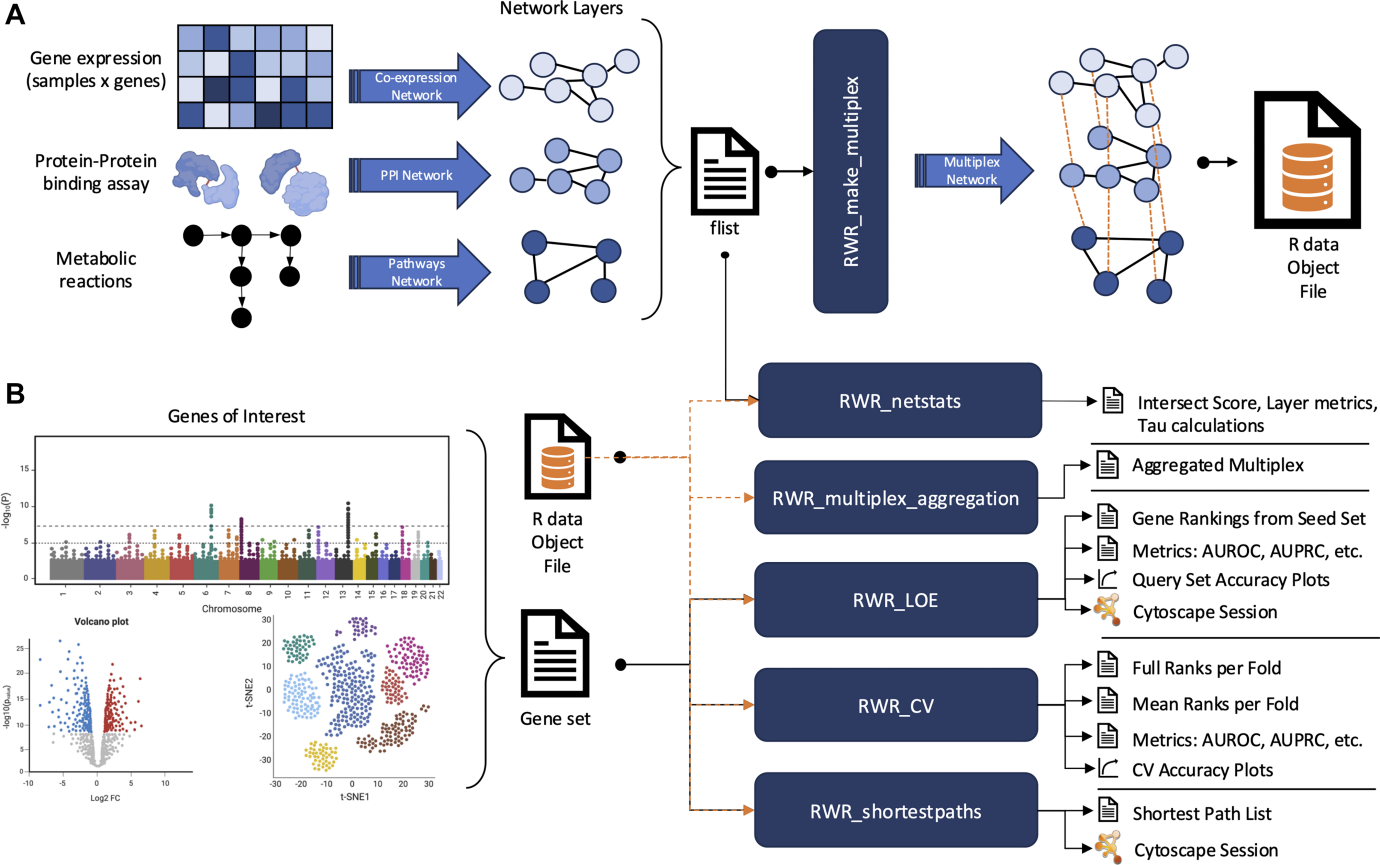
A general workflow for using the RWRtoolkit. (a) Illustration of how a user can generate several network layers from different omics data sources, which become input to the RWRtoolkit workflow. Once the user has networks in the correct format, they can then refer to them via a flist file and use RWR_make_multiplex to turn them into a homogeneous multiplex network (e.g., multiple layers of gene-to-gene relationships). This multiplex is wrapped in an Rdata object that is saved for future use. (b) A demonstration of how the user can now execute a variety of multi-omic analyses, most of which require the Rdata object as input. A set of genes of interest (gene set) from discovery studies such as GWAS or differential expression analysis can be used as input to multiple tools. These tools output a variety of files that show how functionally connected the genes in the gene set are to each other, or to a second gene set of interest, or to all the other genes in the multiplex. Some resulting networks can be automatically visualized in Cytoscape via the RCy3 R package [23]. This figure uses illustrations created with BioRender.com.

We created the RWRtoolkit R package as a collection of functions designed with ease of use in mind, particularly for users who are not familiar with the R environment as all input values for the functions take file paths as input (Fig. 2). We have additionally implemented updates to the RandomWalkRestartMH package.

### RandomWalkRestartMH Updates

For broad installation purposes, we have removed the DNET package from our implementation of the package because it is no longer supported by CRAN. This ensures ongoing compatibility with CRAN and helps maintain the stability of our software. In our updated RandomWalkRestartMH codebase, the code relies on parallel computing constructs rather than a straightforward sequential approach. Instead of iterating through multiplex network layers in nested loops, multiple workers are spawned, and updates to the layers within the supra-adjacency matrix are implemented in parallel. This shifts the logic flow by segmenting operations across concurrent processes, which can speed up large computations. The added parallel step does introduce new system demands, notably requiring additional libraries and configuration for managing distributed tasks. Despite this overhead, the updated code can handle large multiplex networks more effectively. In addition, the previous iteration of the RandomWalkRestartMH package suggested that the parameter Tau exerted little influence on the final output. We found that the original codebase implementations for calculating Tau across the layers contained an error and have implemented a fix. Our findings indicate that adjusting Tau can indeed affect the multiplex network’s overall performance, resulting in notable changes to output scores and rank ordering.

### Multiplex generation

RWRtoolkit workflows typically start with the RWR_make_multiplex command, which handles the creation of the multiplex network. It requires a descriptor file (known as an “flist”) that lists the full path to each network layer to be included in the multiplex. Each network layer’s file must be formatted as a simple delimited edge list with a column for the source genes, a column for the target genes, and an optional weight column (see Supplementary Tables S1–S3 for file examples). This function combines the creation of the multiplex object, the supra-adjacency matrix, as well as the normalized supra-adjacency matrix. The generation of the multiplex object and supra-adjacency matrix use updated RandomWalkRestartMH functionality that parallelizes the multiplex object creation and supra-adjacency matrix generation. This updated methodology employs a task-mapping strategy to directly assign values of 1 to the corresponding $i,j$ edges across all network layers in the supra-adjacency matrix, in contrast to the previous approach, which embedded complete adjacency matrices along the diagonal of the supra-adjacency matrix.

The generated multiplex network is automatically saved as an Rdata object containing the individual layers as igraph [24,25] networks, the multiplex supra-adjacency matrix, transition matrix, and network metadata. This Rdata object is used as input for most downstream commands.

### Multiplex RWR applications

#### Network layer and multiplex statistics

The contents of a multiplex network affect the outcomes of RWR analyses. The RWR_netstats command lets the user evaluate individual network layers or the contents of an entire multiplex network containing many layers. Basic statistics (basic_statistics) for individual layers can be calculated, as well as more complex relationships such as jaccard or overlap scores for interlayer similarities (pairwise_between_mpo_layer), multiplex layer to reference network (multiplex_layers_to_refnet), and single network to network similarities (net_to_net_similarity). Additionally, the RWR tau parameter affects the probability of the walker visiting each specific layer, allowing the user to bias the walk to certain layers of higher importance. Users can supply their own tau values, or use the calculate_tau function to return a tau value for each layer based on each layer’s overlap with a gold-standard network.

#### Evaluating multiplex networks and gene sets using cross-validation

The predictive ability of a multiplex network can be determined using cross-validation of gold standard or reference gene sets with the RWR_CV command. A gold standard gene set typically contains genes that are known to be functionally related (all are members of one biosynthetic pathway, all are annotated with the same GO/KEGG term, etc.). The hypothesis is that gold standard genes purposely left out from the seed set should be found with relatively high precision (i.e., highly ranked by RWR) if the underlying networks are indeed functionally predictive. The RWR_CV command allows the user to provide a gold standard gene set and use *k*-fold, leave-one-out, or singleton cross-validation to score the ability to find the left-out gene(s).

RWR_CV generates output files that include the RWR score and rank of each gene in the multiplex (as detailed by RWR_LOE for each fold), the mean rank of each gene across all folds, evaluation metrics based on the ranks of seed genes for each fold, and a comprehensive evaluation summary file. Users can select from three methods to test gene sets against networks: KFold, where each seed gene *N* is ranked *K* − 1 times across *K* folds, yielding *K* − 1 RWR scores per gene (Supplementary Figure S1A); Leave One Out (LOO), where each seed gene *N* is ranked across *N* − 1 folds, with one gene left out from the seed set per fold; and Singletons, where each seed gene *N* is individually ranked *N* −1 times across *N* folds, with only one single seed gene per fold. Metric calculations for determining precision and recall with respect to ranked data per fold are derived from Järvelin and Kekäläinen [26]. A more in-depth discussion of metric calculation can be found in the supplemental material. File descriptions and examples can be found in Supplementary Tables S14– S16.

#### Ranking genes using multiple LOE

The RWR_LOE command uses RWR to rank all genes in the multiplex network with respect to a gene-set of interest (seed genes), which provides a multi-omic biological context for the seeds. The ranks and scores of all genes can be output to a file. A second gene set can be provided to evaluate the topological relationship between two sets of genes. When a second gene set is provided, those genes are flagged within the ranked output. The network context around the top *N* ranked genes can be easily visualized via an integrated connection to Cytoscape [27] using the RCy3 [23] R package with the –cyto=N flag.

#### Extracting the shortest paths across the multiplex between genes

In a network, many unique paths between two particular nodes can exist. Obtaining the shortest paths between any two given nodes within a network can provide crucial insight to a network’s topology or the relationship between those nodes. RWR_ShortestPaths calculates the pairwise shortest paths between source and target gene sets, and returns them as a series of edges that form the shortest path, the layers in which those edges exist, edge weights, and normalized edge weights. The shortest paths between seed and target sets can additionally be automatically visualized in Cytoscape using the –cyto flag.

#### Network aggregation functions

Two methods of network aggregation are provided to merge the layers of a multiplex network into a single monoplex network. The merged_with_all_layers function aggregates all layers maintaining multiple edges between nodes. The merged_with_all_edgecounts function aggregates all layers of the multiplex, but instead edge weight is calculated as the sum of all shared edges within the multiplex network.

## Results

### RandomWalkRestartMH multiplex generation updates

**Figure 3:**
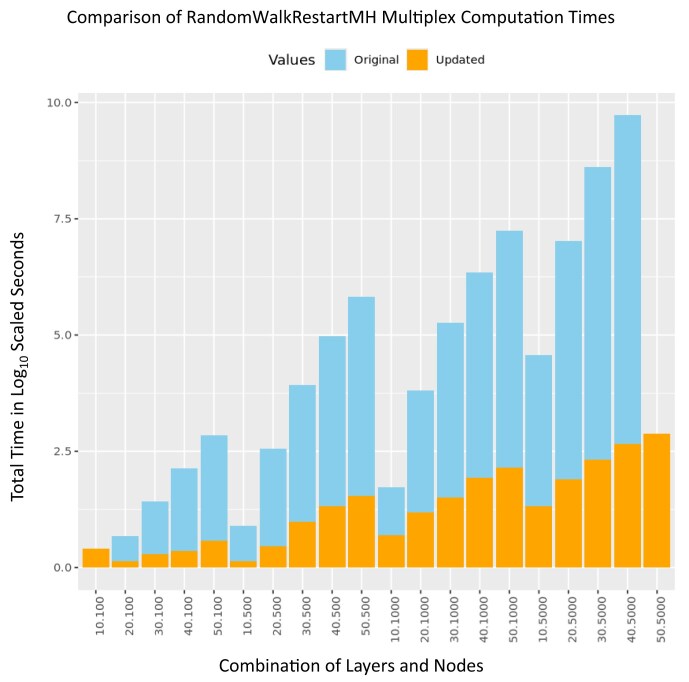
Multiplex generation timing: comparing the compute time for two separate implementations of the generation of a multiplex object, supra-adjacency matrix, and transition matrix in log scale. The generation of multiplex networks in the original implementation (blue) takes significantly longer to process compared with its updated counterpart (orange). The speedup is due to the parallel processing of edge updating within the supra-adjacency matrix across 32 cores.

Based on comparing the multiplex generation methods for creating the multiplex network object, generating the supra-adjacency matrix, and generating the transition matrix of the previous iteration of RandomWalkRestartMH and the current, parallelized version, our parallelized method for calculating multiplex networks at scale surpasses the previous implementation of multiplex generation. Figure 3 demonstrates that as node count as well as layer count increase, the amount of time required for multiplex generation increases accordingly. Our updated implementation significantly improves performance, particularly for larger networks with greater numbers of nodes and layers. It is particularly noteworthy that the network of 50 layers timed out after 6 hours of construction whereas the updated parallelized method constructed the network in 17.69 seconds.

### RandomWalkRestartMH tau updates

**Figure 4:**
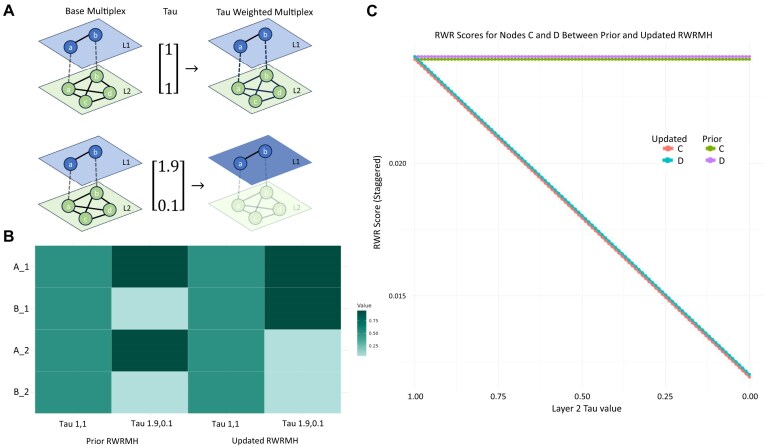
Tau comparison. (a) For a multiplex, setting each layer’s Tau to a different value should alter the weight of the seed nodes within those layers, causing RWR to be biased towards exploring those layers with higher Tau values. (b) Comparing the original RandomWalkRestartMH functionality for updating seed vectors with Tau to the updated version illustrates that both versions work properly with respect to equal weighting. When given a Tau to weigh layer 1 with 1.9 and layer 2 with 0.1, the original implementation weighs seed A with 1.9 and seed B with 0.1 across layers. Conversely, the updated implementation weighs the layers with Tau accordingly. (c) Given random walks with seeds A and B, the RWR scores of nodes C and D do not change as Tau for layer 2 is decreased to 0 with respect to the original implementation. In the updated implementation, as Tau for layer 2 decreases, the RWR scores of C and D decrease accordingly.

Testing with a two-layer multiplex illustrates that the original implementation of get.seed.scoresMultiplex produced incorrect seed weight adjustments, whereas the updated version reflects seed weights weighted by layer (i.e., higher scores for layer 1, lower scores for layer 2) (Fig. 4a,b). Similarly, the revised Random.Walk.Restart.Multiplex function behaves as expected when the tau parameter decreases, causing proportional reductions in non-seed nodes (C and D) in layer 2. In contrast the original implementation shows no changes in these scores as tau decreases (Fig. 4c).

### RWRtoolkit applications

#### Multiplex construction

**Figure 5:**
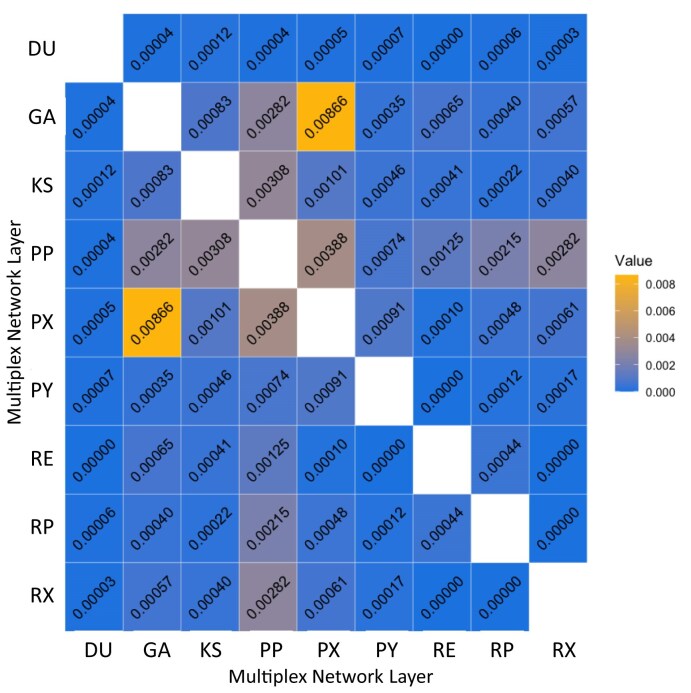
**Pairwise Between MPO Layer Jaccard Netstats:** Jaccard similarity coefficients are calculated for the edges of each network with respect to each other network layer in the multiplex. Overall, there is little overlap in edges between network layers, illustrating the heterogeneity of information encoded within each layer. The layers that contain the greatest amount of jaccard similarity are PP and PX, representing protein–protein interaction and predictive expression, respectively.

We constructed our comprehensive *Arabidopsis* multiplex with a total of nine layers ranging from 789 to 19,975 nodes. The full multiplex took 59 seconds to construct and contains 26,605 unique nodes and 918,640 edges (see Data availability section for multiplex links). We used the RWR_netstats function to calculate basic statistics and pairwise jaccard comparison between all layers in the multiplex, producing two output files: base_stats.tsv and pairwise_between_mpo_layer_jaccard.tsv, illustrated in Table 2 and Fig. 5, respectively.

**Table 2:** RWR Netstats basic statistics for the comprehensive *Arabidopsis thaliana* multiplex.

Network name	Number of nodes	Number of edges	Diameter
DU	2,283	13,514	13
GA	7,683	84,959	22
KS	1,841	94,952	6
PP	19,191	317,787	16
PX	19,975	145,407	12
PY	13,314	71,287	9
RE	789	1,359	16
RP	16,014	167,851	6
RX	2,857	21,524	19

#### Network validation

**Figure 6:**
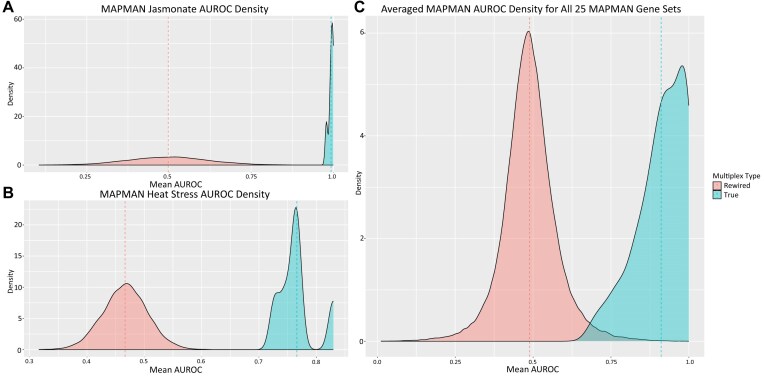
A comparison of the mean AUROC scores from the *k*-fold output of RWR_CV using the true comprehensive multiplex (blue) and 1m000 randomly rewired multiplex networks (red). (a) Illustration of a comparison of AUROC density across five folds using an individual set of genes curated for Jasmonate signaling obtained from MAPMAN. The true comprehensive multiplex (blue) has a mean AUROC across five folds of 0.993, whereas the 1,000 rewired multiplexes have an average mean AUROC across five folds of 0.498. (b) Depiction of a comparison of AUROC density across five folds using an individual set of genes curated for heat stress signaling obtained from MAPMAN. The true comprehensive multiplex has a mean AUROC across five folds of 0.766. The average mean AUROC across five folds for the 1,000 rewired multiplexes is 0.467. (c) Illustration of a comparison of the average AUROC density across 25 gold standard gene sets generated from shared MAPMAN terms including Jasmonate and heat stress signaling. The true comprehensive multiplex has an overall average mean AUROC of 0.91 across all 25 gold standard gene sets, whereas the 1,000 rewired multiplex networks have an overall average AUROC of 0.489 across all 25 gold standard gene sets, illustrating that the true comprehensive multiplex has meaningful biological connections compared to the completely random connections found across the 1,000 rewired multiplex networks.

We ran RWR_CV using *k*-fold (*k* = 5) on each of 25 MAPMAN-derived [28] gene sets to validate the predictive ability of the multiplex network for gene function. This resulted in an average AUROC of 0.91 across all gene sets and CV folds, indicating a strong overall ability to find the left-out genes from a functional group and rank them highly. When we performed the same analysis on 1,000 randomly rewired multiplexes using command-line RWR_CV, the overall average AUROC was 0.49 (where 0.50 is considered the equivalent of random). A comparison of AUROC densities for individual MAPMAN gene sets is illustrated in Fig. 6a,b and the aggregate of all MAPMAN gene set AUROC densities is illustrated in Fig. 6c. Individual RWR_CV comparison statistics for each MAPMAN gene set can be found in Table 3.

**Table 3:** Mean and standard deviation of *k*-fold AUROC scores for comprehensive and rewired multiplexes.

	Rewired 1,000	Rewired 1,000	Comprehensive net	Comprehensive net
Gene set	Mean AUROC	SD AUROC	Mean AUROC	SD AUROC
Abscisic acid	4.99E-01	7.73E-02	8.71E-01	6.95E-02
Auxin	4.81E-01	4.30E-02	8.42E-01	3.98E-02
Brassinosteroids	4.89E-01	9.98E-02	9.39E-01	4.64E-02
Cell wall synthesis	5.01E-01	5.55E-02	9.82E-01	1.27E-02
CHO metabolism	4.97E-01	4.08E-02	9.39E-01	3.07E-02
Coldstress	5.01E-01	1.75E-01	9.81E-01	1.87E-02
Cytokinin	4.95E-01	1.27E-01	9.06E-01	7.55E-02
Drought salt	4.98E-01	8.28E-02	8.69E-01	3.91E-02
Ethylene	4.80E-01	6.08E-02	8.77E-01	4.81E-02
Ethylene and EREBP	4.93E-01	4.05E-02	9.25E-01	7.18E-03
Fatty acid	4.91E-01	5.46E-02	9.32E-01	4.25E-02
Flavonoids	5.02E-01	7.09E-02	9.48E-01	2.88E-02
Gibberelin	4.78E-01	9.54E-02	8.50E-01	9.14E-02
Glucosinolates	4.99E-01	7.31E-02	9.39E-01	3.61E-02
Heat stress	4.67E-01	3.90E-02	7.66E-01	3.48E-02
HeatshockTFs	4.66E-01	3.86E-02	7.69E-01	5.85E-02
Isoprenoids	4.99E-01	5.62E-02	9.85E-01	1.51E-02
Jasmonate	4.98E-01	1.21E-01	9.93E-01	7.94E-03
Lignin biosynthesis	5.03E-01	1.10E-01	9.99E-01	3.11E-04
Major CHO	4.90E-01	5.87E-02	9.14E-01	4.16E-02
Phenylpropanoids	5.01E-01	7.70E-02	9.97E-01	2.33E-03
PS light reaction	4.53E-01	4.63E-02	8.41E-01	3.27E-02
PS light reaction II	4.58E-01	7.55E-02	9.02E-01	1.36E-02
Salicylic acid	5.01E-01	1.50E-01	8.99E-01	1.08E-01

This table contains the mean values and standard deviation (SD) of AUROC for RWR_CV Kfold cross-validation for 25 curated gene sets which all share the same MAPMAN term. The mean and SD values for the rewired networks are averages and SD across 1,000 iterations of the RWR_CV Kfold cross-validation in which the edges of each network were rewired for each iteration. Conversely, the mean and SD values for the comprehensive network alone are those pertaining only to the average values across the single RWR_CV Kfold cross-validation (*k* = 5).

#### Running a random walk on a multiplex

**Figure 7:**
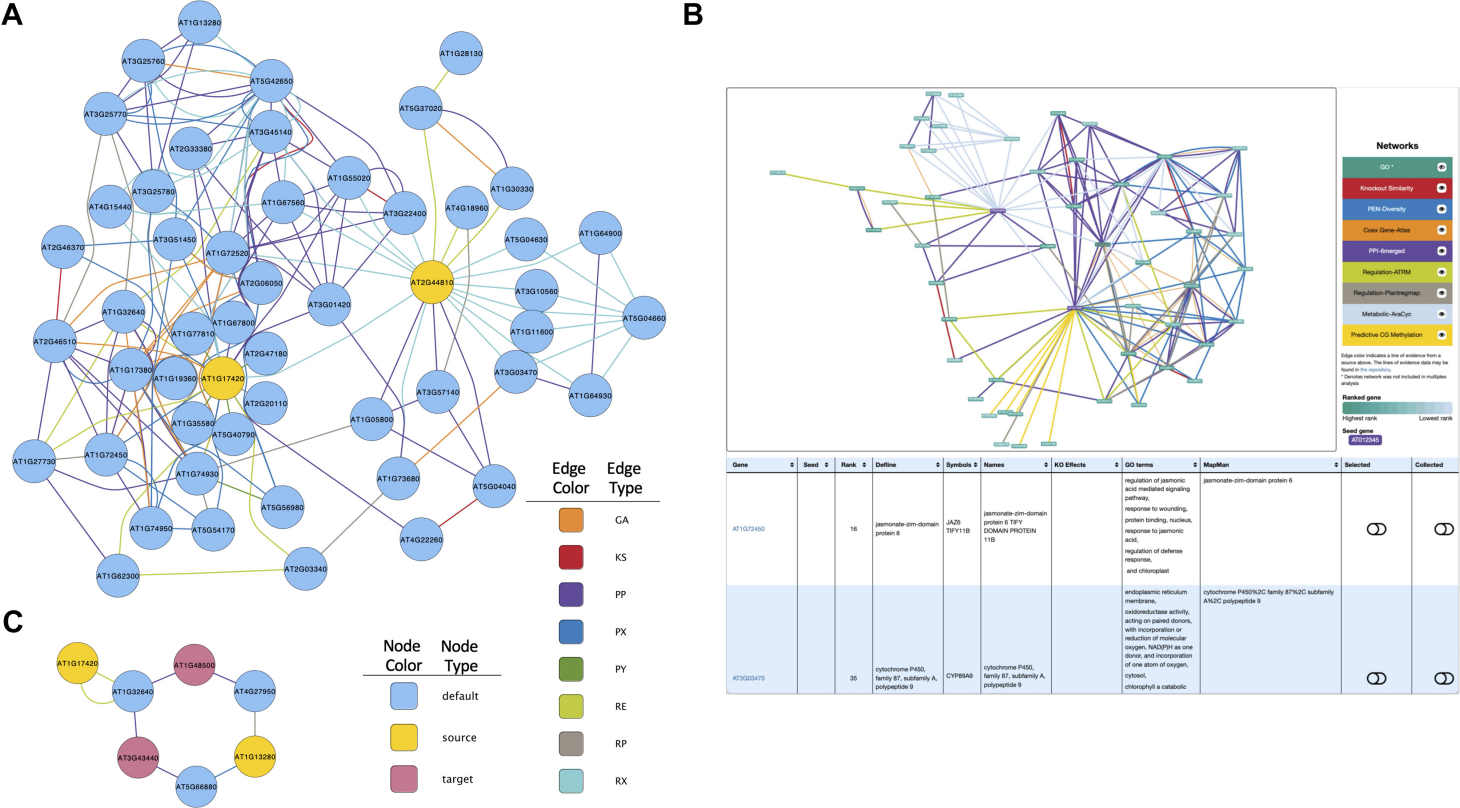
RWRtoolkit multiplex visualizations. (a) Using the RWR_LOE function with the –cyto flag, users can specify the total number of nodes they wish to see in the Cytoscape visualization. The total multiplex is aggregated for visualization purposes with all edge types maintained for visual styling. (b) The RWR_LOE function within the KBase application automatically visualizes the network output, illustrating the layers from which each edge came on the same screen as the RWR_LOE node score output. (c) RWR_ShortestPaths finds the shortest paths between any two sets of nodes and plots the subnetwork into Cytoscape.

Using the RWRtoolkit software application to run RWR_LOE, we used AT2G44810 and AT1G17420 as seeds with the cyto parameter set to 50 to visualize the subnetwork of the top 50 ranked genes and all lines of evidence connecting those genes (Fig. 7a). The full score and rankings for all genes in the multiplex were reported to file. The top-50 subnetwork was aggregated and sent to Cytoscape via the RCy3 API with all edges colored within the visualization by layer name. Conversely, the web-based KBase implementation visualized the top 50 selected genes within a separate window with interactive javascript functionality (Fig. 7b).

#### Evaluating the connectivity of a set of genes

Using 5-fold RWR_CV we ranked 30 seed genes (27 MAPMAN Jasmonate genes and 3 from differing ontology terms). The average rank per gene is denoted in Table 4. Almost all (23/27) of the jasmonate genes were ranked in the top 100, while the remaining 4 jasmonate genes, AT5G20900 AT3G17860 AT3G43440 and AT1G48500, had relatively high ranks of 128, 246, 1,517, and 1,947, respectively. The 3 non-jasmonate genes, AT4G33360, AT5G23010, and AT1G08380, were ranked considerably lower at 2,600, 9,426, and 9,707, respectively. The KBase application reports the same files and information.

**Table 4:** RWR CV LOO cross-validation to explore how well connected each node is with respect to all remaining nodes within the seed set of interest.

NodeNames	meanrank	rerank
AT1G13280	1	1
AT1G17420	1	1
AT1G72520	1	1
AT3G25760	1	1
AT5G42650	1	1
AT2G06050	2	6
AT3G22400	2	6
AT3G25770	2	6
AT3G25780	2	6
AT3G45140	2	6
AT1G17990	3	11
AT1G18020	3	11
AT1G55020	3	11
AT1G67560	5	14
AT1G17380	7	16
AT1G72450	8	17
AT2G44810	9	18
AT1G74950	15	23
AT1G09400	23	28
AT1G76690	26	29
AT1G76680	27	30
AT1G19180	29	31
AT1G70700	45	44
AT5G20900	277	128
AT3G17860	519	246
AT3G43440	2063	1517
AT1G48500	2607	1947
AT4G33360	3280	2600
AT5G23010	9382	9425
AT1G08380	9644	9707

#### Extracting the shortest paths

Extracting the shortest paths from the source genes AT1G13280 and AT1G17420 and target genes AT1G48500 and AT3G43440 revealed that all 4 paths had a length of 3; these are reported in Table 5. The cyto flag exports a visualization of all nodes within the shortest paths in Cytoscape (Fig. 7c).

**Table 5:** RWR shortest paths from the source AT1G13280 and AT1G17420 to the targets AT1G48500 and AT3G43440.

				Weight	Path	Path	Path
From	To	Weight	Type	norm	name	length	elements
AT1G48500	AT4G27950	1	PP	3.15E-06	AT1G13280_AT1G48500	3	AT1G13280$\rightarrow$AT4G27950$\rightarrow$AT1G48500
AT4G27950	AT1G13280	1	RP	5.96E-06	AT1G13280_AT1G48500	3	AT1G13280$\rightarrow$AT4G27950$\rightarrow$AT1G48500
AT5G66880	AT3G43440	1	PP	3.15E-06	AT1G13280_AT3G43440	3	AT1G13280$\rightarrow$AT5G66880$\rightarrow$AT3G43440
AT5G66880	AT1G13280	1	PX	6.88E-06	AT1G13280_AT3G43440	3	AT1G13280$\rightarrow$AT5G66880$\rightarrow$AT3G43440
AT1G32640	AT1G17420	1	RE	0.00073584	AT1G17420_AT1G48500	3	AT1G17420$\rightarrow$AT1G32640$\rightarrow$AT1G48500
AT1G32640	AT1G48500	1	PP	3.15E-06	AT1G17420_AT1G48500	3	AT1G17420$\rightarrow$AT1G32640$\rightarrow$AT1G48500
AT1G32640	AT1G17420	1	RE	0.00073584	AT1G17420_AT3G43440	3	AT1G17420$\rightarrow$AT1G32640$\rightarrow$AT3G43440
AT1G32640	AT3G43440	1	PP	3.15E-06	AT1G17420_AT3G43440	3	AT1G17420$\rightarrow$AT1G32640$\rightarrow$AT3G43440

## Discussion

Using the RWRtoolkit package, users can easily create and evaluate multiplex biological networks encoding multiple lines of interaction evidence. Importantly, RWRtoolkit is agnostic to organism, tissue, or condition. The user may apply these tools to non-model organisms by using orthologs of available networks from model organisms, or by building custom networks from experimental data as demonstrated here, or a combination of both.

RWRtoolkit uses RWR to explore and score topological connectivity from multiple lines of evidence between seed genes and other genes. In doing so, RWRtoolkit facilitates interpretation of a gene set beyond gene set enrichment analysis. It allows the user to expand the biological context of a gene or gene set using all lines available of evidence jointly. Moreover, the biological context and relationships within a gene set are explainable and quantifiable. Users can further explore the lines of evidence supporting gene-to-gene interactions (coexpression, protein–protein interactions, etc.) using Cytoscape or the KBase implementation.

RWRtoolkit was designed with ease of use in mind both for researchers familiar with the R software environment and those familiar with only command-line interfaces. Users who want to generate custom multiplex networks and use the entire suite of functions in RWRtoolkit can find the open-source code and vignettes on GitHub. For users who prefer a point-and-click GUI or have limited bioinformatic experience, we have included RWR_LOE and RWR_CV as applications within KBase and provided preassembled *Arabidopsis thaliana* multiplex networks.

### Addressing limitations in the RandomWalkRestartMH package

In addition to developing this suite of tools, we have updated the base underlying functionality of RandomWalkRestartMH. While RandomWalkRestartMH provides core functionality for constructing multiplex networks and implementing random walk algorithms, the construction of the multiplex was not optimized. As the size of networks and the total number of layers increases, the computational load for constructing the multiplex increases exponentially. With RWRtoolkit we parallelized this process to dramatically reduce the time to construct a large multiplex network from hours to seconds, thus enabling genome-wide multiplexes in higher organisms with tens of thousands of genes.

With respect to the tau parameter for weighting layers within the multiplex, the implementation in the RandomWalkRestartMH package was not correctly weighting the seeds within the multiplex network, leading Valdeolivas et al. [13] to conclude that tau had a minimal effect on output rankings. In that implementation tau incorrectly affects each seed’s weight equally across all layers rather than differently per layer. The impact is demonstrated in Fig. 4b, where changing tau fails to affect the RWR scores for nodes C and D which are present only in layer 2.

Comparatively, in our updated implementation, as we decrease tau for a given layer, we see a commensurate decrease in score for nodes existing only in that layer, illustrating that the tau parameter can play a measurable role with respect to node scoring. The corrected implementation gives users more flexibility to usefully weight the layers within their multiplexes.

### RWRtoolkit

#### Multiplex generation and netstats

The construction time of the multiplex network with 26,605 unique nodes and 918,640 edges demonstrates the computational efficiency and scalability of this parallelized method. This is especially relevant for research using larger, more complex networks.

The netstats functionality allows for users to concisely generate multiplex metadata and statistics and output those data to file. The basic statistics data provides a file output of useful context for interpreting the structural diversity of the layers within the multiplex, while the pairwise between layer jaccard similarity functionality offers a unique look at edge set comparisons. Fig. 5 illustrates that very few edges intersect between layers with respect to any two layers. This illustrates the heterogeneity of information encoded within the multiplex network, indicating that each layer is offering distinct biological aspects not encoded within the other layers.

#### Evaluation of multiplex networks using MAPMAN gene sets

One of the standout capabilities of RWRtoolkit is its ability to evaluate multiplex networks using gene sets known to have known biological connections and related topology, such as genes with shared annotation from GO or MAPMAN. These manually curated genes represent functionally cohesive sets derived from independent experimentation and databases, and provide a framework for testing a multiplex network’s predictive capabilities. The example cross-validation experiment conducted on our 9-layer comprehensive *Arabidopsis* multiplex demonstrates that functionally related groups of genes have strong topological similarity within this multiplex such that a subset of a functional gene set can be used to reliably predict functionally related genes with relatively few false positives. For comparison, the same analysis on rewired multiplex networks achieves no apparent predictive ability, illustrating that the networks are poorly constructed with respect to *Arabidopsis* genetic interactions.

#### Testing the connectivity of a set of genes

It is often the case in biological research that a list of genes will be generated from a statistical test or experiment such as GWAS or differential expression analysis. Using tools such as RWR_CV, we can explore how well these genes rank each other with respect to all others in the set, illustrating how topologically related those genes are given the layers in the multiplex network. Using a set of genes sharing the jasmonate signalling term from the MAPMAN database, we see a small example of the power of RWR_CV’s LOO method. Given that there are 30 genes within the set, 30 individual tests of RWR were run, leaving only one gene out each time. By then considering the mean rank and updated (rerank) ranks of all genes in the gene set, we can begin to understand how topologically related all genes in the gene set are to each other.

Twenty-seven of the 30 genes were annotated as jasmonate-related, and 23 of those achieved mean ranks in the top 100, demonstrating their close functional roles and topological similarity. Unsurprisingly, the three genes from non-jasmonate MAPMAN terms (AT4G33360, AT5G23010, and AT1G08380) ranked poorly with mean ranks of 2600, 9425, and 9707 because the gene set was dominated by jasmonate functionality. Such RWR_CV rankings can therefore be used to parse gene sets apart by function or to detect false-positive genes in a gene set [29]. Two biological case studies using RWR_CV functionality, one using the RWRtoolkit R package installation and the other using the KBase web application GUI, can be found as supplemental material.

RWRtoolkit was developed for applications to biological networks, but given that networks are domain agnostic and can signify any entity-to-entity relationship (including social networks, transportation networks, etc.), RWRtoolkit’s algorithms could be applied to any network data to identify highly ranked nodes using RWR.

#### Visualizing output

The web of connections within the multiplex network is overwhelming for meaningful interpretation of gene-to-gene relationships and biological context. RWRtoolkit, through commands such as RWR_CV, RWR_LOE, and shortest_paths, reduces the network down to a context of interest around seed genes and their high-ranking connections. The software offers multiple routes for visualizing these subnetsworks via the RCy3 API to Cytoscape or the KBase web app. We see this in Fig. 7a, where the top 50 genes from the run are plotted into Cytoscape and styled. This immediately allows for visual inspection of the seed genes, their topologically similar genes, and their connecting lines of evidence. For users leveraging the KBase GUI (Fig. 7b), web-based visualization tools allow for a seamless exploration of the gene scores, rankings, and inter- and intra-layer connections.

## Methods

All RWRtoolkit and RandomWalkRestartMH R software was run on the Andes compute system at the Oak Ridge Leadership Computing Facility (OLCF) at Oak Ridge National Laboratory (ORNL). The computations were run on a single compute node, with 2 AMD EPYC 7302 3 GHz 16-Core processors with 256 GB memory.

### RandomWalkRestartMH multiplex generation updates

We installed two separate instances of RandomWalkRestartMH: the original implementation defined by Valdeolivas et al. [13] installed as “RandomWalkRestartMHOriginal,” and our updated version. Both implementations of RandomWalkRestartMH define the same core functions for multiplex network processing: create.multiplex, which constructs the multiplex network list of igraph objects; compute.adjacency.matrix, which generates the supra-adjacency matrix for the multiplex; and compute.transition.matrix, which normalizes the adjacency matrix to create a transition matrix. We implemented a custom framework to evaluate the speed of these functions in multiplex generation.

We implemented a grid computation for generating multiplex networks of varying layer count *L*  $\in$ (10, 20, 30, 40, and 50) and node number per layer *N*  $\in$ (50, 100, 500, 1000, and 5000).

For each combination (*L,N*), we generated *L* random networks of size *N* using the Erdős–Rényi model in the igraph package [25,30]. These networks were then used to generate multiplex networks using both the previous implementation of RandomWalkRestartMH as well as our updated implementation. The updated implementation of RandomWalkRestartMH used 32 cores to compute the supra-adjacency matrix while the original implementation is single threaded.

### RandomWalkRestartMH tau updates

Valdeolivas et al. [13] noted that changing the tau parameter had minimal influence on RWR scores. Our investigation revealed an error within the original codebase so we updated our codebase to enable tau to weight layers appropriately.

To illustrate the impact of correctly operating tau we constructed a toy multiplex network consisting of 2 network layers - one layer a two-node path with nodes A and B and the other layer a clique of 4 nodes A, B, C, and D. We ran 100 iterations of Random.Walk.Restart.Multiplex function from RandomWalkRestartMH with nodes A and B as seeds while varying the values of tau for the second layer from 2 (maximum importance for this layer) to 0 (no importance for this layer).

### RWRtoolkit applications

#### Multiplex construction and layers

For our base application and public distribution, several *A. thaliana* multiplex networks were generated, including a comprehensive network with 9 layers. Network layer descriptions, sources, and sizes are provided in Table 6. All input networks were converted to be unweighted, and the multiplexes were constructed with a delta value of 0.5, where delta defines the global probability of the random walker transitioning between layers during an RWR iteration.

**Table 6:** A list of all network layers within the Comprehensive Multiplex Network.

Network layer	Description	Nodes	Edges
CoEvolution-DUO	Gene A connects to Gene B if an SNP in or near Gene A is correlated with an SNP in or near Gene B using the DUO metric [31]	2,283	13,514
Coexpression	Gene-Atlas Coexpression network obtained from AtGenie.org [32]	7,683	84,959
Knockout Similarity	Gene A connects to Gene B if the phenotypic effect of knocking out Gene A is similar to the phenotypic effect of knocking out Gene B [33]	1,841	94,952
PPI-6merged	GeneA connects to Gene B if their protein products have been shown to bind to interact with each other, typically through experimental evidence. The PPI-6merged network is the union of 6 different *A. thaliana* PPI networks: AraNet2 LC, AraNet2 HT [34], AraPPInet2 0.60 [35], BIOGRID 4.3.194 physical [36], AtPIN [37], and Mentha [38]	19,191	317,787
PEN-Diversity	Gene A connects to Gene B if the expression vector of Gene A is an important predictor of the expression vector of Gene B in an iRF model, where all other genes’ expression are included as covariates [5,39]	19,975	145,407
Predictive CG Methylation	Gene A connects to Gene B if the CG methylation vector of Gene A is an important predictor of the CG methylation vector of Gene B in an iRF model, where all other genes’ CG methylation states are included as covariates [5,39]	13,314	71,287
Regulation-ATRM	Gene A connects to Gene B if Gene A is a transcription factor (TF) that is shown to interact with Gene B (which may or may not be a TF). This dataset contains literature mined and manually curated TF regulatory interactions for *A. thaliana* [40]	789	1,359
Regulation-Plantregmap	This network contains computationally predicted TF-Target relationships based on motifs, binding sites, and ChipSeq data [41]	16,014	167,851
Metabolic-AraCyc	Gene A connects to Gene B if they are both enzymatic and are linked by a common substrate or product [42]	2857	21,524

For the comprehensive multiplex network, we used the RWR_netstats function to extract basic statistics using the option basic_statistics, and to determine the edge-wise overlap between layers using the pairwise_between_layers option and the default jaccard similarity score.

#### Network evaluation

To ensure the integrity of the multiplex network construction, we employed Random Walk with Restart Cross-Validation (RWR_CV) using a series of carefully curated gene sets known to exhibit high functional relatedness. These gene sets were based on shared MAPMAN annotations [28] that are curated by experts, and therefore represent functionally cohesive and biologically meaningful gene clusters. In a well-constructed multiplex network these genes are expected to be more topologically similar to each other than random genes would be, reflecting the robustness of the network’s structural integrity and its biological accuracy. We performed 5-fold (method = “kfold”) cross-validation (*k* = 5) for each MAPMAN-derived gene set with a restart value of 0.7 to evaluate the constructed multiplex network. The results from these evaluations were compared to those obtained from the same analysis conducted on 1,000 randomly rewired multiplex networks, each preserving the same number of nodes and edges across all layers.

#### Running a random walk on a multiplex

Users can obtain the random walk scores and ranks of all other genes within a multiplex network with respect to the seed gene of interest. We used the RWR_LOE function with a seed set file containing the seed genes of AT2G44810 and AT1G17420. These genes are part of a gene set with a shared MAPMAN ontology of jasmonate and were selected arbitrarily for the purproses of illustrating RWR_LOE functionality. The analysis was run with a restart value of 0.7 and cyto as 50 using the RWRtoolkit software package. Using the Kbase application, we loaded the Arabidopsis_thaliana genome in the Data section. The Arabidopsis_thaliana genome was loaded via the public database tab, using the NCBI RefSeq Genomes. We next selected the “Build FeatureSet from Genome” from the Comparative Genomics dropdown from the “Apps” section to add the cell into the Kbase Narrative. In the “Build FeatureSet from Genome” cell, we selected the Arabidopsis_thaliana genome, and searched and selected AT2G44810 and AT1G17420 in the Protein-Encoding Gene Feature text box. The output feature set was named and the cell was run. To run the random walk, the RWR_LOE cell was selected from the Comparative Genomics drop down within the Apps window. Within the RWR_LOE Cell, we selected the generated seed gene set from the “Seed Gene Keys” dropdown, selected the “Comprehensive Network” from the multiplex dropdown, chose 50 nodes as the maximum node rank to visualize, and named the output file object.

#### Testing the connectivity of a set of genes

It is possible that some genes within a gene set are more functionally related than others. For the sake of illustration, we selected a set of 27 genes with the shared MAPMAN ontology term “Jasmonate”. We also added genes AT1G08380, AT4G33360, and AT5G23010, from the MAPMAN ontology terms “Photosynthesis light reaction,” “Flavonoids,” and “Glucosinolates,” respectively. We then used RWR_CV to score how connected these 30 genes are to each other within the topology of the comprehensive multiplex network, using a restart value of 0.7 and the LOO CV option.

RWR_CV functionality and several pregenerated *Arabidopsis* multiplexes are also integrated into KBase as a GUI within the Comparative Genomics Apps. To define the gene set in KBase we used the “Build FeatureSet from Genome” option in the Comparative Genomics Apps section, selected the Arabidopsis_thaliana genome, and selected all 30 genes defined above. We then selected “Find Gene Set Interconnectivity using Cross Validation with RWRtoolsCV” from the Comparative Genomics dropdown and the comprehensive multiplex. From advanced parameters, we selected “loo” as the method of choice and chose 30 folds (as there are 30 genes within the input set).

#### Extracting the shortest paths

To see how those gene sets are connected within the multiplex network, all possible shortest paths between all seed and target genes were extracted using the RWRtoolkit application. We selected two genes as source genes (AT1G13280 and AT1G17420) and two genes as target genes (AT1G48500 and AT3G43440). We ran RWR_ShortestPaths with the source and target gene sets as parameters.

## Availability of source code and requirements

- Project Name: RWRtoolkit
- Project home page:  https://github.com/dkainer/rwrtoolkit
- Operating system(s):  Platform independent
- Programming language:  R
- Other requirements:  R 4.1.0 or higher
- License: GPL-3.0 license
- Software Heritage PID: swh:1:snp:0f97744911eaa2098a614537 ee7249bdcc513b3f

## Supplementary Material

giaf028_Supplemental_Files

giaf028_GIGA-D-24-00348_Original_Submission

giaf028_GIGA-D-24-00348_Revision_1

giaf028_Response_to_Reviewer_Comments_Original_Submission

giaf028_Reviewer_1_Report_Original_Submission Paul Pavlidis, Ph.D. -- 9/20/2024

giaf028_Reviewer_1_Report_Revision_1 Paul Pavlidis, Ph.D. -- 1/20/2025

giaf028_Reviewer_2_Report_Original_Submission Francis Agamah -- 10/11/2024

giaf028_Reviewer_2_Report_Revision_1 Francis Agamah -- 2/2/2025

## Data Availability

The datasets supporting the results of this article are available: Well-Watered Shoot Biomass GWAS Results from Supplemental Case Study can be found in Supplementary Table S19. KBase Narrative is publicly available at [43]. Snapshots of the code are available from Software Heritage [44]. Snapshots of the Pre-Assembled Arabidopsis Networks and MAPMAN-derived gene sets available from the GitHub repository [45] and from Software Heritage [46].

## Additional Files

**Supplementary Figure S1**: Differences among the methods of RWR_CV. (A) KFold: for the total number of folds within the set, *N* genes are divided by *K* folds, and randomly chunked without replacement into target sets of size *K*/*N*. (B) LOO: for each gene within the gene set, LOO uses that gene as a target with the remaining genes in the set used as seeds. (C) Singletons: for each gene within the gene set, Singletons uses that gene as a seed gene with the remaining genes in the set used as targets.

**Supplementary Figure S2**: Switchgrass GWAS Manhattan plots from one single locus (A; mixed linear model (MLM)) and three multilocus (BLINK, B; FarmCPU, C; and MLMM, D) using GAPIT. The solid line indicates significance at FDR $<$ 0.2, the dotted line indicates suggestive significance. Insets: *Q*–*Q* plots for each method.

**Supplementary Figure S3**: (A) Workflow diagram. Switchgrass GWAS models identify significant SNP associations with well-watered shoot biomass dry weight, and these SNPs are assigned to the two nearest Switchgrass genes. Switchgrass genes are converted to *Arabidopsis* orthologs, which are used as seeds in *Arabidopsis* multiplex networks. KBase apps were used to explore highly interconnected genes and identify functional context of the GWAS genes using a 9-layer multiplex, including 3 network layers (DUO Co-Evolution, iRF Predictive Expression, and iRF Predictive CG Methylation) derived from high-performance computing using models with petascale- or exascale-level combinatorial complexity. Figure created using Biorender.com. (B) Using KBase, RWR-CV identified three seed genes (inset, red box) that were unconnected from the top 200 ranked genes. Seed genes (*Arabidopsis* orthologs from GWAS results) are displayed as purple nodes, and teal to light blue nodes are color scaled based on RWR-LOE ranks. Edge colors indicate the line of evidence from which each gene–gene relationship was derived. (C) Visualization of KBase RWR-LOE top-200 rank output using orthologs of genes that were highly interconnected based on RWR-CV (“filtered genes”). Inset: magnified view of network. When hovering over a gene, users can view Gene Ontology (GO), knockout phenotype, and MAPMAN annotations for that gene. (D) Framework for a conceptual model of Switchgrass well-watered shoot biomass with GWAS genes (red) and top-200 ranked genes by RWR-LOE (black). Genes from GWAS and RWR-LOE ranks implicated shoot apical meristem development, homeodomain transcription factors involved in transcriptional repression, and sphingolipid/ceramide synthesis. Figure made with BioRender.com.

**Supplementary Figure S4**: Illustrating RWR_LOE Rank Differential between pennycress dual knockout experiments FAE1/FAD2 and FAE1/ROD1. The top-200 ranked genes from RWR_LOE of FAE1/FAD2 (*x*-axis, left) and FAE1/ROD1 (*x*-axis, right) are shown plotted against their corresponding ranks (*y*-axis) from RWR_LOE of FAE1/ROD1 and FAE1/FAD2, respectively. Lower numbers mean better ranking. The top-200 genes for one knockout pair generally achieve a similar ranking in the other knockout pair (blue dots). However, within each experiment, there exist genes that rank far worse when compared to their corresponding rank in the other experiment (orange dots).

**Supplementary Figure S5**: Top-ranked genes identify overlapping and distinct biological processes in pennycress from alternative knockout gene pairs. (A) ClueGO enrichment of the FAE1/FAD2-specific sets of top-ranked genes. A bipartite network exists in which nodes are genes and enrichment terms, and edges connecting genes to terms indicate enrichment. (B) Shortest paths between the FAE1/FAD2 gene set and EMB3113. The shortest path found from the FAD2 to EMB3113 uses edges from the predictive expression network while the path from FAE1 to EMB3113 uses edges from the gene atlas and duo network. In cases where edges exist on multiple layers, multiple labeled edges will exist between the two nodes.

**Supplementary Table S1**: Example network layer edge list with no edge weights to be used as input.

**Supplementary Table S2**: Example network layer edge list with edge weights to be used as input.

**Supplementary Table S3**: Example flist in which each row has a full path to an edge list and an associated layer name.

**Supplementary Table S4**: RWR Gene Sets example file.

**Supplementary Table S5**: Example RWR_netstats Basic Statistics Output.

**Supplementary Table S6**: RWR_netstats pairwise_between_mpo_layer_jaccard.tsv.

**Supplementary Table S7**: RWR_netstats pairwise_between_mpo_layer_overlap.tsv.

**Supplementary Table S8**: RWR_Netstats Multiplex Layers to Ref Net Jaccard example file output.

**Supplementary Table S9**: RWR_Netstats Multiplex Layers to Ref Net overlap example file output.

**Supplementary Table S10**: RWR_Netstats net to net similarity example file output.

**Supplementary Table S11**: RWR_Netstats calculated tau example file output.

**Supplementary Table S12**: RWR_Netstats calculate exclusivity file output.

**Supplementary Table S13**: RWR_LOE output example file output

**Supplementary Table S14**: 14. RWR_CV Full Ranks example file output.

**Supplementary Table S15**: RWR_CV Mean Ranks example file output.

**Supplementary Table S16**: RWR_CV Metrics example file output.

**Supplementary Table S17**: RWR_CV Summary example file output.

**Supplementary Table S18**: RWR_shortestpaths example file output.

**Supplementary Table S19**: Outer Join of the top 200 ranked nodes from fae1 fad2 and top 200 ranked nodes from fae1 rod1 using RWR_LOE.

## Abbreviations

AIG1: avirulence induced gene; AS2: asymmetric leaves 2; BLUPS: best linear unbiased predictors; BP: biological process; CV: cross-validation; EMB3113: embryo defective 3113; FAD2: fatty acid desaturase 2; FAE1: fatty acid elongation 1; GO: Gene Ontology; GWAS: genome-wide association study; HD-ZIP III: Homeodomain Leucine-Zipper; JA: jasmonic acid; KAN1: KANADI1; LCBs: long-chain bases; LOE: line of evidence; PIN1: pin-formed 1; PHB: PHABULOSA; PHV: PHAVOLUTA; PPI: protein–protein interactions; ROD1: reduced oleate desaturation 1; RWR: random walk with restart; SLD1: sphingoid LCB desaturase 1; SLD2: sphingoid LCB desaturase 2; SNP: single nucleotide polymorphism; TF: transcription factor; QTL: quantitative trait locus; WOX9: Wuschel-related homeobox 9.
