# Supplementary material for: RWRtoolkit: multi-omic network analysis using random walks on multiplex networks in any species
Source: Gigascience. 2025 Apr 24;14:giaf028. doi: 10.1093/gigascience/giaf028 (PMC12020474; doi:10.1093/gigascience/giaf028)
Supplement: giaf028_Supplemental_Files [file giaf028_supplemental_files.zip › RWRtoolkit_GigaScience_SI_Revision.pdf]

Supplemental Material

RWR_CV Metric Calculations

Left out genes in the RWR_CV runs act as target genes (T), and are considered true positives (TP). All non-target genes (i.e. all genes in the multiplex not in the seed set) act as false positives (FP). As the genes increase in rank, precision and recall at rank R are considered.

$$Precision@R = \frac{TP@R}{TP@R + FP@R} \equiv Precision@R = \frac{TP@R}{R}$$

$$Recall@R = \frac{TP@R}{|TP|}$$

Precision at R is defined by the true positive at rank R divided by the total number of true positives at R and false positives at R. As there are no false negative or true negative data to consider, we can also define precision at R to be the total number of true positives at R divided by the rank R (as the sum of all true positives and false positives will equal the rank R). To calculate recall at R, we consider the total number of true positives at R divided by the total number of true positives within the gene set. Mean rank is calculated for each gene G across K folds. For final output, genes are reranked with respect to mean ranks.

Case Studies

We used RWRtoolkit to explore genetic relationships in two promising biofuel crops: switchgrass (Panicum virgatum) and pennycress (Thlaspi arvense). Since large scale multi-omic pennycress and switchgrass data are not yet available, we used the Comprehensive multiplex network from varied data layers available for Arabidopsis thaliana, a model species which is in the same family as pennycress (Table 2).

Results

We used RWRtoolkit to explore genetic relationships in two promising biofuel crops: switchgrass (Panicum virgatum) and pennycress (Thlaspi arvense). Since large scale multi-omic pennycress and switchgrass data are not yet available, we used the Comprehensive multiplex network from varied data layers available for Arabidopsis thaliana, a model species which is in the same family as pennycress (Table 2).

Identifying Genes Contributing to Switchgrass Well-Watered Shoot Biomass Using RWRtoolkit and KBase

Identifying genetic variants, genes, and biological pathways controlling plant biomass can prioritize gene targets for improving biofuel feedstocks. To this end, we performed a genome-wide association study (GWAS) for well-watered shoot biomass dry weight (Supplemental Table 19, Supplemental Fig 2.) in the tetraploid bioenergy feedstock switchgrass (Panicum virgatum). Two GWAS models (BLINK and FarmCPU) identified 22 unique significant single nucleotide polymorphisms (SNPs) associated with well-watered shoot biomass at an FDR < 0.2. These were mapped to a total of 38 unique switchgrass genes based on genomic proximity.

We next wanted to understand the biological context among our Switchgrass well-watered shoot biomass gene set. However, there are limited publicly available networks that describe gene-gene relationships in switchgrass [1, 2], so we mapped the 38 switchgrass GWAS genes to 32 Arabidopsis (Arabidopsis thaliana) orthologs using Phytozome [3]. For a greater understanding of the functional context of these GWAS results we leveraged our Arabidopsis multiplex network consisting of nine distinct lines of biological evidence to explore the relationships among these orthologs (Supplemental Figure 3A).

RWR_CV and RWR_LOE functionality and the Arabidopsis multiplex network were incorporated into the DOE Systems Biology Knowledgebase (KBase [4]) interface (Supplemental Figure 3B-C). sing the visualization derived from the KBase tools, we first removed three orthologs (AT1G12640, AT1G47310, AT3G14470) that were not connected to any other GWAS genes, nor to the top 200 genes ranked by RWR_CV with 5-fold cross validation (Supplemental Figure 3B). We then used the remaining 29 GWAS gene orthologs as seeds for the RWR_LOE application to help generate a framework for a conceptual model depicting the top candidate genes from GWAS and their functional context derived from other high ranking genes (Supplemental Figure 3D). Here, we focus our discussion on two genes identified by shoot biomass GWAS and their relevant RWR_LOE connections.

Both BLINK and FarmCPU GWAS models identified the SNP Chr02K_40684207 as significant, with FDR-adjusted p-values of 1.00E-5 and 8.38E-13, respectively (Supplemental Table 19, Supplemental Fig. 2). This SNP is located in the switchgrass gene Pavir.2KG286800, which is orthologous to the Arabidopsis gene SPHINGOID LCB DESATURASE 2 (SLD2, AT2G46210.1) (Supplemental Table 19). SLD2 plays a crucial role in sphingolipid biosynthesis by catalyzing the desaturation of long-chain bases (LCBs) at position 8 [5]. RWR_LOE analysis revealed interactions between SLD2 and SPHINGOID LCB DESATURASE 1 (SLD1, AT3G61580) and DES-1-LIKE (AT4G04930), which are delta 8 and delta 4 desaturases through the PPI layer (Supplemental Fig. 3D), as well as AGL18, a MADS-domain transcription factor (AT3G57390) through the coexpression layer.

Additionally, the BLINK GWAS model identified Chr025_34638064 as significant (FDR-adjusted p-value 1.02E-06, Supplemental Table 19).

The nearest gene to this SNP is Pavir.2KG303000, which is orthologous to AT5G16560.1, or KANADI1 (KAN1). KAN1 is a transcription factor (TF) with a significant role in adaxial-abaxial (top and bottom) polarity in leaves and the proper development of the shoot apical meristem (SAM) via auxin signaling through interactions with auxin-related genes [6]. Previous work implicated KAN1 in a growth-defense tradeoff regime in Arabidopsis thaliana through jasmonic acid (JA) signaling [7]. The authors reported that JA activates KAN1 which suppresses auxin biosynthesis, transport, and signaling, ultimately inhibiting growth [7]. Importantly, this suggests that SNPs affecting KAN1 may alter growth and biomass phenotypes. To gain a more comprehensive understanding of the mechanisms involved in this regulatory network, we examined the genes connected to KAN1 in the KBase RWR_LOE Narrative.

Visualizations of the lines of evidence around KAN1 and within multiplex network revealed protein-protein interactions (PPI-6merged, Table 2) between products of KAN1 and PHAVOLUTA (PHV), PHABULOSA (PHB), and WUSCHEL-RELATED HOMEOBOX 9 (WOX9; Supplemental Fig. 3D) [8]. KAN1 was also connected to PHV, PHB, ASYMMETRIC LEAVES 2 (AS2) and PIN-FORMED 1 (PIN1) through TF regulatory interactions (Regulation-ATRM, described in Table 2). Additionally, the results of RWR_LOE exhibited a machine learning-predicted epigenetic relationship (Predictive CG Methylation, Table 2) between KAN1 and WOX9. Together, the output of RWR_CV and RWR_LOE identified shoot apical meristem development, long-chain fatty acid modifications, and homeodomain transcription factors as strong candidates affecting shoot biomass.

Predicting the Functional Effects of Gene Edits

Next, we applied RWRtoolkit to explore biological pathways surrounding two distinct genetically modified lines of pennycress (Thlaspi arvense). Pennycress is a cover crop in the Brassicaceae family with great potential to produce biodiesel and sustainable aviation fuel through large seed yields containing high volumes of long-chain fatty-acids. Jarvis et al. [9] recently demonstrated that pennycress lines with dual knockout of genes FAE1 and FAD2 produced seeds with 91

We ran RWR_LOE for each knockout pair separately and noted that many of the top 200 ranked genes from the FAE1/ROD1 run remained very highly ranked in the FAE1/FAD2 run. However, some of the top 200 genes fell drastically in ranking, indicating that they were no longer part of the same functional context after swapping ROD1 for FAD2 in the LOE runs (Illustrated in Supplemental Figure 4). Such "differentially ranked" genes can be considered as candidates driving functional changes that result in the phenotypic differences observed between the two dual knockout lines.

Using the set differential methodology to illustrate these differential rank differences, Gene Ontology enrichment of the intersection of the top 200 ranked genes from the FAE1/FAD2 seeds and the FAE1/ROD1 seeds (i.e., genes ranked highly by RWR_LOE for both knockout pairs) exhibited enrichment for fatty acid biosynthesis, fatty acid metabolic process, and sphingolipid metabolic process. Genes ranked highly by RWR_LOE for FAE1/ROD1 but not for FAE1/FAD2 showed three terms enriched: seed oil biogenesis, response to freezing, and lipid storage. The genes ranked highly by RWR_LOE for FAE1/FAD2 but not for FAE1/ROD1, however, showed enrichment for multiple GO BP and KEGG terms beyond those expected for lipids, including photoinhibition, regulation of circadian rhythm, and chloroplast rRNA processing, illustrated in Supplemental Figure 5 A.

To better understand the connectivity between FAE1 and FAD2 to EMB3113, we used RWR_ShortestPaths to explore the connections between these genes of interest. FAE1 is connected to EMB3113 through AT2G34315 via a GeneAtlas co-expression edge, and from AT2G34315 to AT3G61920 via a DUO computed similarity (an advanced correlation metric) [10] edge. Finally, it connects EMB3113 from AT3G61920, also via the DUO similarity metric (Supplemental Figure 5 B). FAD2 connects to EMB3113 via a PEN (Predictive Expression Network) edge with AT1G09750 .

Discussion

Here, we demonstrate that RWRtoolkit enables the discovery of gene-to-gene relationships not previously apparent within a monoplex network topology, as well as gene-to-gene relationships across the broader multiplex surrounding a gene set of interest. Using the RWRtoolkit package, users can create and validate multiplex biological networks encoding multiple lines of evidence. Importantly, RWRtoolkit is agnostic to organism, tissue or condition. The user may explore biological pathways in non-model organisms by either using orthologs and available networks from model organisms as demonstrated in the present work by building custom networks from experimental data, or a combination of both approaches. In addition to obtaining topologically relevant gene-to-gene relationships from the multiplex networks, users can identify the lines of evidence driving these interactions (e.g., co-expression, protein-protein interactions, etc.) using Cytoscape or KBase. Additionally, peta/exascale-complexity networks derived from AI-based methods can be used as layers within the multiplex to identify relationships for poorly-annotated genes, including proteins of unknown function.

RWRtoolkit was designed as a user-friendly package for researchers familiar with R software and command line interfaces. Users who want to generate custom multiplex networks and use the entire suite of functions in RWRtoolkit can find the open-source code and vignettes on GitHub. For users who prefer a point-and-click graphical user interface or have limited bioinformatic experience, we have included RWR_LOE and RWR_CV as applications within KBase and provided pre-assembled Arabidopsis thaliana multiplex networks.

RWRtoolkit explores topological connectivity between seed genes and other genes based on multiple lines of evidence. In doing so, RWRtoolkit facilitates interpretation of a gene set outside of gene set enrichment analysis, with the goal of expanding the biological context of genes in a gene set which may not have been previously studied in the same experimental context. Moreover, the biological context between any group of genes is explainable based on the various types of biological evidence present in a multiplex network.

Well Watered Shoot Biomass GWAS Results

We applied the KBase RWR_LOE application to functionally contextualize GWAS results from the bioenergy feedstock switchgrass, as GWAS results for complex traits are often difficult to interpret because significant SNPs can map to a set of genes with largely uncharacterized relationships. RWR_LOE captured connections surrounding both sphingolipid production and a regulatory subnetwork of cell differentiation and specification that likely affects vascular development in the SAM. In addition, the interactions captured by RWR-LOE led to the development of a conceptual model framework highlighting these findings (Fig. 4 D).

Sphingolipids are integral to various cellular, developmental, and stress-related processes [11]. Though the SNP associated with SLD2 was identified by two GWAS models, SLD2 knockout experiments in A. thaliana showed no phenotype growth defects under normal conditions [5]. However, double mutants of sld1 sld2 exhibited altered growth phenotypes under cold stress conditions accompanied by changes in the distribution of complex sphingolipids such as glucosyl-ceramide (GluCer) and GIPCs [5]. These findings suggest an ambiguous role for SLD2 in shoot biomass accumulation. However, AGL18, which was connected to SLD2 via coexpression, is essential in regulating the transition from vegetative to reproductive growth in plants [12] suggesting its involvement in shoot biomass development. Notably, the additional context provided by RWR_LOE highlights a connection between SLD2 and shoot biomass accumulation, a relationship that was not apparent from the GWAS results alone.

With respect to the SAM, the abaxial-adaxial regulatory network involved in shoot patterning and vascular development is primarily controlled by the KANADI TFs as well as the the Class III Homeodomain Leucine-Zipper (HD-ZIP III) TFs, PHB and PHV [13]. KAN1 engages in both direct protein-protein interactions with PHB and PHV and influences their transcriptional activities antagonistically to preserve the required abaxial/adaxial boundary in apical meristem establishment and leaf development [14, 15]. AS2 is an important LOB-domain containing an adaxial regulator required for symmetrical leaf expansion [16]. AS2 and KAN1 are mutual transcriptional repressors controlling the lateral expansion and flatness of leaves which is fundamental to proper vegetative growth in the SAM [17]. Similarly, WOX9 is a WUS homeobox-containing TF required for growth and maintenance of the vegetative SAM, in part through maintaining the population of undifferentiated stem cells [18]. KAN1 engages in a protein-protein interaction with WOX9 , likely to balance stem cell maintenance and cell fate/identity during vascular development in the SAM [8, 19]. The methylation state of KAN1 was found to be an important predictor of the methylation state of WOX9, suggesting an epigenetic relationship between these regulators in the SAM. KAN1 also regulates key auxin-transport genes, such as PIN1, to orchestrate organ patterning and vascular development in the SAM. Specifically, KAN1 directly inhibits PIN1 by binding to a specific site downstream of PIN1, effectively restrict-

ing auxin flow by PIN1 repression [14]. Additionally, the protein products of WOX9, PHB, and PHV were all shown to interact via a protein-protein interaction layer of the multiplex, further indicating a tightly interconnected regulatory network among these genes.

RWR_LOE significantly enhances traditional GWAS by uncovering additional topological connections within a multiplex (or monoplex) that would otherwise remain unknown. This approach enabled us to construct a conceptual model that includes a network of genetic influences on shoot biomass, which would not be possible with the GWAS results alone. We demonstrated how users can leverage the capabilities of RWR_LOE across a network to reveal mechanistic interactions and interpretations surrounding a user-defined gene set.

Exploring Dual Knockouts with RWR_LOE

We used RWRtoolkit with multi-omic Arabidopsis networks to understand the functional difference between FAE1/ROD1 and FAE1/FAD2 knockout plants from pennycress gene editing experiments. Using RWR_LOE, we aimed to explain the observed phenotypic differences between these genotypes based on differential network connectivity. The genes found in common in the top 200 ranks for both FAE1/ROD1 and FAE1/FAD2 RWR_LOE runs exhibited enrichment for fatty acid biosynthesis-related GO and KEGG terms, which was expected given the well-described function of the three targeted genes in fatty acid biosynthesis and modification. The FAE1/ROD1 specific subnetwork (i.e., genes not found in the FAE1/FAD2 run) did not capture any obvious function beyond additional terms related to fatty acid synthesis and storage. However, the enriched GO and KEGG terms for the FAE1/FAD2 subnetwork suggest impacts to the growth and development of the plant, and are possibly affected in a regulatory manner when FAE1 and FAD2 are knocked out, but not when FAE1 and ROD1 are knocked out. AT2G33800 (EMB3113), rank 160 in the FAE1/FAD2 run, is annotated with the enriched GO BP term "chloroplast rRNA processing," and has been shown to express a reduction in growth compared to wild type [20], offering a potential explanation as to why there exists growth reduction in the FAE1/FAD2 knockout line.

Using RWRtoolkit's RWR_LOE and RWR_ShortestPaths functionality to explore the surrounding network topology of these knockout gene pairs, we start to gain some potential insights as to why the FAE1/FAD2 dual knockout showed deleterious effects upon the growth of pennycress. Interestingly, within the path connecting FAE1 to EMB3113, AT2G34315 acts as an avirulence induced gene (AIG1)[21] and AT3G61920 encodes a PADRE protein that exhibits downregulation when exposed to Pst DC3000 AvrRPS4 [22]. Therefore, genes within this path may suggest a connection between the growth/defense tradeoff in plant development. AT1G09750, the connecting gene between FAD2 and EMB3113, encodes a metabolic enzyme with roles in hydrotropic response signal transduction and osmotic equilibrium maintenance [23]. Additionally, AT1G09750 has been quantified as being expressed during active growth and growth arrest developmental stages of Arabidopsis hypocotyls. Given AT1G09750's role during active growth stages of development, we can begin to paint a more comprehensive picture as to why these pleiotropic effects occurred.

While the present manuscript focuses on gene-gene homogeneous networks, users could expand RWRtoolkit to include additional omic data such as metabolite-metabolite networks as well as heterogeneous networks (phenotype-gene, phenotype-metabolite, etc.). The focus of RWRtoolkit is intended to be applied to biological networks, but given that networks are domain agnostic and can signify any entity to entity relationship (including social networks, transportation networks, etc.), RWRtoolkit's algorithms could be applied to any network data to identify highly ranked nodes using random walk with restart.

Together, we show that RWRtoolkit is an easy-to-use software package and KBase application that facilitates biological interpretation of experimental data sets using network analyses. We hope this package will provide another useful tool for researchers to interpret functional context from newly derived experimental data in order to accelerate scientific discovery.

Methods

GWAS

SNP Variant Calling and Filtering

Variant calling methods for the SNPs were described previously [24]. Briefly, Illumina HiSeq X10 and Illumina NovaSeq 6000 paired-end sequencing at Department of Energy Joint Genome Institute and the HudsonAlpha Institute for Biotechnology were used for whole genome re-sequencing of the 260 P. virgatum genotypes. The median sequencing depth was 59x. The raw SNP dataset was filtered down to 4,458,778 SNPs for GWAS and all other downstream analyses: SNPs with more than 10

Phenotyping

The aboveground shoot dry biomass was measured on 1442 P. virgatum plants (298 unique genotypes) grown under well-watered conditions in a greenhouse in multiple batches. Phenotypic outliers in the dataset were removed using the Median Absolute Deviation (MAD) method [25] with a MAD distance of 6 used as a threshold for removing outliers. Best Linear Unbiased Predictors (BLUPs) [26] of each genotype were obtained by running a linear model with genotypes as the random effect and the Batch as the fixed effect (covariate).

GWAS

Association of the SNPs in the genome with the phenotypic trait (BLUPs) were calculated using GAPIT version 3 R package [27] with the following GWAS models: MLM [28], MLMM [29], FarmCPU [30], and BLINK [31]. The SNPs from the association test that passed the FDR threshold of 0.2 were considered significant. The significant SNPs were mapped to the two nearest Panicum virgatum genes, upstream and downstream using Version 5.1 snpEff annotation [15].

Exploring Gene Edits with RWR_LOE

Differential Ranking Between Two Gene Sets

We ran RWR_LOE two separate times: first with a seed gene set containing FAE1 (AT4G34520) and ROD1 (AT3G15820), then with a seed gene set containing FAE1 and FAD2 (AT3G12120). Each individual run produced rankings for all 26,605 genes in the multiplex network. We extracted the top 200 ranked genes from running RWR_LOE on the FAE1/ROD1 gene pair, and contrasted those to the ranks obtained for those 200 genes when running RWR_LOE on the FAE1/FAD2 gene pair.

Cytoscape Set Differential

As in the differential ranking analysis, RWR_LOE was run using seeds FAE1 and FAD2, and then using FAE1 and ROD1 as seeds for a separate ranking analysis. Here, the subnetworks containing the seeds and the top 200 ranked genes were extracted by using the cyto parameter (–cyto 200), generating two separate networks in Cytoscape, named FAE1.FAD2, and FAE1.ROD1. By subtracting the FAE1.ROD1 network from the FAE1.FAD2 network using the difference method in Cytoscape, we obtained edges unique only to the FAE1/FAD2 RWR_LOE rankings, resulting in the FAD2 specific network. Conversely, by subtracting the FAE1.FAD2 network from the FAE1.ROD1 network, we obtained edges unique only to FAE1/ROD1 RWR_LOE rankings, resulting in a ROD1 specific network. Nodes

shared by both networks were obtained via the intersect method in Cytoscape, creating the intersection subnetwork.

GO Enrichments

Gene set enrichment was run to assess biological functionality for all three distinct subnetworks using ClueGO, obtained from the Cytoscape App Store [32, 33]. For each individual network (FAD2 and ROD1 specific networks and the intersection network), all gene nodes within each network were loaded into the Load Marker List(s) section and enriched using ClueGO in Functional Analysis mode within the Load Marker List section. The GO Biological Process and KEGG Ongologies/Pathways were selected within the ClueGO settings.

RWR Shortest Paths

To extract shortest paths between the source genes (FAE1, FAD2) and the target gene (EMB3113), we ran RWR_ShortestPaths supplying the source gene set and the target gene set with the cyto parameter as true (–cyto TRUE). The output file contains an edge list with additional metadata for each edge, comprising the shortest paths from all nodes in the source gene set to all nodes in the target gene set.

References

1. Rao X, Chen X, Shen H, Ma Q, Li G, Tang Y, et al. Gene regulatory networks for lignin biosynthesis in switchgrass (Panicum virgatum). Plant Biotechnol J 2019 Mar;17(3):580–593.
2. Zhang P, Duo T, Wang F, Zhang X, Yang Z, Hu G. De novo transcriptome in roots of switchgrass (Panicum virgatum L.) reveals gene expression dynamic and act network under alkaline salt stress. BMC Genomics 2021 Jan;22(1):82.
3. Goodstein DM, Shu S, Howson R, Neupane R, Hayes RD, Fazo J, et al. Phytozome: a comparative platform for green plant genomics. Nucleic Acids Res 2012 Jan;40(Database issue):D1178–86.
4. Arkin AP, Cottingham RW, Henry CS, Harris NL, Stevens RL, Maslov S, et al. KBase: The United States Department of Energy Systems Biology Knowledgebase. Nat Biotechnol 2018 Jul;36(7):566–569.
5. Chen M, Markham JE, Cahoon EB. Sphingolipid 8 unsaturation is important for glucosylceramide biosynthesis and low-temperature performance in Arabidopsis. Plant J 2012 Mar;69(5):769–781.
6. Ram H, Sahadevan S, Gale N, Caggiano MP, Yu X, Ohno C, et al. An integrated analysis of cell-type specific gene expression reveals genes regulated by REVOLUTA and KANADI1 in the Arabidopsis shoot apical meristem. PLoS Genet 2020 Apr;16(4):e1008661.
7. Zhang N, Zhao B, Fan Z, Yang D, Guo X, Wu Q, et al. Systematic identification of genes associated with plant growth-defense tradeoffs under JA signaling in Arabidopsis. Planta 2020 Jan;251(2):43.
8. Arabidopsis Interactome Mapping Consortium. Evidence for network evolution in an Arabidopsis interactome map. Science 2011 Jul;333(6042):601–607.
9. Jarvis BA, Romsdahl TB, McGinn MG, Nazarenus TJ, Cahoon EB, Chapman KD, et al. CRISPR/Cas9-Induced and Mutations Stacked With Confer High Oleic Acid Seed Oil in Pennycress (L.). Front Plant Sci 2021 Apr;12:652319.
10. Climer S, Templeton AR, Garvin M, Jacobson D, Lane M, Hulver S, et al. Synchronized genetic activities in Alzheimer's brains revealed by heterogeneity-capturing network analysis. bioRxiv 2020 Jan;p. 2020.01.28.923730.
11. Mamode Cassim A, Grison M, Ito Y, Simon-Plas F, Mongrand S, Boutté Y. Sphingolipids in plants: a guidebook on their function in membrane architecture, cellular processes, and environmental or developmental responses. FEBS Lett 2020 Nov;594(22):3719–3738.
12. Adamczyk BJ, Lehti-Shiu MD, Fernandez DE. The MADS domain factors AGL15 and AGL18 act redundantly as repressors of the floral transition in Arabidopsis. Plant J 2007 Jun;50(6):1007–1019.
13. Barton MK. Twenty years on: the inner workings of the shoot apical meristem, a developmental dynamo. Dev Biol 2010 May;341(1):95–113.
14. Merelo P, Xie Y, Brand L, Ott F, Weigel D, Bowman JL, et al. Genome-wide identification of KANADI1 target genes. PLoS One 2013 Oct;8(10):e77341.
15. Emery JF, Floyd SK, Alvarez J, Eshed Y, Hawker NP, Izhaki A, et al. Radial patterning of Arabidopsis shoots by class III HD-ZIP and KANADI genes. Curr Biol 2003 Oct;13(20):1768–1774.
16. Iwakawa H, Iwasaki M, Kojima S, Ueno Y, Soma T, Tanaka H, et al. Expression of the ASYMMETRIC LEAVES2 gene in the adaxial domain of Arabidopsis leaves represses cell proliferation in this domain and is critical for the development of properly expanded leaves. Plant J 2007 Jul;51(2):173–184.
17. Wu G, Lin WC, Huang T, Poethig RS, Springer PS, Kerstetter RA. KANADI1 regulates adaxial-abaxial polarity in Arabidopsis by directly repressing the transcription of ASYMMETRIC LEAVES2. Proc Natl Acad Sci U S A 2008 Oct;105(42):16392–16397.
18. Wu X, Dabi T, Weigel D. Requirement of homeobox gene STIMPY/WOX9 for Arabidopsis meristem growth and maintenance. Curr Biol 2005 Mar;15(5):436–440.
19. Tvorogova VE, Krasnoperova EY, Potsenkovskaia EA, Kudriashov AA, Dodueva IE, Lutova LA. What Does the WOX Say? Review of Regulators, Targets, Partners. Mol Biol 2021 May;55(3):311–337.
20. Mateo-Bonmatí E, Casanova-Sáez R, Quesada V, Hricová A, Candela H, Micol JL. Plastid control of abaxial-adaxial patterning. Sci Rep 2015 Nov;5:15975.
21. Dekkers BJW, Pearce S, van Bolderen-Veldkamp RP, Marshall A, Widera P, Gilbert J, et al. Transcriptional dynamics of two seed compartments with opposing roles in Arabidopsis seed germination. Plant Physiol 2013 Sep;163(1):205–215.
22. Didelon M, Khafif M, Godiard L, Barbacci A, Raffaele S. Patterns of Sequence and Expression Diversification Associate Members of the PADRE Gene Family With Response to Fungal Pathogens. Front Genet 2020 May;11:491.
23. Miao R, Wang M, Yuan W, Ren Y, Li Y, Zhang N, et al. Comparative Analysis of Arabidopsis Ecotypes Reveals a Role for Brassinosteroids in Root Hydrotropism. Plant Physiol 2018 Apr;176(4):2720–2736.
24. Lovell JT, MacQueen AH, Mamidi S, Bonnette J, Jenkins J, Napier JD, et al. Genomic mechanisms of climate adaptation in polyploid bioenergy switchgrass. Nature 2021 Feb;590(7846):438–444.
25. Leys C, Ley C, Klein O, Bernard P, Licata L. Detecting outliers: Do not use standard deviation around the mean, use absolute deviation around the median. J Exp Soc Psychol 2013 Jul;49(4):764–766.
26. Henderson CR. Best linear unbiased estimation and prediction under a selection model. Biometrics 1975 Jun;31(2):423–447.
27. GAPIT Version 3: Boosting Power and Accuracy for Genomic Association and Prediction. Genomics Proteomics Bioinformatics 2021 Aug;19(4):629–640.
28. Yu J, Pressoir G, Briggs WH, Vroh Bi I, Yamasaki M, Doebley JF, et al. A unified mixed-model method for association mapping that accounts for multiple levels of relatedness. Nat Genet 2006 Feb;38(2):203–208.
29. Segura V, Vilhjálmsson BJ, Platt A, Korte A, Seren Long Q, et al. An efficient multi-locus mixed-model approach for genome-wide association studies in structured populations. Nat Genet 2012 Jun;44(7):825–830.
30. Liu X, Huang M, Fan B, Buckler ES, Zhang Z. Iterative Us-

age of Fixed and Random Effect Models for Powerful and Efficient Genome-Wide Association Studies. PLoS Genet 2016 Feb;12(2):e1005767.
31. Huang M, Liu X, Zhou Y, Summers RM, Zhang Z. BLINK: a package for the next level of genome-wide association studies with both individuals and markers in the millions. Gigascience 2019 Feb;8(2).
32. Bindea G, Mlecnik B, Hackl H, Charoentong P, Tosolini M, Kirilovsky A, et al. ClueGO: a Cytoscape plug-in to decipher functionally grouped gene ontology and pathway annotation networks. Bioinformatics 2009 Apr;25(8):1091–1093.
33. Lotia S, Montojo J, Dong Y, Bader GD, Pico AR. Cytoscape app store. Bioinformatics 2013 May;29(10):1350–1351.

Supplemental Figures

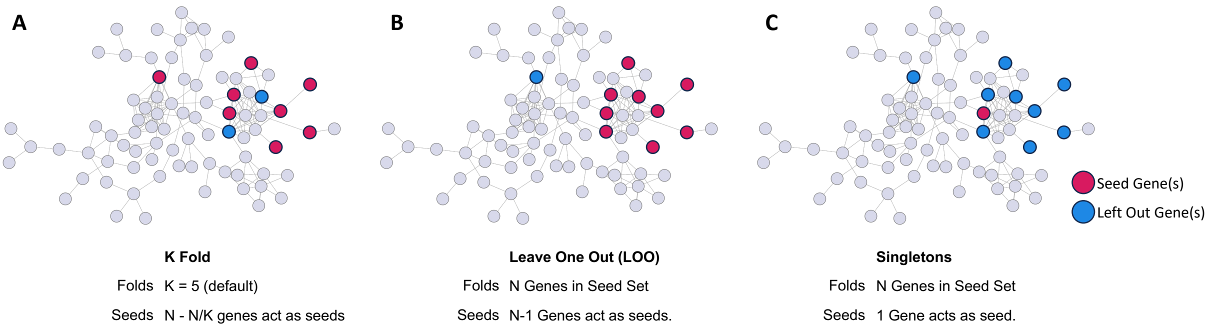

Figure 1. Differences among the methods of RWR_CV. A. K Fold: For the total number of folds within the set, N genes are divided by K folds, and randomly chunked without replacement into target sets of size K / N. **B. Leave One Out (LOO)**: For each gene within the gene set, LOO uses that gene as a target with the remaining genes in the set used as seeds. **C.** Singletons: For each gene within the gene set, Singletons uses that gene as a seed gene with the remaining genes in the set used as targets.

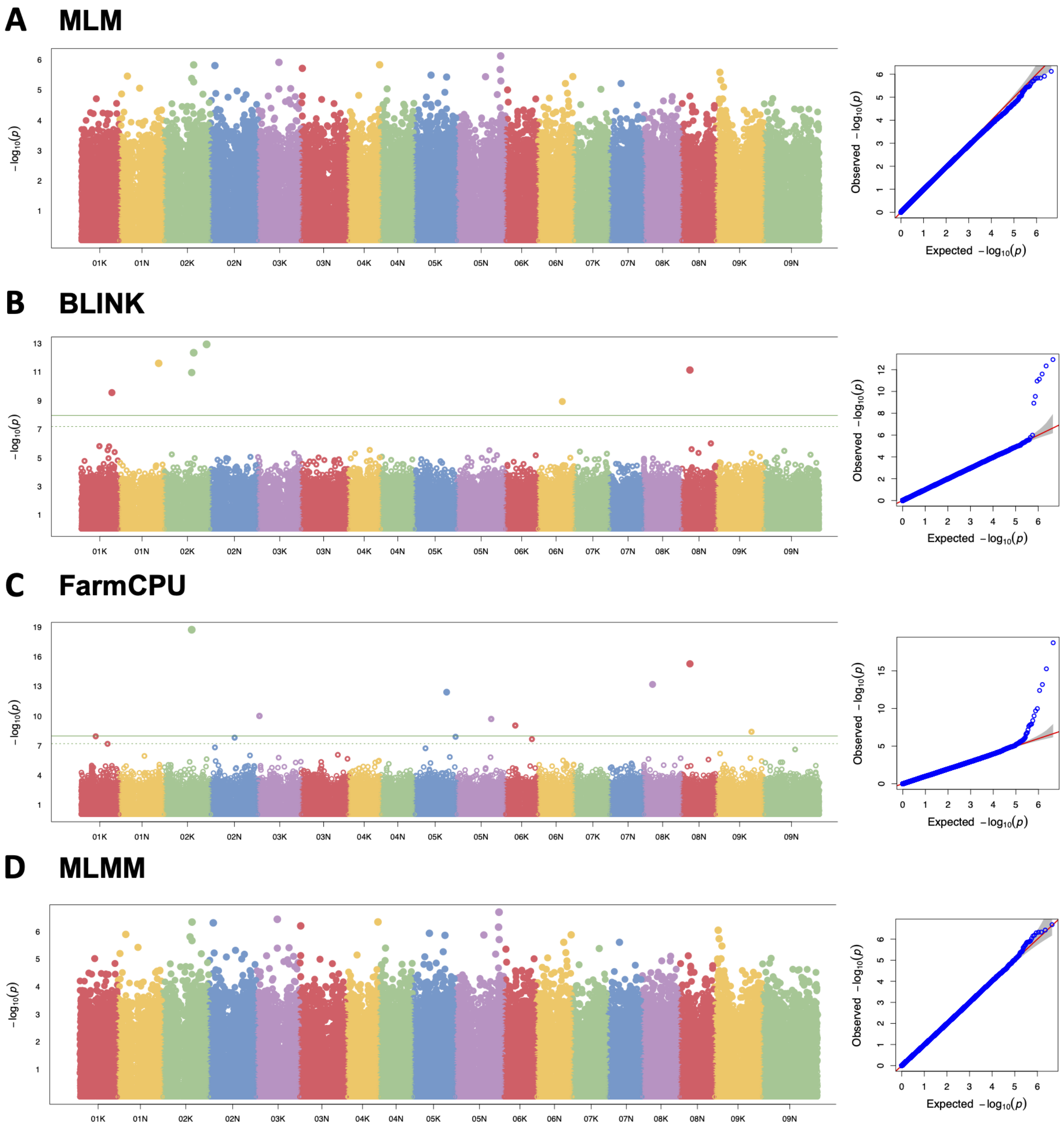

Figure 2. Switchgrass GWAS Manhattan plots from one single locus (A; mixed linear model; MLM) and three multi-locus (BLINK, B; FarmCPU, C; and MLMM, D) using GAPIT. Solid line indicates significance at FDR < 0.2, dotted line indicates suggestive significance. Insets: Q-Q plots for each method.

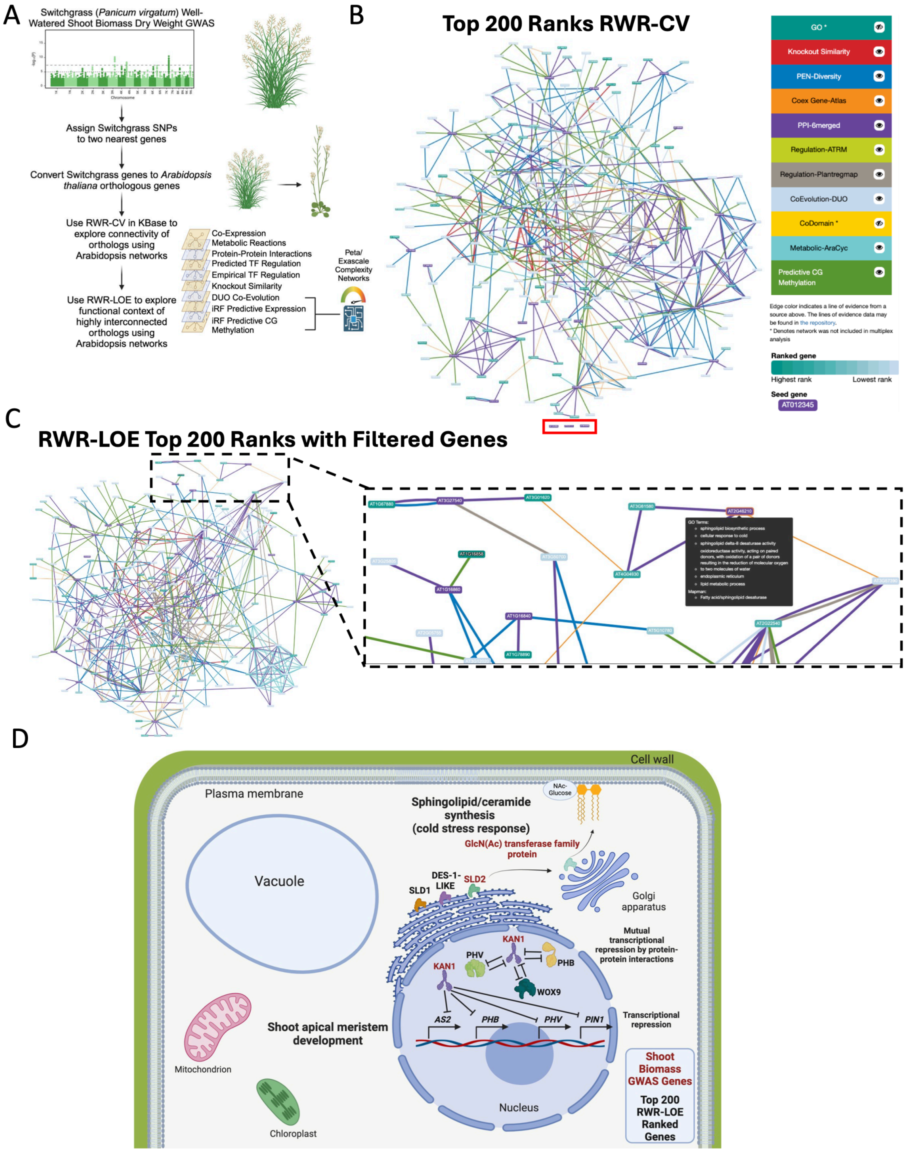

Figure 3. A. Workflow diagram. Switchgrass GWAS models identify significant single nucleotide polymorphism (SNP) associations with well-watered shoot biomass dry weight, and these SNPs are assigned to the two nearest Switchgrass genes. Switchgrass genes are converted to Arabidopsis orthologs, which are used as seeds in Arabidopsis multiplex networks. KBase apps were used to explore highly interconnected genes and identify functional context of the GWAS genes using a 9-layer multiplex, including 3 network layers (DUO Co-Evolution, iRF Predictive Expression, and iRF Predictive CG Methylation) derived from high-performance computing using models with petascale or exascale-level combinatorial complexity. Figure created using Biorender.com. **B.** Using KBase, RWR-CV identified three seed genes (inset, red box) that were unconnected from the top 200 ranked genes. Seed genes (Arabidopsis orthologs from GWAS results) are displayed as purple nodes, and teal to light blue nodes are color scaled based on RWR-LOE ranks. Edge colors indicate the line of evidence from which each gene-gene relationship was derived. **C.** Visualization of KBase RWR-LOE top 200 ranks output using orthologs of genes that were highly interconnected based on RWR-CV ("filtered genes"). Inset: Magnified view of network. When hovering over a gene, users can view Gene Ontology (GO), knockout phenotype and MAPMAN annotations for that gene. **D.** Framework for a conceptual model of Switchgrass well-watered shoot biomass with GWAS genes (red) and top 200 ranked genes by RWR-LOE (black). Genes from GWAS and RWR-LOE ranks implicated shoot apical meristem development, homeodomain transcription factors involved in transcriptional repression, and sphingolipid/ceramide synthesis. Figure made with BioRender.com.

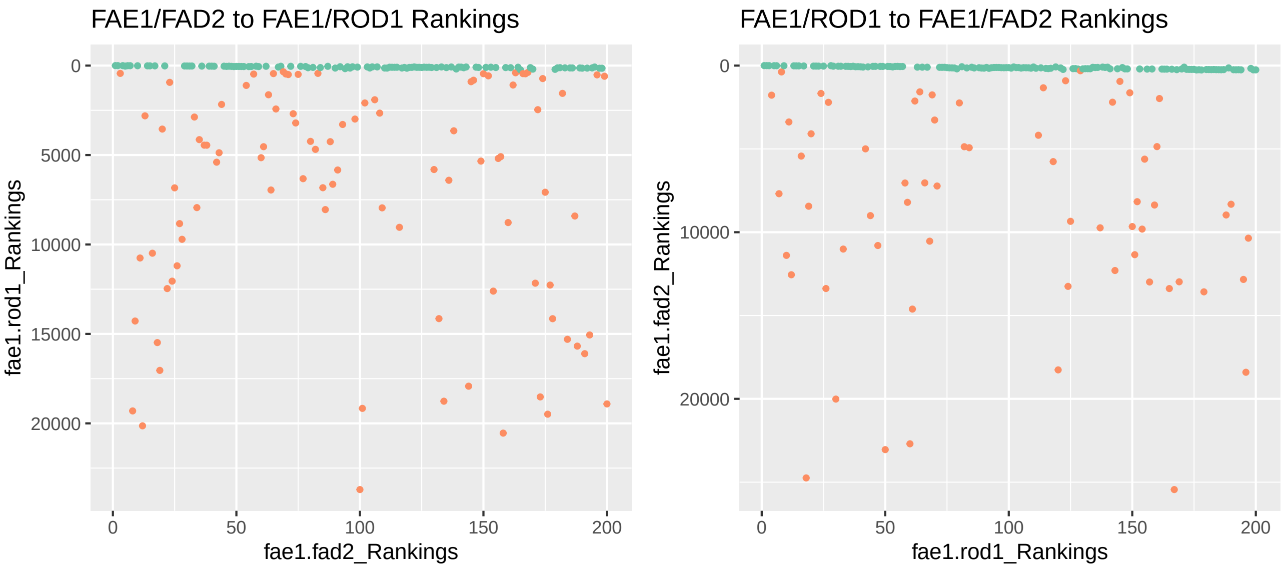

Figure 4. Illustrating RWR_LOE Rank Differential between pennycress dual knockout experiments FAE1/FAD2 and FAE1/ROD1. The top 200 ranked genes from RWR_LOE of FAE1/FAD2 (x-axis, left) and FAE1/ROD1 (x-axis, right) are shown plotted against their corresponding ranks (y-axis) from RWR_LOE of FAE1/ROD1 and FAE1/FAD2, respectively. Lower numbers mean better ranking. The top 200 genes for one knockout pair generally achieve a similar ranking in the other knockout pair (blue dots). However, within each experiment, there exist genes that rank far worse when compared to their corresponding rank in the other experiment (orange dots).

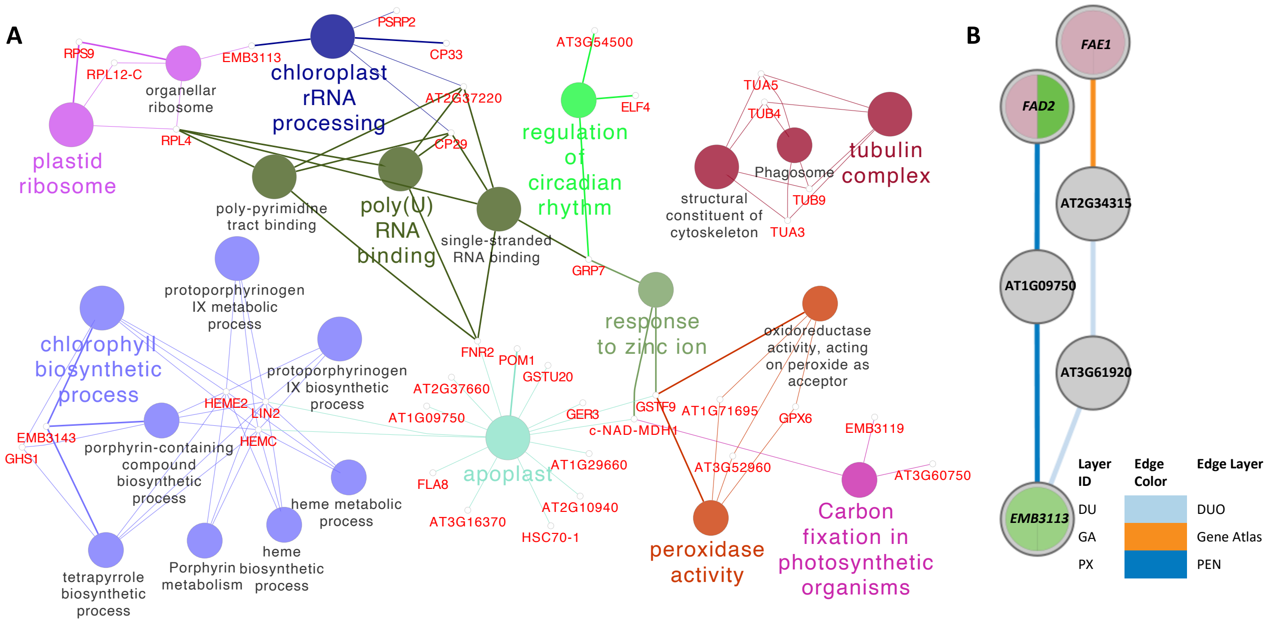

Figure 5. Top-ranked genes identify overlapping and distinct biological processes in pennycress from alternative knockout gene pairs. A. ClueGO enrichment of the FAE1/FAD2 specific sets of top ranked genes. A bipartite network where nodes are genes and enrichment terms, edges connecting genes to terms indicate enrichment. **B**. RWR Shortest paths between the FAE1/FAD2 gene set and EMB3113. The shortest path found from the FAD2 to EMB3113 uses edges from the predictive expression network while the path from FAE1 to EMB3113 uses edges from the gene atlas and duo network. In cases where edges exist on multiple layers, multiple labeled edges will exist between the two nodes.

Supplemental Tables

Table 1. Example network layer edge list with no edge weights to be used as input.

Gene A	Gene C
Gene A	Gene E
Gene B	Gene C
...	...
Gene E	Gene D

Table 2. Example network layer edge list with edge weights to be used as input.

Gene A	Gene C	0.8
Gene A	Gene E	0.2
Gene B	Gene C	0.15
...	...	...
Gene E	Gene D	0.99

Table 3. Example flist in which each row has a full path to an edge list and an associated layer name.

/path/to/layer1	layer1_name
/path/to/layer2	layer2_name
/path/to/layer3	layer3_name
...	...
/path/to/layerN	layerN_name

While relative paths within the flist can work with respect to the directory from which the program is run, it is highly recommended that users add full paths within their flists.

Table 4. RWR Gene Sets example file.

setA	gene_A
setA	gene_B
setA	gene_C

Most RWRtoolkit commands require the user to input a set of genes of interest, known as a gene set. A gene set is a tab-delimited file where the first column is the name of the gene set itself, the second column is the unique gene IDs, and the third (optional) column is a weight per gene which can be used to influence the random walk if desired. The genes in the gene set can be used as seeds for random walks, target genes in RWR_LOE and RWR_shortestpaths.

Table 5. Example RWR_netstats Basic Statistics Output.

network_name	number_of_nodes	number_of_edges	diameter
network_1	31	98	4.863
network_2	31	98	3.71
automated_textmining	31	160	1.792
coexpression	31	154	0.5813
combined_score	31	162	2.899
database_annotated	31	136	2.889
experimentally_determined_interaction	31	71	1.499
gene_fusion	31	5	1.809
homology	31	13	1.945
neighborhood_on_chromosome	31	87	1.848
phylogenetic_cooccurrence	31	20	1.88

In the above table, basic statistics are given for all layers of the supplied multiplex as well as the two additional networks supplied to the function.

Table 6. RWR_netstats pairwise_between_mpo_layer_jaccard.tsv.

	automated textmining	coexpression	combined score	database annotated	experimentally determined interaction	gene fusion	homology	neighborhood on chromosome	phylogenetic cooccurrence
automated textmining	1	0.9383	0.9877	0.8385	0.4348	0.03125	0.08125	0.5438	0.125
coexpression	0.9383	1	0.9506	0.7901	0.3975	0.02581	0.03727	0.5548	0.08075
combined score	0.9877	0.9506	1	0.8395	0.4383	0.03086	0.08025	0.537	0.1235
database annotated	0.8385	0.7901	0.8395	1	0.3019	0.03676	0.09559	0.4204	0.1223
experimentally determined interaction	0.4348	0.3975	0.4383	0.3019	1	0.0411	0.1507	0.4107	0.1974
gene fusion	0.03125	0.02581	0.03086	0.03676	0.0411	1	0	0.05747	0.08696
homology	0.08125	0.03727	0.08025	0.09559	0.1507	0	1	0.02041	0.65
neighborhood on chromosome	0.5438	0.5548	0.537	0.4204	0.4107	0.05747	0.02041	1	0.09184
phylogenetic cooccurrence	0.125	0.08075	0.1235	0.1223	0.1974	0.08696	0.65	0.09184	1

The above table illustrates Jaccard similarity between each layer in the multiplex.

Table 7. RWR_netstats pairwise_between_mpo_layer_overlap.tsv.

	automated textmining	coexpression	combined score	database annotated	experimentally determined interaction	gene fusion	homology	neighborhood on chromosome	phylogenetic cooccurrence
automated textmining	0.6474	0.6367	0.6395	0.6183	0.7178	0.8885	0.7788	0.7402	0.82
coexpression	0.2407	0.251	0.2386	0.1818	0.3292	0.4024	0.04705	0.3571	0.2417
combined score	0.9549	0.9542	0.9548	0.9549	0.9628	0.9976	0.9671	0.9643	0.9758
database annotated	0.7969	0.7908	0.7933	0.9449	0.616	1	0.8855	0.7216	0.7756
experimentally determined interaction	0.1685	0.1433	0.1726	0.1341	0.3938	0.2822	0.7429	0.1582	0.5923
gene fusion	0.01269	0.006692	0.01253	0.01493	0.003123	0.4061	0	0.02334	0.04966
homology	0.07738	0.03564	0.07643	0.09104	0.153	0	0.9524	0.01743	0.619
neighborhood on chromosome	0.2413	0.2454	0.2383	0.2025	0.2948	0.5791	0.04704	0.4437	0.2205
phylogenetic cooccurrence	0.1085	0.0689	0.1071	0.112	0.1882	0.2053	0.9573	0.07618	0.8677

The above table illustrates the overlap score between each layer in the multiplex.

Table 8. RWR_Netstats Multiplex Layers to Ref Net Jaccard example file output.

	jaccard
automated_textmining	0.5782
coexpression	0.5948
combined_score	0.
database_annotated	0.5826
experimentally_determined_interaction	0.5128
gene_fusion	0.3992
homology	0.3701
neighborhood_on_chromosome	0.5866
phylogenetic_cooccurrence	0.3884

This file contains statistics for the Jaccard score between the total number of edges within the intersection divided by the total number of edges within the union of both networks.

Table 9. RWR_Netstats Multiplex Layers to Ref Net overlap example file output.

	overlap
automated_textmining	0.5782
coexpression	0.5948
combined_score	0.5771
database_annotated	0.5826
experimentally_determined_interaction	0.5128
gene_fusion	0.3992
homology	0.3701
neighborhood_on_chromosome	0.5866
phylogenetic_cooccurrence	0.3884

This file sums the edge weights of all intersecting values and then divides those summed edge weights by the total number of edges within the network layer (note: this will be the same as the Jaccard method if all edges are weighted as 1 and the network layer contains all of the edges found within the reference network).

Table 10. RWR_Netstats net to net similarity example file output.

jaccard	overlap
0.4307	0.576

This function compares and calculates metrics for two provided network layers.

Table 11. RWR_Netstats calculated tau example file output.

	calculated_tau
automated_textmining	1.134
coexpression	1.166
combined_score	1.132
database_annotated	1.142
experimentally_determined_interaction	1.006
gene_fusion	0.7828
homology	0.7257
neighborhood_on_chromosome	1.15
phylogenetic_cooccurrence	0.7615

This function offers a more advanced method for users to calculate a tau parameter where the parameter is calculated as an "overlap" score with respect to a reference network for each layer.

Table 12. RWR_Netstats calculate exclusivity file output.

n_layers	pct_found
1	0.0185
2	0.9012
3	0.0802
4	0
5	0
6	0
7	0
8	0
9	0

The table illustrates the overall percentage of edges in the multiplex that exist in N layers. In the above output example, 2% of edges exist in only 1 layer, 90% of edges in the multiplex exist in 2 layers, and 8% of edges exist in 3 layers, with no other edges existing in more than 3 layers.

Table 13. RWR_LOE output example file output.

NodeNames	Score	rank	num_in_network	num_seeds	networks	modname	seed_geneset
H6PD	0.0007084	1	12	12	automated_t...nce	default	setA1
GPI	0.0007036	2	12	12	automated_t...nce	default	setA1
PFKP	0.0005773	3	12	12	automated_t...nce	default	setA1
HK1	0.0005156	4	12	12	automated_t...nce	default	setA1
GFPT2	0.0004941	5	12	12	automated_t...nce	default	setA1
…	…	…	…	…	…	…	…
GNPNAT1	0.0001826	16	12	12	automated_t...nce	default	setA1
PGLS	0.0001515	17	12	12	automated_t...nce	default	setA1
KHK	3.312e-05	18	12	12	automated_t...nce	default	setA1
IDNK	1.559e-05	19	12	12	automated_t...nce	default	setA1

This file denotes node name, associated score from the random walker, rank based off of the associated score, number of seeds in the supplied gene set within the network, the total number of seeds supplied, the multiplex network name (i.e. the combined names of all layers within the network), the modname (alias for experiment name output), and the name of the seed gene set.

Table 14. RWR_CV Full Ranks example file output.

NodeNames	Score	rank	InValset	num in network	num seeds	num leftout	networks	fold	modname	geneset	seed	leftout	method
ENO1	0.001162	1	1	12	8	4	aut...nce	1	default	setA1	many	many	kfold
PFKP	0.0008004	2	0	12	8	4	aut...nce	1	default	setA1	many	many	kfold
GPI	0.0007388	3	0	12	8	4	aut...nce	1	default	setA1	many	many	kfold
...	...	...	...	...	...	...	...	...	...	...	...	...	...
GFPT1	6.626e-05	20	1	12	8	4	aut...nce	3	default	setA1	many	many	kfold
GFPT2	6.518e-05	21	0	12	8	4	aut...nce	3	default	setA1	many	many	kfold
KHK	4.215e-05	22	0	12	8	4	aut...nce	3	default	setA1	many	many	kfold
IDNK	1.977e-05	23	0	12	8	4	aut...nce	3	default	setA1	many	many	kfold

This file contains the output ranking for each RWR_CV fold, including the base statistics from RWR_LOE seen previously, but also contains InValset, denoting whether the ranked gene was in the target set, fold, number of seed genes, number left out genes, and the method for cross validation.

Table 15. RWR_CV Mean Ranks example file output.

NodeNames	meanrank	rerank	InValset	geneset	num_in_network
ENO1	1	1	1	setA1	12
TPI1	1	1	1	setA1	12
ENO3	3	3	1	setA1	12
GPI	3.67	4	0	setA1	12
...	...	...	...	...	...
HK3	10.67	16	0	setA1	12
G6PD	11	17	1	setA1	12
PGM1	11.67	18	0	setA1	12
HK2	14.67	19	0	setA1	12
...	...	...	...	...	...

This file contains the mean ranks for each gene across all folds of RWR_CV and reranks each gene with respect to those floating point mean ranks.

Table 16. RWR_CV Metrics example file output.

NodeNames	meanrank	rerank	InValset	geneset	num_in_network	TP	FP	cum_TP	cum_FP	FPR	PREC	REC
ENO1	1	1	1	setA1	12	1	0	1	0	0	1	0.083
TPI1	1	1	1	setA1	12	1	0	2	0	0	1	0.167
ENO3	3	3	1	setA1	12	1	0	3	0	0	1	0.25
GPI	3.67	4	0	setA1	12	0	1	3	1	0.053	0.75	0.25
...	...	...	...	...	...	...	...	...	...	...	...	...
PMM1	20	26	1	setA1	12	1	0	11	17	0.895	0.393	0.917
GCK	21	29	1	setA1	12	1	0	12	17	0.895	0.414	1
KHK	22	30	0	setA1	12	0	1	12	18	0.947	0.4	1
IDNK	23	31	0	setA1	12	0	1	12	19	1	0.387	1

This file contains the mean ranks denoted above, but additionally does contain metrics such as True Positive (TP), which recalls a gene within the target gene set, False Positive (FP), which recalls a gene not within the target gene set, Cumulative True Positive (cum_TP), which sums all TP as ranks increase, Cumulative False Positive (cum_FP), which sums all FP as ranks increase, False Positive Rate (FPR) which divides cum_FP by the sum of cum_FP and the total number of genes in the network not in the seed set, Precision (PREC) which is cum_TP divided by the sum of cum_TP and cum_FP, and Recall (REC), which is the cum_TP divided by the sum of cum_TP and the difference of cum_TP and num_in_network (False Negative).

Table 17. RWR_CV Summary example file output.

fold	value	measure	geneset
1	0.25	P@NumLeftOut	setA1
2	0	P@NumLeftOut	setA1
3	0.5	P@NumLeftOut	setA1
1	0.398	AvgPrec	setA1
2	0.162	AvgPrec	setA1
3	0.514	AvgPrec	setA1
1	0.249	AUPRC	setA1
2	0.137	AUPRC	setA1
3	0.364	AUPRC	setA1
1	0.174	ExpectedAUPRC	setA1
2	0.174	ExpectedAUPRC	setA1
3	0.174	ExpectedAUPRC	setA1
1	0.5	AUROC	setA1
2	0.402	AUROC	setA1
3	0.609	AUROC	setA1
meanrank	0.534	AvgPrec	setA1
meanrank	0.475	AUPRC	setA1
meanrank	0.486	AUROC	setA1

This file contains overall summary statistics for each individual fold as well as mean rank including Average Precision, Area Under the Precision Recall Curve (AUPRC), Expected AUPRC, Area under the Receiver Operating Curve (AUROC).

Table 18. RWR_shortestpaths example file output.

from	to	weight	type	weightnorm	pathname	pathlength	pathelements
G6PD	HK2	0.8889	database_annotated	0.006917	G6PD_HK2	2	G6PD->HK2
G6PD	HK2	0.956	combined_score	0.006181	G6PD_HK2	2	G6PD->HK2
…	…	…	…	…	…	…	…
GFPT1	HK3	1	database_annotated	0.007781	GFPT1_HK3	2	GFPT1->HK3
GFPT1	HK3	0.9259	combined_score	0.005986	GFPT1_HK3	2	GFPT1->HK3
…	…	…	…	…	…	…	…
MPI	PMM2	0.1182	coexpression	0.003058	PKLR_PMM2	4	PKLR->GPI->MPI->PMM2
MPI	PMM2	0.8968	automated_textmining	0.008657	PKLR_PMM2	4	PKLR->GPI->MPI->PMM2

This file extracts all connections within a path between nodes in the source gene set and target gene set. Edges for each path are saved with the layers in which those edges exist as well as each edge's original edge weight, normalized edge weight, a unique path name id, total path length, and a list of all elements within the path.

Table 19. Outer Join of the top 200 ranked nodes from *fae1 fad2* and top 200 ranked nodes from *fae1 rod1* using RWR_LOE

Node Names	Score fae1 fad2	rank fae1 fad2	Score fae1 rod1	rank fae1 rod1
AT1G01120	0.0002963	1	0.0002962	1
AT3G01513	5.793e-05	2	5.793e-05	2
AT2G29980	3.815e-05	3		
AT4G34510	3.745e-05	4	3.697e-05	3
AT2G43710	3.052e-05	5	8.268e-06	28
AT3G01530	2.602e-05	6	2.605e-05	5
AT3G01510	2.552e-05	7	2.524e-05	6
AT1G09750	2.312e-05	8		
AT2G37220	2.302e-05	9		
AT3G10185	2.086e-05	10	2.086e-05	9
AT3G53460	1.773e-05	11		
AT2G43550	1.666e-05	12		
AT4G27130	1.631e-05	13		
AT1G19440	1.518e-05	14	1.675e-05	13
AT3G10180	1.404e-05	15	1.403e-05	14
AT4G38770	1.387e-05	16		
AT1G23310	1.314e-05	17	1.311e-05	15
AT1G56070	1.273e-05	18		
AT4G21860	1.264e-05	19		
AT1G49720	1.263e-05	20		
AT5G08430	1.261e-05	21	1.258e-05	17
AT5G17890	1.248e-05	22		
AT5G54590	1.226e-05	23		
AT2G17870	1.222e-05	24		
AT5G67320	1.205e-05	25		
AT3G06510	1.177e-05	26		
AT2G36530	1.174e-05	27		
AT1G53670	1.173e-05	28		
AT5G10060	1.142e-05	29	1.14e-05	21
AT1G25450	1.077e-05	30	1.075e-05	22
AT2G16280	1.042e-05	31	9.51e-06	25
AT3G14300	1.019e-05	32	1.019e-05	23
AT5G16230	9.231e-06	33		
AT3G02610	8.75e-06	34		
AT5G05580	7.951e-06	35		
AT2G15090	7.872e-06	36	7.866e-06	29
AT3G11170	7.778e-06	37		
AT3G11400	7.529e-06	38		
AT4G25010	7.468e-06	39	7.485e-06	31
AT4G25960	6.815e-06	40	6.796e-06	32
AT3G20660	6.427e-06	41	4.938e-06	37
AT3G02490	6.318e-06	42		
AT3G15870	6.245e-06	43		
AT5G55470	6.066e-06	44		
AT1G10050	5.936e-06	45	5.937e-06	34
AT2G47245	5.918e-06	46	3.934e-06	46
AT1G69870	5.692e-06	47	5.757e-06	35
AT1G64400	5.6e-06	48	3.944e-06	45
AT3G05970	5.371e-06	49	3.516e-06	55
AT4G11030	5.352e-06	50	3.722e-06	48
AT5G27600	5.35e-06	51	3.573e-06	49
AT2G04350	5.325e-06	52	3.56e-06	51
AT4G23850	5.308e-06	53	3.534e-06	54
AT2G31360	5.307e-06	54		
AT2G47240	5.302e-06	55	3.442e-06	57
AT1G77590	5.29e-06	56	3.55e-06	52
AT1G64900	5.242e-06	57		
AT5G60070	5.221e-06	58	5.22e-06	36
AT1G49430	5.104e-06	59	3.455e-06	56
AT2G37760	5.03e-06	60		
AT3G07070	4.914e-06	61		
AT4G34540	4.888e-06	62	4.85e-06	39
AT4G33600	4.882e-06	63		
AT4G19000	4.695e-06	64		
AT5G04630	4.579e-06	65		
AT3G01800	4.443e-06	66		
AT4G00360	4.429e-06	67	1.062e-06	81
AT1G68530	4.409e-06	68	4.905e-06	38
AT1G64930	4.4e-06	69		
AT3G10560	4.37e-06	70		
AT3G03470	4.344e-06	71		
AT4G36830	4.325e-06	72	4.333e-06	40
AT5G66570	4.22e-06	73		
AT2G45840	4.197e-06	74		
AT1G11600	4.177e-06	75		
AT4G34550	3.909e-06	76	4.106e-06	43
AT4G06599	3.796e-06	77		
AT4G34530	3.569e-06	78	3.54e-06	53
AT1G01600	3.557e-06	79	7.163e-07	119
AT3G18220	3.535e-06	80		
AT1G63710	3.524e-06	81	8.224e-07	102
AT3G61290	3.49e-06	82		
AT5G04660	3.472e-06	83		
AT5G58860	3.47e-06	84	7.792e-07	110
AT1G72120	3.329e-06	85		
AT4G15960	3.067e-06	86		
AT1G71160	3.056e-06	87	4.299e-06	41
AT3G15850	3.029e-06	88		
AT4G26400	3.02e-06	89		
AT2G45970	3.005e-06	90	5.726e-07	138
AT3G13682	2.699e-06	91		
AT4G01220	2.681e-06	92	2.716e-06	63
AT4G30950	2.588e-06	93		
AT2G27690	2.529e-06	94	4.509e-07	171
AT3G55360	2.37e-06	95	2.347e-06	65
AT1G02660	1.948e-06	96	5.592e-07	140
AT5G40750	1.939e-06	97	1.939e-06	67
AT2G13290	1.921e-06	98		
AT4G36480	1.81e-06	99	1.009e-06	85
AT2G00550	1.664e-06	100		
AT5G58250	1.661e-06	101		
AT3G23770	1.655e-06	102		
AT5G04530	1.603e-06	103	1.266e-06	74
AT3G61680	1.596e-06	104	5.881e-07	134
AT2G26250	1.573e-06	105	1.559e-06	72
AT5G08280	1.5e-06	106		
AT4G31060	1.419e-06	107	1.299e-06	73
AT1G20840	1.367e-06	108		
AT3G60750	1.35e-06	109		
AT4G13050	1.348e-06	110	5.778e-07	136
AT3G25110	1.314e-06	111	5.636e-07	139
AT3G56700	1.31e-06	112	8.921e-07	91
AT1G66550	1.309e-06	113	8.689e-07	95
AT3G60060	1.298e-06	114	8.621e-07	96
AT5G22500	1.286e-06	115	8.784e-07	94
AT3G02630	1.282e-06	116		
AT1G01710	1.276e-06	117	5.826e-07	135
AT4G00520	1.268e-06	118	8.196e-07	104
AT3G62590	1.259e-06	119	5.415e-07	146
AT1G35290	1.236e-06	120	8.215e-07	103
AT4G10030	1.231e-06	121	8.336e-07	100
AT4G34250	1.225e-06	122	1.218e-06	75
AT3G52570	1.224e-06	123	8.352e-07	99
AT5G23670	1.224e-06	124	8.525e-07	97
AT5G48370	1.224e-06	125	8.174e-07	106
AT3G11980	1.223e-06	126	9.253e-07	88
AT3G48780	1.215e-06	127	8.421e-07	98
AT1G06520	1.212e-06	128	8.815e-07	93
AT2G30720	1.209e-06	129	8.062e-07	107
AT1G01060	1.186e-06	130		
AT3G48790	1.18e-06	131	7.92e-07	108
AT3G02620	1.16e-06	132		
AT1G62640	1.154e-06	133	1.146e-06	76
AT5G19780	1.145e-06	134		
AT1G06515	1.135e-06	135	7.658e-07	113
AT4G18390	1.116e-06	136		
AT5G57260	1.109e-06	137	1.111e-06	77
AT5G60710	1.055e-06	138		
AT5G16240	1.053e-06	139	4.068e-07	189
AT5G51490	1.048e-06	140	1.048e-06	83
AT3G10280	1.034e-06	141	9.846e-07	86
AT5G01550	9.893e-07	142	6.831e-07	121
AT5G49070	9.858e-07	143	1.097e-06	78
AT3G52960	9.727e-07	144		
AT2G01820	9.482e-07	145		
AT2G01680	9.221e-07	146		
AT5G04480	9.141e-07	147	9.244e-07	89
AT5G46290	8.97e-07	148	8.175e-07	105
AT1G05850	8.805e-07	149		
AT1G14185	8.638e-07	150		
AT3G01500	8.566e-07	151	8.251e-07	101
AT3G27960	8.482e-07	152		
AT3G14630	8.472e-07	153	9.061e-07	90
AT5G19770	8.112e-07	154		
AT1G78640	7.804e-07	155	7.804e-07	109
AT3G16370	7.788e-07	156		
AT1G06120	7.73e-07	157		
AT5G20630	7.729e-07	158		
AT1G65880	7.677e-07	159	7.759e-07	111
AT2G33800	7.653e-07	160		
AT4G24510	7.463e-07	161	7.369e-07	117
AT1G06350	7.386e-07	162		
AT1G72970	7.385e-07	163		
AT1G04220	7.379e-07	164	8.871e-07	92
AT4G26740	7.219e-07	165		
AT5G51930	7.101e-07	166		
AT3G56060	7.029e-07	167		
AT1G12570	6.989e-07	168		
AT5G51950	6.972e-07	169	7.644e-07	115
AT3G12203	6.955e-07	170	3.959e-07	198
AT3G54500	6.94e-07	171		
AT5G61380	6.818e-07	172		
AT1G67260	6.801e-07	173		
AT2G45470	6.787e-07	174		
AT2G41900	6.676e-07	175		
AT2G30860	6.646e-07	176		
AT5G14920	6.638e-07	177		
AT3G14240	6.63e-07	178		
AT1G02205	6.578e-07	179		
AT1G22610	6.457e-07	180	6.364e-07	127
AT3G52160	6.409e-07	181	7.507e-07	116
AT2G44810	6.377e-07	182		
AT1G68380	6.368e-07	183	6.366e-07	126
AT2G10940	6.362e-07	184		
AT2G26640	6.345e-07	185	6.052e-07	131
AT2G19890	6.092e-07	186	6.042e-07	132
AT3G10410	6.081e-07	187		
AT5G62190	6.012e-07	188		
AT5G07210	6.006e-07	189	6.012e-07	133
AT4G33280	5.93e-07	190	5.431e-07	144
AT1G20020	5.902e-07	191		
AT4G25980	5.814e-07	192	5.542e-07	141
AT2G40080	5.78e-07	193		
AT5G48100	5.66e-07	194	6.064e-07	130
AT4G20380	5.65e-07	195	1.086e-06	79
AT3G10570	5.638e-07	196		
AT5G55370	5.405e-07	197	5.405e-07	147
AT1G28030	5.334e-07	198	5.334e-07	148
AT1G64950	5.282e-07	199		
AT3G05020			2.734e-05	4
AT3G06410			2.46e-05	7
AT5G04680			2.387e-05	8
AT4G30500			1.946e-05	10
AT2G36020			1.841e-05	11
AT5G11050			1.714e-05	12
AT4G11610			1.283e-05	16
AT5G49440			1.235e-05	18
AT4G39510			1.185e-05	19
AT4G32690			1.142e-05	20
AT1G47610			9.836e-06	24
AT4G26480			9.352e-06	26
AT5G66460			9.055e-06	27
AT3G07340			7.559e-06	30
AT1G52540			6.164e-06	33
AT4G22360			4.129e-06	42
AT4G26450			4.03e-06	44
AT4G29830			3.768e-06	47
AT1G16250			3.563e-06	50
AT1G15220			3.295e-06	58
AT4G32790			3.245e-06	59
AT3G10150			2.963e-06	60
AT1G22170			2.799e-06	61
AT4G26455			2.767e-06	62
AT5G50390			2.676e-06	64
AT1G18520			2.181e-06	66
AT5G15530			1.908e-06	68
AT3G60600			1.81e-06	69
AT5G42630			1.793e-06	70
AT3G53520			1.658e-06	71
AT4G35580			1.078e-06	80
AT4G32000			1.049e-06	82
AT4G27145			1.024e-06	84
AT3G12120			9.806e-07	87
AT3G62170			7.692e-07	112
AT3G05360			7.655e-07	114
AT4G39390			7.284e-07	118
AT2G29620			6.943e-07	120
AT5G38170			6.747e-07	122
AT5G22290			6.726e-07	123
AT2G24290			6.581e-07	124
AT1G12020			6.571e-07	125
AT5G50770			6.138e-07	128
AT1G05785			6.069e-07	129
AT5G57670			5.777e-07	137
AT1G73730			5.528e-07	142
AT1G22910			5.479e-07	143
AT5G02840			5.427e-07	145
AT2G39830			5.292e-07	149
AT1G32361			5.233e-07	150
AT4G14368			5.164e-07	151
AT4G37970			5.16e-07	152
AT2G34315			5.142e-07	153
AT2G35210			5.105e-07	154
AT1G76900			4.921e-07	155
AT4G00220			4.891e-07	156
AT4G28090			4.887e-07	157
AT3G01570			4.861e-07	158
AT5G27950			4.854e-07	159
AT3G23250			4.837e-07	160
AT1G72560			4.789e-07	161
AT5G08460			4.787e-07	162
AT2G23220			4.729e-07	163
AT5G36160			4.685e-07	164
AT4G26330			4.682e-07	165
AT5G50600			4.624e-07	166
AT3G43690			4.606e-07	167
AT4G37640			4.589e-07	168
AT1G22590			4.57e-07	169
AT2G45420			4.54e-07	170
AT1G28650			4.489e-07	172
AT2G44470			4.449e-07	173
AT3G04200			4.432e-07	174
AT3G54940			4.389e-07	175
AT1G17810			4.31e-07	176
AT1G48910			4.26e-07	177
AT5G40420			4.228e-07	178
AT3G51390			4.225e-07	179
AT1G62070			4.215e-07	180
AT3G25260			4.204e-07	181
AT5G38160			4.183e-07	182
AT1G78390			4.179e-07	183
AT5G38195			4.149e-07	184
AT5G07200			4.147e-07	185
AT3G58720			4.141e-07	186
AT5G51210			4.118e-07	187
AT5G04430			4.107e-07	188
AT3G07990			4.06e-07	190
AT5G59170			4.05e-07	191
AT1G62060			4.04e-07	192
AT3G27660			4.03e-07	193
AT4G36870			3.998e-07	194
AT4G14820			3.991e-07	195
AT2G26480			3.987e-07	196
AT5G53810			3.979e-07	197
AT5G08580			3.956e-07	199
