# Supplementary material for: RWRtoolkit: multi-omic network analysis using random walks on multiplex networks in any species
Source: Gigascience. 2025 Apr 24;14:giaf028. doi: 10.1093/gigascience/giaf028 (PMC12020474; doi:10.1093/gigascience/giaf028)
Supplement: giaf028_GIGA-D-24-00348_Revision_1 [file giaf028_giga-d-24-00348_revision_1.pdf]

GigaScience

RWRtoolkit: multi-omic network analysis using random walks on multiplex networks in any species

--Manuscript Draft--

**Manuscript Number:**	GIGA-D-24-00348R1	
**Full Title:**	RWRtoolkit: multi-omic network analysis using random walks on multiplex networks in any species	
**Article Type:**	Research	
**Funding Information:**	Biological and Environmental Research (DE-AC02-05CH11231)	Dr. Daniel Jacobson
	Biological and Environmental Research (DE-SC0021286)	Dr. Daniel Jacobson
	Center for Bioenergy Innovation	Dr. Daniel Jacobson
**Abstract:**	We introduce RWRtoolkit, a multiplex generation, exploration, and statistical package built for R and command line users. RWRtoolkit enables the efficient exploration of large and highly complex biological networks generated from custom experimental data and/or from publicly available datasets, and is species agnostic. A range of functions can be used to find topological distances between biological entities, determine relationships within sets of interest, search for topological context around sets of interest, and statistically evaluate the strength of relationships within and between sets. The command-line interface is designed for parallelization on high performance cluster systems, which enables high throughput analysis such as permutation testing. Several tools in the package have also been made available for use in reproducible workflows via the KBase web application.	
**Corresponding Author:**	Daniel Jacobson, Ph.D.<br>Oak Ridge National Laboratory<br>Oak Ridge, Tennessee UNITED STATES	
**Corresponding Author Secondary Information:**		
**Corresponding Author's Institution:**	Oak Ridge National Laboratory	
**Corresponding Author's Secondary Institution:**		
**First Author:**	Matthew Lane, M.S.	
**First Author Secondary Information:**		
**Order of Authors:**	Matthew Lane, M.S.	
	David Kainer	
	Kyle Sullivan	
	J. Izaak Miller	
	Mikaela Cashman	
	Mallory Morgan	
	Ashley Cliff	
	Jonathon Romero	
	Angelica Walker	
	D. Dakota Blair	
	Hari Chhetri	
	Yongqin Wang	

Powered by Editorial Manager® and ProduXion Manager® from Aries Systems Corporation

	Mirko Pavicic
	Anna Furches
	Jaclyn Noshay
	Meghan Drake
	AJ Ireland
	Ali Missaoui
	Yun Kang
	John C Sedbrook
	Paramvir Dehal
	Shane Canon
	Daniel Jacobson
**Order of Authors Secondary Information:**	
**Response to Reviewers:**	We appreciate the thoughtful comments provided by both reviewers. We have implemented their feedback which has considerably strengthened the present manuscript. Below are point-by-point responses to each reviewer comment:<br><br>Reviewer #1:<br><br>Kainer and Lane et al. present a software package, RWRToolkit, for generating and analyzing integrated biological networks based on the Random Walk with Restart (RWR) algorithm. The key deliverable of this work is R software that executes and expands upon an existing RWR package. More specifically, the introduced functionality focuses on identifying genes within the integrated network that are connected to user-inputted seed genes, nominating these candidates as functionally related. But the bulk of the manuscript is actually presenting the results of two genetic analyses in plants, which are also used as examples of using RWR.<br><br>Response: We greatly thank the reviewer for pointing this out. We acknowledge that the manuscript originally placed significant emphasis on the case studies. This was done to illustrate the practical applications and robustness of the software across diverse biological scenarios. To address this, we have moved the case studies to the supplemental and have instead focused primarily on descriptions of the software's unique functionalities and emphasized its user-friend design and integration with KBase. We believe this should make the purpose of the paper clearer and simplify the message overall.<br><br>The authors have confused their message by presenting novel data and results in a manuscript that is ostensibly about software. Furthermore, the software itself is, by the authors' own description, a "wrap(per)" for RandomWalkRestartMH - the algorithm has been published and is described and evaluated in other papers (that are cited). There is additional functionality, but by focusing so much on evaluating the method and on novel results, the manuscript opens up the door to questions about those specific analyses and data, which I did not find compelling, as well as the RWR method itself, which is also not compelling as presented. This manuscript ultimately reads more like it is an application of RWR inference on the authors' biological problem of interest, rather than an objective assessment of the functionality of the software, much less of RWR itself.<br><br>Response: The reviewer's point is well made and we have worked to improve the narrative of the manuscript accordingly. We have revised our manuscript to better distinguish between our case studies and the software itself. We have illustrated the updates made to the RandomWalkRestartMH package codebase (speed improvements that allow for much larger networks, fixed the tau parameter etc), our

extended feature implementation, and have left the interpretation of the biological narratives as case studies for readers to browse at their own leisure. The changes can be found in the Data Description, Discussion, and Methods section.

On the positive side, the software offers what seems to be convenient functionality to build integrated networks and facilitate analyses. The Github is well organized and documented, with an extensive test suite. The authors further make their implementation as well as an Arabidopsis integrated network available on a public data repository. The introduction is well-written and efficiently contextualizes the generation of integrated biological networks.

Response: We appreciate the Reviewer's positive opinion of the software's added capabilities in multiplex network building, our GitHub documentation and Arabidopsis multiplex networks, and the Introduction to the manuscript.

However, the manuscript does not provide a sufficient sense for what functionality is missing or underdeveloped in the RandomWalkRestartMH package. That is, what exact problem is solved by RWRToolkit - if someone wanted to do cross-validation using RandomWalkRestartMH for example, what would be involved without RWRToolkit? Based on the vignettes for the RandomWalkRestartMH package, it seems designed for the kind of analyses that the authors here do on the plant genes, is RWRToolkit really necessary to do that? Perhaps RWRToolkit makes it easier? The authors should make the advantages of using RWRToolkit vs. the underlying wrapped package much clearer. Perhaps this can all be addressed by the authors, but the other major problem I have with the manuscript is with the evaluation and applications.

Response: We thank the reviewers for noting that we did not make clear the advantages of using the RWRtoolkit functionality compared to RandomWalkRestartMH. We have updated the manuscript to illustrate why users would wish to use the RWRtoolkit compared to RandomWalkRestartMH, focusing on the software's usability with its file based input and command line interface options as well as KBase integration. Additionally, our updated RandomWalkRestartMH codebase drastically reduces the compute time required for generation, and we have fixed the Tau parameter to properly weigh the seeds across layers, and we have been sure to note these within the "RandomWalkRestartMH Updates" subsections within the Data Description, Results, Discussion, and Methods section.

The evaluation of the RWR algorithm presented in figure 3 is underdeveloped and unconvincing. No context/comparison is provided for how the analyzed multiplex network performs relative to other approaches, other than a shuffled null. One reason I find this unconvincing is that the groups of genes to be tested were selected by the authors because they were, according to the methods section (Network Validation), already known to be highly connected. I have to assume that "highly connected" refers to information that is known to be captured in the networks used. So it seems they have chosen examples that are known, ahead of time, to be likely to "work", but I also expect they will "work" with just about any network analysis method.

Response: We apologize for our lack of clarity here. It was, indeed, incorrect of us to describe the selected groups of genes as "highly connected" as this would imply that we selected genes that were already known to be connected in the multiplex. In reality, the gene sets were selected solely based on shared MAPMAN annotations, meaning the genes in a set are known to have similar functionality/role according to curated evidence, but the multiplex itself was never used for selection. We also recognise that if a network containing connections derived from MAPMAN were included in the multiplex, using gene sets from that database for validation would create a circular argument. We have updated the text to explain the gene set selection more carefully.

If they want to turn this into an algorithm evaluation paper (which I rather advise against), the authors must contrast other algorithms and test the performance of the Arabidopsis multiplex network to a simple "monoplex" network generated from the

same input layers, and reconsider what gene sets they use for testing.

Response: We agree with the reviewer’s advice here. We do not intend this paper to be an algorithm evaluation.

Additional comparisons of the performance of the multiplex layer relative to each individual layer would also be valuable, given that the entire work is predicated on the superior ability of multi-modal, multiplex networks to predict functionally related genes. But I have to emphasize that embarking on an evaluation of RWR should have been out of scope for a simple "wrapper": the authors are the ones who have opened the door to questioning the efficacy of the method by presenting this evaluation. The only reason I can think they felt the need to include an evaluation was because they then go on to apply RWR to the same network for discovery - that is, perhaps their intention is to evaluate the network, not the algorithm. But since the evaluation is unconvincing, it doesn't do that too well either.
Response: Thank you for the reviewer’s thoughtful suggestion. We agree that while additional comparisons of multiplex network performance relative to individual layers could be valuable, the primary focus of this manuscript is on extending and updating the RandomWalkRestartMH package. As such, conducting comprehensive evaluations of all layers to validate the superiority of multiplex networks is beyond the scope of this work.

The application to the genetics studies doesn't add all that much beyond "the software can be run." Despite this, discussion of the genetic studies takes up a lot of this manuscript - even without the RWR results. This means that I have to evaluate these genetics studies, and to the extent I have done so, they are unimpressive and I am not sure they would stand alone. This starts with the weak results from the GWAS, with four methods applied using a very lenient FDR of 0.2, two of which reporting no convincing associations. The remaining two methods identify 22 SNPs, with only one SNP being shared. I have no sense that this isn't just a false discovery. Despite this, the authors implicate a gene and use its ortholog a seed for the RWR_LOE method to find connected genes in the multiplex Arabidopsis network. All of this can be criticized and questioned - and perhaps choosing a random gene would have been just as interesting as what happens next. The authors use the candidate connected genes to suggest a functional connection to shoot biomass development. This line of speculation might be acceptable in the context of a study generating gene candidates involved in shoot biomass, but it is not a sufficient demonstration of the method's ability to retrieve validated functionally related genes - as the finding themselves are not validated. Further it is not clear that the same candidates would not have been readily identified by examining the top ranked partners of individual networks (i.e., coexpression) or a simplistic/monoplex network, or even just doing GO enrichment. The same sentiment applies to the other analysis of the knockouts, both as an evaluation of the RWR method, and in terms of being a meaningful discovery, or even that compelling of an exploratory analysis. But again, this really seems beside the point for describing the software.’

Response: We acknowledge the reviewer’s concern about the extent and emphasis placed on the genetic studies. The primary purpose of these studies was to demonstrate the practical utility and application of the software rather than present standalone biological discoveries. We have revised the manuscript to focus on illustrating RWRtoolkit’s functionalities and software updates to RandomWalkRestartMH and shifted the case studies to supplementary material.

Minor comments:

* Multiple figures suffer from poor legibility, such as the legends and axis labels in Fig. 3 and the text of Figs. 4 and 5.
Response: We have updated our figures to have a greater font size with respect to the legends and axes. Particularly, we have additionally updated our illustrations to primarily be pdfs, to ensure the vector graphics of the fonts translate to the document.

* The section "Multiplex Generation" would benefit from a concise overview of how

the Multiplex network is generated in the first place in the context of RandomWalkRestartMH, even if this is just a reference to Fig.1. For example, the methods state that "the multiplex constructed with a delta value of 0.5" with no context provided for what this parameter means.
Response: We thank the reviewer for catching our assumptions. We have since included a description of the parameter within the construction section. We have included a concise description of how the multiplex networks were constructed by the RandomWalkRestartMH and how they are now constructed with respect to our updated codebase within the data description RandomWalkRestartMH Multiplex Generation section.

* Fig. 5A seems unnecessary, or at least underdeveloped. There is no need to add text of the status of each set within the Venn diagram; consider instead basic information such as the count of genes in each set. It's also very difficult to read and make sense of the hairballs.
Response: We thank the reviewer for commenting on the illustration. As we thoroughly discuss the processing of the data in the text, we have instead opted to illustrate the major findings with respect to the GO enrichment of the FAE1/FAD2 specific top ranked genes.

* Part of the results (including the main reference to Fig. 5) are in the Discussion, rather than the Analyses section.
Response: We deeply thank the reviewer for catching this mistake. We have since ensured that our results are properly within the results sections of our manuscript.

* The authors claim to "show" that the software is "easy-to-use" (end of the Discussion). I do not see where they have evaluated this, which would require comparing user experience to some alternative, and I don't expect them to do that - I mean, I agree it doesn't seem particularly difficult to use, but it would be more appropriate to say it was designed with ease-of-use in mind. If they mean "easier than the underlying wrapped package", this needs to be described better as referred to under my major points.

Response: We thank the reviewer for nothing that we have not “shown” that the software is “easy-to-use”. We have made it a point to note in our manuscript that we have instead designed the software with “ease of use” in mind.

Reviewer #2:

The paper introduces a species agnostic random walk with restart toolkit built for R and command line users. The tool enables constructions of multiplex networks from any set of data layers and enables the discovery of gene-to-gene relationships. The tool offers a collection of functions for network analysis. Overall, the tool is a significant contribution to network analysis.
Response: We thank the reviewer for their positive feedback and for acknowledging the significance of RWRtoolkit as a contribution to network analysis. We are pleased to hear that you find the toolkit’s features and its potential for multiplex network analysis valuable.

Major Comments

The manuscript's background section should provide a more comprehensive overview of the rationale behind the development of RWRtoolkit. It should clearly outline the existing RWR implementation tools, identify the gaps in these tools, and explain how RWRtoolkit addresses these limitations or offers a new approach.

Response: We very much appreciate this suggestion to expand the background section to better contextualize the development of RWRtoolkit. As the RWRtoolkit fixes and extends the RandomWalkRestartMH package, we have primarily focused on comparisons with the RandomWalkRestartMH package itself.

	To demonstrate the effectiveness of RWRtoolkit, the authors could evaluate the ranking performance against other established random walk with restart algorithms that can handle heterogeneous multiplex networks. Additionally, a detailed explanation of the scoring approach implemented in RWRtoolkit is necessary to justify its choice and potential advantages.<br>Response: Thank you for your thoughtful suggestion. We would like to clarify that the primary goal of this manuscript is to update and extend the original RandomWalkRestartMH package, focusing on enhancing its functionality and usability. While comprehensive algorithm evaluation is beyond the scope of this work, we have included an evaluation of our updated methods with respect to the RandomWalkRestartMH methods. Additionally, we have included a detailed description of the evaluation and validation metric calculations within the supplemental data section. This ensures that users have the necessary context to assess the performance of the software in relevant biological scenarios. We hope this approach strikes an appropriate balance between extending the original package and providing meaningful validation insights.<br><br>The authors have indicated in the section "network layer and multiplex statistics" that the tau parameter affects the probability of the walker visiting each specific layer. To address potential bias issues in the network exploration, it would be beneficial to provide an exploration of the parameter space and indicate how it informs the stability of the RWR output scores under variations of the various algorithm parameters.<br>Response: We agree that an exploration of the tau parameter space is essential for understanding its impact on network exploration. In the revised manuscript, we have included an in-depth analysis of the parameter space with respect to our updated version as well as the prior implementation of RandomWalkRestartMH, focusing on the tau parameter's effect on the walker's behavior across layers. This includes visualizations and metrics to illustrate how variations in tau influence RWR output scores for the updated and previous implementations.
**Additional Information:**	
**Question**	**Response**
Are you submitting this manuscript to a special series or article collection?	No
**Experimental design and statistics**<br><br>Full details of the experimental design and statistical methods used should be given in the Methods section, as detailed in our Minimum Standards Reporting Checklist. Information essential to interpreting the data presented should be made available in the figure legends.<br><br>Have you included all the information requested in your manuscript?	Yes
**Resources**<br><br>A description of all resources used, including antibodies, cell lines, animals	Yes

Powered by Editorial Manager® and ProduXion Manager® from Aries Systems Corporation

and software tools, with enough information to allow them to be uniquely identified, should be included in the Methods section. Authors are strongly encouraged to cite Research Resource Identifiers (RRIDs) for antibodies, model organisms and tools, where possible.<br><br>Have you included the information requested as detailed in our Minimum Standards Reporting Checklist?	
**Availability of data and materials**<br><br>All datasets and code on which the conclusions of the paper rely must be either included in your submission or deposited in publicly available repositories (where available and ethically appropriate), referencing such data using a unique identifier in the references and in the "Availability of Data and Materials" section of your manuscript.<br><br>Have you have met the above requirement as detailed in our Minimum Standards Reporting Checklist?	Yes

Powered by Editorial Manager® and ProduXion Manager® from Aries Systems Corporation

maintext
Click here to access/download;LaTeX - Main Document (TeX file);main.tex

```
This is pdfTeX, Version 3.141592653-2.6-1.40.26 (TeX Live 2024)
(preloaded format=pdflatex 2024.8.2)  2 MAR 2025 20:34
entering extended mode
 restricted \write18 enabled.
 %&-line parsing enabled.
**main.tex
(./main.tex
LaTeX2e <2024-06-01> patch level 2
L3 programming layer <2024-05-27>
(./oup-contemporary.cls
Document Class: oup-contemporary 2023/06/12, v1.2
(c:/texlive/2024/texmf-dist/tex/latex/base/article.cls
Document Class: article 2024/02/08 v1.4n Standard LaTeX document class
(c:/texlive/2024/texmf-dist/tex/latex/base/size10.clo
File: size10.clo 2024/02/08 v1.4n Standard LaTeX file (size option)
)
\c@part=\count194
\c@section=\count195
\c@subsection=\count196
\c@subsubsection=\count197
\c@paragraph=\count198
\c@subparagraph=\count199
\c@figure=\count266
\c@table=\count267
\abovecaptionskip=\skip49
\belowcaptionskip=\skip50
\bibindent=\dimen141
) (c:/texlive/2024/texmf-dist/tex/latex/base/inputenc.sty
Package: inputenc 2024/02/08 v1.3d Input encoding file
\inpenc@prehook=\toks17
\inpenc@posthook=\toks18
) (c:/texlive/2024/texmf-dist/tex/latex/base/fontenc.sty
Package: fontenc 2021/04/29 v2.0v Standard LaTeX package
) (c:/texlive/2024/texmf-dist/tex/generic/iftex/ifpdf.sty
Package: ifpdf 2019/10/25 v3.4 ifpdf legacy package. Use iftex instead.
(c:/texlive/2024/texmf-dist/tex/generic/iftex/iftex.sty
Package: iftex 2022/02/03 v1.0f TeX engine tests
)) (c:/texlive/2024/texmf-dist/tex/latex/microtype/microtype.sty
Package: microtype 2024/03/29 v3.1b Micro-typographical refinements (RS)
(c:/texlive/2024/texmf-dist/tex/latex/graphics/keyval.sty
Package: keyval 2022/05/29 v1.15 key=value parser (DPC)
\KV@toks@=\toks19
) (c:/texlive/2024/texmf-dist/tex/latex/etoolbox/etoolbox.sty
Package: etoolbox 2020/10/05 v2.5k e-TeX tools for LaTeX (JAW)
\etb@tempcnta=\count268
)
\MT@toks=\toks20
\MT@tempbox=\box52
\MT@count=\count269
LaTeX Info: Redefining \noprotrusionifhmode on input line 1061.
LaTeX Info: Redefining \leftprotrusion on input line 1062.
\MT@prot@toks=\toks21
LaTeX Info: Redefining \rightprotrusion on input line 1081.
LaTeX Info: Redefining \textls on input line 1392.
```

```
\MT@outer@kern=\dimen142
LaTeX Info: Redefining \textmicrotypecontext on input line 2013.
\MT@listname@count=\count270
(c:/texlive/2024/texmf-dist/tex/latex/microtype/microtype-pdftex.def
File: microtype-pdftex.def 2024/03/29 v3.1b Definitions specific to
pdftex (RS)

LaTeX Info: Redefining \lsstyle on input line 902.
LaTeX Info: Redefining \lslig on input line 902.
\MT@outer@space=\skip51
)
Package microtype Info: Loading configuration file microtype.cfg.
(c:/texlive/2024/texmf-dist/tex/latex/microtype/microtype.cfg
File: microtype.cfg 2024/03/29 v3.1b microtype main configuration file
(RS)
)) (c:/texlive/2024/texmf-dist/tex/latex/euler/euler.sty
Package: euler 1995/03/05 v2.5
Package: `euler' v2.5 <1995/03/05> (FJ and FMi)
LaTeX Font Info:    Redeclaring symbol font `letters' on input line 35.
LaTeX Font Info:    Encoding `OML' has changed to `U' for symbol font
(Font)              `letters' in the math version `normal' on input line
35.
LaTeX Font Info:    Overwriting symbol font `letters' in version `normal'
(Font)                  OML/cmm/m/it --> U/eur/m/n on input line 35.
LaTeX Font Info:    Encoding `OML' has changed to `U' for symbol font
(Font)              `letters' in the math version `bold' on input line
35.
LaTeX Font Info:    Overwriting symbol font `letters' in version `bold'
(Font)                  OML/cmm/b/it --> U/eur/m/n on input line 35.
LaTeX Font Info:    Overwriting symbol font `letters' in version `bold'
(Font)                  U/eur/m/n --> U/eur/b/n on input line 36.
LaTeX Font Info:    Redeclaring math symbol \Gamma on input line 47.
LaTeX Font Info:    Redeclaring math symbol \Delta on input line 48.
LaTeX Font Info:    Redeclaring math symbol \Theta on input line 49.
LaTeX Font Info:    Redeclaring math symbol \Lambda on input line 50.
LaTeX Font Info:    Redeclaring math symbol \Xi on input line 51.
LaTeX Font Info:    Redeclaring math symbol \Pi on input line 52.
LaTeX Font Info:    Redeclaring math symbol \Sigma on input line 53.
LaTeX Font Info:    Redeclaring math symbol \Upsilon on input line 54.
LaTeX Font Info:    Redeclaring math symbol \Phi on input line 55.
LaTeX Font Info:    Redeclaring math symbol \Psi on input line 56.
LaTeX Font Info:    Redeclaring math symbol \Omega on input line 57.
\symEulerFraktur=\mathgroup4
LaTeX Font Info:    Overwriting symbol font `EulerFraktur' in version
`bold'
(Font)                  U/euf/m/n --> U/euf/b/n on input line 63.
LaTeX Info: Redefining \oldstylenums on input line 85.
\symEulerScript=\mathgroup5
LaTeX Font Info:    Overwriting symbol font `EulerScript' in version
`bold'
(Font)                  U/eus/m/n --> U/eus/b/n on input line 93.
LaTeX Font Info:    Redeclaring math symbol \aleph on input line 97.
LaTeX Font Info:    Redeclaring math symbol \Re on input line 98.
LaTeX Font Info:    Redeclaring math symbol \Im on input line 99.
```

```
LaTeX Font Info:    Redeclaring math delimiter \vert on input line 101.
LaTeX Font Info:    Redeclaring math delimiter \backslash on input line
103.
LaTeX Font Info:    Redeclaring math symbol \neg on input line 106.
LaTeX Font Info:    Redeclaring math symbol \wedge on input line 108.
LaTeX Font Info:    Redeclaring math symbol \vee on input line 110.
LaTeX Font Info:    Redeclaring math symbol \setminus on input line 112.
LaTeX Font Info:    Redeclaring math symbol \sim on input line 113.
LaTeX Font Info:    Redeclaring math symbol \mid on input line 114.
LaTeX Font Info:    Redeclaring math delimiter \arrowvert on input line
116.
LaTeX Font Info:    Redeclaring math symbol \mathsection on input line
117.
\symEulerExtension=\mathgroup6
LaTeX Font Info:    Redeclaring math symbol \coprod on input line 125.
LaTeX Font Info:    Redeclaring math symbol \prod on input line 125.
LaTeX Font Info:    Redeclaring math symbol \sum on input line 125.
LaTeX Font Info:    Redeclaring math symbol \intop on input line 130.
LaTeX Font Info:    Redeclaring math symbol \ointop on input line 131.
LaTeX Font Info:    Redeclaring math symbol \braceld on input line 132.
LaTeX Font Info:    Redeclaring math symbol \bracerd on input line 133.
LaTeX Font Info:    Redeclaring math symbol \bracelu on input line 134.
LaTeX Font Info:    Redeclaring math symbol \braceru on input line 135.
LaTeX Font Info:    Redeclaring math symbol \infty on input line 136.
LaTeX Font Info:    Redeclaring math symbol \nearrow on input line 153.
LaTeX Font Info:    Redeclaring math symbol \searrow on input line 154.
LaTeX Font Info:    Redeclaring math symbol \nwarrow on input line 155.
LaTeX Font Info:    Redeclaring math symbol \swarrow on input line 156.
LaTeX Font Info:    Redeclaring math symbol \Leftrightarrow on input line
157.
LaTeX Font Info:    Redeclaring math symbol \Leftarrow on input line 158.
LaTeX Font Info:    Redeclaring math symbol \Rightarrow on input line
159.
LaTeX Font Info:    Redeclaring math symbol \leftrightarrow on input line
160.
LaTeX Font Info:    Redeclaring math symbol \leftarrow on input line 161.
LaTeX Font Info:    Redeclaring math symbol \rightarrow on input line
163.
LaTeX Font Info:    Redeclaring math delimiter \uparrow on input line
166.
LaTeX Font Info:    Redeclaring math delimiter \downarrow on input line
168.
LaTeX Font Info:    Redeclaring math delimiter \updownarrow on input line
170.
LaTeX Font Info:    Redeclaring math delimiter \Uparrow on input line
172.
LaTeX Font Info:    Redeclaring math delimiter \Downarrow on input line
174.
LaTeX Font Info:    Redeclaring math delimiter \Updownarrow on input line
176.
LaTeX Font Info:    Redeclaring math symbol \leftharpoonup on input line
177.
LaTeX Font Info:    Redeclaring math symbol \leftharpoondown on input
line 178.
```

```
LaTeX Font Info:    Redeclaring math symbol \rightharpoonup on input line
179.
LaTeX Font Info:    Redeclaring math symbol \rightharpoondown on input
line 180
.
LaTeX Font Info:    Redeclaring math delimiter \lbrace on input line 182.
LaTeX Font Info:    Redeclaring math delimiter \rbrace on input line 184.
\symcmmigroup=\mathgroup7
LaTeX Font Info:    Overwriting symbol font `cmmigroup' in version `bold'
(Font)                  OML/cmm/m/it --> OML/cmm/b/it on input line 200.
LaTeX Font Info:    Redeclaring math accent \vec on input line 201.
LaTeX Font Info:    Redeclaring math symbol \triangleleft on input line
202.
LaTeX Font Info:    Redeclaring math symbol \triangleright on input line
203.
LaTeX Font Info:    Redeclaring math symbol \star on input line 204.
LaTeX Font Info:    Redeclaring math symbol \lhook on input line 205.
LaTeX Font Info:    Redeclaring math symbol \rhook on input line 206.
LaTeX Font Info:    Redeclaring math symbol \flat on input line 207.
LaTeX Font Info:    Redeclaring math symbol \natural on input line 208.
LaTeX Font Info:    Redeclaring math symbol \sharp on input line 209.
LaTeX Font Info:    Redeclaring math symbol \smile on input line 210.
LaTeX Font Info:    Redeclaring math symbol \frown on input line 211.
LaTeX Font Info:    Redeclaring math accent \grave on input line 245.
LaTeX Font Info:    Redeclaring math accent \acute on input line 246.
LaTeX Font Info:    Redeclaring math accent \tilde on input line 247.
LaTeX Font Info:    Redeclaring math accent \ddot on input line 248.
LaTeX Font Info:    Redeclaring math accent \check on input line 249.
LaTeX Font Info:    Redeclaring math accent \breve on input line 250.
LaTeX Font Info:    Redeclaring math accent \bar on input line 251.
LaTeX Font Info:    Redeclaring math accent \dot on input line 252.
LaTeX Font Info:    Redeclaring math accent \hat on input line 254.
) (c:/texlive/2024/texmf-dist/tex/latex/merriweather/merriweather.sty
Package: merriweather 2022/09/20 (Bob Tennent) Supports
Merriweather(Sans) font
s for all LaTeX engines.
(c:/texlive/2024/texmf-dist/tex/generic/iftex/ifxetex.sty
Package: ifxetex 2019/10/25 v0.7 ifxetex legacy package. Use iftex
instead.
) (c:/texlive/2024/texmf-dist/tex/generic/iftex/ifluatex.sty
Package: ifluatex 2019/10/25 v1.5 ifluatex legacy package. Use iftex
instead.
) (c:/texlive/2024/texmf-dist/tex/latex/base/textcomp.sty
Package: textcomp 2024/04/24 v2.1b Standard LaTeX package
) (c:/texlive/2024/texmf-dist/tex/latex/xkeyval/xkeyval.sty
Package: xkeyval 2022/06/16 v2.9 package option processing (HA)
(c:/texlive/2024/texmf-dist/tex/generic/xkeyval/xkeyval.tex
(c:/texlive/2024/te
xmf-dist/tex/generic/xkeyval/xkvutils.tex
\XKV@toks=\toks22
\XKV@tempa@toks=\toks23
)
\XKV@depth=\count271
```

```
File: xkeyval.tex 2014/12/03 v2.7a key=value parser (HA)
)) (c:/texlive/2024/texmf-dist/tex/latex/base/fontenc.sty
Package: fontenc 2021/04/29 v2.0v Standard LaTeX package
) (c:/texlive/2024/texmf-dist/tex/latex/fontaxes/fontaxes.sty
Package: fontaxes 2020/07/21 v1.0e Font selection axes
LaTeX Info: Redefining \upshape on input line 29.
LaTeX Info: Redefining \itshape on input line 31.
LaTeX Info: Redefining \slshape on input line 33.
LaTeX Info: Redefining \swshape on input line 35.
LaTeX Info: Redefining \scshape on input line 37.
LaTeX Info: Redefining \sscshape on input line 39.
LaTeX Info: Redefining \ulcshape on input line 41.
LaTeX Info: Redefining \textsw on input line 47.
LaTeX Info: Redefining \textssc on input line 48.
LaTeX Info: Redefining \textulc on input line 49.
)) (c:/texlive/2024/texmf-dist/tex/latex/mathastext/mathastext.sty
Package: mathastext 2024/07/27 v1.4b Use the text font in math mode (JFB)

Package mathastext Info: Starting the math mode configuration.
\mst@exists@muskip=\muskip17
\mst@forall@muskip=\muskip18
\mst@prime@muskip=\muskip19
\mst@do@nonletters=\toks24
\mst@undo@nonletters=\toks25
\mst@do@easynonletters=\toks26
\mst@undo@easynonletters=\toks27
\symmtoperatorfont=\mathgroup8
\symmtletterfont=\mathgroup9
(   mathastext:   ) ! and ?
(   mathastext:   ) punctuation: , . : ; and \colon
LaTeX Info: Redefining \relbar on input line 1201.
LaTeX Info: Redefining \rightarrowfill on input line 1202.
LaTeX Info: Redefining \leftarrowfill on input line 1205.
(   mathastext:   ) + and =
LaTeX Info: Redefining \Relbar on input line 1298.
(   mathastext:   ) adding = ; and + to \nfss@catcodes
(   mathastext:   ) parentheses ( ) [ ] and slash /
(   mathastext:   ) alldelims: < > \backslash \setminus | \vert \mid \{
\}
LaTeX Font Info:    Redeclaring math symbol \setminus on input line 1364.
LaTeX Info: Redefining \models on input line 1383.
(   mathastext:   ) \# \mathdollar \% \&
(   mathastext:   ) \imath and \jmath
LaTeX Font Info:    Overwriting math alphabet `\Mathnormalbold' in
version `nor
mal'
(Font)                  T1/Merriwthr-OsF/b/it --> T1/Merriwthr-OsF/b/it
on inpu
t line 2863.
LaTeX Font Info:    Overwriting math alphabet `\Mathnormalbold' in
version `bol
d'
(Font)                  T1/Merriwthr-OsF/b/it --> T1/Merriwthr-OsF/b/it
on inpu
```

```
t line 2863.
LaTeX Font Info:    Overwriting symbol font `mtletterfont' in version
`normal'
(Font)                  T1/Merriwthr-OsF/m/it --> T1/Merriwthr-OsF/m/it
on inpu
t line 2863.
LaTeX Font Info:    Overwriting symbol font `mtletterfont' in version
`bold'
(Font)                  T1/Merriwthr-OsF/m/it --> T1/Merriwthr-OsF/b/it
on inpu
t line 2863.
LaTeX Font Info:    Overwriting symbol font `mtoperatorfont' in version
`normal
'
(Font)                  T1/Merriwthr-OsF/m/n --> T1/Merriwthr-OsF/m/n on
input
line 2863.
LaTeX Font Info:    Overwriting symbol font `mtoperatorfont' in version
`bold'
(Font)                  T1/Merriwthr-OsF/m/n --> T1/Merriwthr-OsF/b/n on
input
line 2863.
LaTeX Font Info:    Overwriting math alphabet `\Mathbf' in version
`normal'
(Font)                  T1/Merriwthr-OsF/b/n --> T1/Merriwthr-OsF/b/n on
input
line 2863.
LaTeX Font Info:    Overwriting math alphabet `\Mathbf' in version `bold'
(Font)                  T1/Merriwthr-OsF/b/n --> T1/Merriwthr-OsF/b/n on
input
line 2863.
LaTeX Font Info:    Overwriting math alphabet `\Mathit' in version
`normal'
(Font)                  T1/Merriwthr-OsF/m/it --> T1/Merriwthr-OsF/m/it
on inpu
t line 2863.
LaTeX Font Info:    Overwriting math alphabet `\Mathit' in version `bold'
(Font)                  T1/Merriwthr-OsF/m/it --> T1/Merriwthr-OsF/b/it
on inpu
t line 2863.
LaTeX Font Info:    Overwriting math alphabet `\Mathsf' in version
`normal'
(Font)                  T1/MerriwthrSans-OsF/m/n --> T1/MerriwthrSans-
OsF/m/n o
n input line 2863.
LaTeX Font Info:    Overwriting math alphabet `\Mathsf' in version `bold'
(Font)                  T1/MerriwthrSans-OsF/m/n --> T1/MerriwthrSans-
OsF/b/n o
n input line 2863.
LaTeX Font Info:    Overwriting math alphabet `\Mathtt' in version
`normal'
(Font)                  T1/lmtt/m/n --> T1/lmtt/m/n on input line 2863.
LaTeX Font Info:    Overwriting math alphabet `\Mathtt' in version `bold'
(Font)                  T1/lmtt/m/n --> T1/lmtt/b/n on input line 2863.
```

```
(   mathastext:   ) Latin letters in the `normal', resp. `bold',
(   mathastext:   ) math versions are now set up to use the fonts
(   mathastext:   ) T1/Merriwthr-OsF/m/it, resp. T1/Merriwthr-OsF/b/it.
(   mathastext:   ) Other characters (digits, ...) and \log-like names
will be
(   mathastext:   ) typeset with the n shape.
(   mathastext:   ) \hbar
(   mathastext:   ) minus as endash
(   mathastext:   ) The italic option is in effect.
(   mathastext:   ) \HUGE has been (re)-defined.
(   mathastext:   ) mathastext has declared larger sizes for subscripts.
(   mathastext:   ) To keep LaTeX defaults, use option
`defaultmathsizes'.

Package mathastext Info: Loading is complete.  You can now use
\Mathastext to
(mathastext)             modify the normal and bold math versions.  Use
it
(mathastext)             with optional argument or use \MTDeclareVersion
to
(mathastext)             declare additional math versions.
) (c:/texlive/2024/texmf-dist/tex/latex/relsize/relsize.sty
Package: relsize 2013/03/29 ver 4.1
) (c:/texlive/2024/texmf-dist/tex/latex/ragged2e/ragged2e.sty
Package: ragged2e 2023/06/22 v3.6 ragged2e Package
\CenteringLeftskip=\skip52
\RaggedLeftLeftskip=\skip53
\RaggedRightLeftskip=\skip54
\CenteringRightskip=\skip55
\RaggedLeftRightskip=\skip56
\RaggedRightRightskip=\skip57
\CenteringParfillskip=\skip58
\RaggedLeftParfillskip=\skip59
\RaggedRightParfillskip=\skip60
\JustifyingParfillskip=\skip61
\CenteringParindent=\skip62
\RaggedLeftParindent=\skip63
\RaggedRightParindent=\skip64
\JustifyingParindent=\skip65
) (c:/texlive/2024/texmf-dist/tex/latex/xcolor/xcolor.sty
Package: xcolor 2023/11/15 v3.01 LaTeX color extensions (UK)
(c:/texlive/2024/texmf-dist/tex/latex/graphics-cfg/color.cfg
File: color.cfg 2016/01/02 v1.6 sample color configuration
)
Package xcolor Info: Driver file: pdftex.def on input line 274.
(c:/texlive/2024/texmf-dist/tex/latex/graphics-def/pdftex.def
File: pdftex.def 2024/04/13 v1.2c Graphics/color driver for pdftex
) (c:/texlive/2024/texmf-dist/tex/latex/graphics/mathcolor.ltx)
Package xcolor Info: Model `cmy' substituted by `cmy0' on input line
1350.
Package xcolor Info: Model `hsb' substituted by `rgb' on input line 1354.
Package xcolor Info: Model `RGB' extended on input line 1366.
Package xcolor Info: Model `HTML' substituted by `rgb' on input line
1368.
```

```
Package xcolor Info: Model `Hsb' substituted by `hsb' on input line 1369.
Package xcolor Info: Model `tHsb' substituted by `hsb' on input line
1370.
Package xcolor Info: Model `HSB' substituted by `hsb' on input line 1371.
Package xcolor Info: Model `Gray' substituted by `gray' on input line
1372.
Package xcolor Info: Model `wave' substituted by `hsb' on input line
1373.
) (c:/texlive/2024/texmf-dist/tex/latex/colortbl/colortbl.sty
Package: colortbl 2024/07/06 v1.0i Color table columns (DPC)
(c:/texlive/2024/texmf-dist/tex/latex/tools/array.sty
Package: array 2024/06/14 v2.6d Tabular extension package (FMi)
\col@sep=\dimen143
\ar@mcellbox=\box53
\extrarowheight=\dimen144
\NC@list=\toks28
\extratabsurround=\skip66
\backup@length=\skip67
\ar@cellbox=\box54
)
\everycr=\toks29
\minrowclearance=\skip68
\rownum=\count272
) (c:/texlive/2024/texmf-dist/tex/latex/graphics/graphicx.sty
Package: graphicx 2021/09/16 v1.2d Enhanced LaTeX Graphics (DPC,SPQR)
(c:/texlive/2024/texmf-dist/tex/latex/graphics/graphics.sty
Package: graphics 2024/05/23 v1.4g Standard LaTeX Graphics (DPC,SPQR)
(c:/texlive/2024/texmf-dist/tex/latex/graphics/trig.sty
Package: trig 2023/12/02 v1.11 sin cos tan (DPC)
) (c:/texlive/2024/texmf-dist/tex/latex/graphics-cfg/graphics.cfg
File: graphics.cfg 2016/06/04 v1.11 sample graphics configuration
)
Package graphics Info: Driver file: pdftex.def on input line 106.
)
\Gin@req@height=\dimen145
\Gin@req@width=\dimen146
) (c:/texlive/2024/texmf-dist/tex/latex/xpatch/xpatch.sty
(c:/texlive/2024/texm
f-dist/tex/latex/l3kernel/expl3.sty
Package: expl3 2024-05-27 L3 programming layer (loader)
(c:/texlive/2024/texmf-dist/tex/latex/l3backend/l3backend-pdftex.def
File: l3backend-pdftex.def 2024-05-08 L3 backend support: PDF output
(pdfTeX)
\l__color_backend_stack_int=\count273
\l__pdf_internal_box=\box55
))
Package: xpatch 2020/03/25 v0.3a Extending etoolbox patching commands
(c:/texlive/2024/texmf-dist/tex/latex/l3packages/xparse/xparse.sty
Package: xparse 2024-05-08 L3 Experimental document command parser
)) (c:/texlive/2024/texmf-dist/tex/latex/environ/environ.sty
Package: environ 2014/05/04 v0.3 A new way to define environments
(c:/texlive/2024/texmf-dist/tex/latex/trimspaces/trimspaces.sty
Package: trimspaces 2009/09/17 v1.1 Trim spaces around a token list
)
```

```
\@envbody=\toks30
) (c:/texlive/2024/texmf-dist/tex/latex/lastpage/lastpage.sty
Package: lastpage 2024/07/07 v2.1c lastpage: 2.09 or 2e? (HMM)
(c:/texlive/2024/texmf-dist/tex/latex/lastpage/lastpage2e.sty
Package: lastpage2e 2024/07/07 v2.1c Decide which 2e lastpage version to
use (H
MM)
(c:/texlive/2024/texmf-dist/tex/latex/lastpage/lastpagemodern.sty
Package: lastpagemodern 2024-07-07 v2.1c Refers to last page's name (HMM;
JPG)
\c@lastpagecount=\count274
)
)) (c:/texlive/2024/texmf-dist/tex/latex/graphics/rotating.sty
Package: rotating 2016/08/11 v2.16d rotated objects in LaTeX
(c:/texlive/2024/texmf-dist/tex/latex/base/ifthen.sty
Package: ifthen 2024/03/16 v1.1e Standard LaTeX ifthen package (DPC)
)
\c@r@tfl@t=\count275
\rotFPtop=\skip69
\rotFPbot=\skip70
\rot@float@box=\box56
\rot@mess@toks=\toks31
) (c:/texlive/2024/texmf-dist/tex/latex/graphics/lscape.sty
Package: lscape 2020/05/28 v3.02 Landscape Pages (DPC)
) (c:/texlive/2024/texmf-dist/tex/latex/tools/afterpage.sty
Package: afterpage 2023/07/04 v1.08 After-Page Package (DPC)
\AP@output=\toks32
\AP@partial=\box57
\AP@footins=\box58
) (c:/texlive/2024/texmf-dist/tex/latex/textpos/textpos.sty
Package: textpos 2022/07/23 v1.10.1
Package textpos Info: choosing support for LaTeX3 on input line 60.
\TP@textbox=\box59
\TP@holdbox=\box60
\TPHorizModule=\dimen147
\TPVertModule=\dimen148
\TP@margin=\dimen149
\TP@absmargin=\dimen150
Grid set 16 x 16 = 37.34424pt x 52.81541pt
\TPboxrulesize=\dimen151
\TP@ox=\dimen152
\TP@oy=\dimen153
\TP@tbargs=\toks33
TextBlockOrigin set to 0pt x 0pt
) (c:/texlive/2024/texmf-dist/tex/latex/url/url.sty
\Urlmuskip=\muskip20
Package: url 2013/09/16  ver 3.4  Verb mode for urls, etc.
) (c:/texlive/2024/texmf-dist/tex/latex/newfloat/newfloat.sty
Package: newfloat 2023/10/01 v1.2 Defining new floating environments (AR)
Package newfloat Info: `rotating' package detected.
) (c:/texlive/2024/texmf-dist/tex/latex/mdframed/mdframed.sty
Package: mdframed 2013/07/01 1.9b: mdframed
(c:/texlive/2024/texmf-dist/tex/latex/kvoptions/kvoptions.sty
```

```
Package: kvoptions 2022-06-15 v3.15 Key value format for package options
(HO)
(c:/texlive/2024/texmf-dist/tex/generic/ltxcmds/ltxcmds.sty
Package: ltxcmds 2023-12-04 v1.26 LaTeX kernel commands for general use
(HO)
) (c:/texlive/2024/texmf-dist/tex/latex/kvsetkeys/kvsetkeys.sty
Package: kvsetkeys 2022-10-05 v1.19 Key value parser (HO)
)) (c:/texlive/2024/texmf-dist/tex/latex/zref/zref-abspage.sty
Package: zref-abspage 2023-09-14 v2.35 Module abspage for zref (HO)
(c:/texlive/2024/texmf-dist/tex/latex/zref/zref-base.sty
Package: zref-base 2023-09-14 v2.35 Module base for zref (HO)
(c:/texlive/2024/texmf-dist/tex/generic/infwarerr/infwarerr.sty
Package: infwarerr 2019/12/03 v1.5 Providing info/warning/error messages
(HO)
) (c:/texlive/2024/texmf-dist/tex/generic/kvdefinekeys/kvdefinekeys.sty
Package: kvdefinekeys 2019-12-19 v1.6 Define keys (HO)
) (c:/texlive/2024/texmf-dist/tex/generic/pdftexcmds/pdftexcmds.sty
Package: pdftexcmds 2020-06-27 v0.33 Utility functions of pdfTeX for
LuaTeX (HO
)
Package pdftexcmds Info: \pdf@primitive is available.
Package pdftexcmds Info: \pdf@ifprimitive is available.
Package pdftexcmds Info: \pdfdraftmode found.
) (c:/texlive/2024/texmf-dist/tex/generic/etexcmds/etexcmds.sty
Package: etexcmds 2019/12/15 v1.7 Avoid name clashes with e-TeX commands
(HO)
) (c:/texlive/2024/texmf-dist/tex/latex/auxhook/auxhook.sty
Package: auxhook 2019-12-17 v1.6 Hooks for auxiliary files (HO)
)
Package zref Info: New property list: main on input line 767.
Package zref Info: New property: default on input line 768.
Package zref Info: New property: page on input line 769.
)
\c@abspage=\count276
Package zref Info: New property: abspage on input line 67.
) (c:/texlive/2024/texmf-dist/tex/latex/needspace/needspace.sty
Package: needspace 2010/09/12 v1.3d reserve vertical space
)
\mdf@templength=\skip71
\c@mdf@globalstyle@cnt=\count277
\mdf@skipabove@length=\skip72
\mdf@skipbelow@length=\skip73
\mdf@leftmargin@length=\skip74
\mdf@rightmargin@length=\skip75
\mdf@innerleftmargin@length=\skip76
\mdf@innerrightmargin@length=\skip77
\mdf@innertopmargin@length=\skip78
\mdf@innerbottommargin@length=\skip79
\mdf@splittopskip@length=\skip80
\mdf@splitbottomskip@length=\skip81
\mdf@outermargin@length=\skip82
\mdf@innermargin@length=\skip83
\mdf@linewidth@length=\skip84
\mdf@innerlinewidth@length=\skip85
```

```
\mdf@middlelinewidth@length=\skip86
\mdf@outerlinewidth@length=\skip87
\mdf@roundcorner@length=\skip88
\mdf@footenotedistance@length=\skip89
\mdf@userdefinedwidth@length=\skip90
\mdf@needspace@length=\skip91
\mdf@frametitleaboveskip@length=\skip92
\mdf@frametitlebelowskip@length=\skip93
\mdf@frametitlerulewidth@length=\skip94
\mdf@frametitleleftmargin@length=\skip95
\mdf@frametitlerightmargin@length=\skip96
\mdf@shadowsize@length=\skip97
\mdf@extratopheight@length=\skip98
\mdf@subtitleabovelinewidth@length=\skip99
\mdf@subtitlebelowlinewidth@length=\skip100
\mdf@subtitleaboveskip@length=\skip101
\mdf@subtitlebelowskip@length=\skip102
\mdf@subtitleinneraboveskip@length=\skip103
\mdf@subtitleinnerbelowskip@length=\skip104
\mdf@subsubtitleabovelinewidth@length=\skip105
\mdf@subsubtitlebelowlinewidth@length=\skip106
\mdf@subsubtitleaboveskip@length=\skip107
\mdf@subsubtitlebelowskip@length=\skip108
\mdf@subsubtitleinneraboveskip@length=\skip109
\mdf@subsubtitleinnerbelowskip@length=\skip110
(c:/texlive/2024/texmf-dist/tex/latex/mdframed/md-frame-0.mdf
File: md-frame-0.mdf 2013/07/01\ 1.9b: md-frame-0
)
\mdf@frametitlebox=\box61
\mdf@footnotebox=\box62
\mdf@splitbox@one=\box63
\mdf@splitbox@two=\box64
\mdf@splitbox@save=\box65
\mdfsplitboxwidth=\skip111
\mdfsplitboxtotalwidth=\skip112
\mdfsplitboxheight=\skip113
\mdfsplitboxdepth=\skip114
\mdfsplitboxtotalheight=\skip115
\mdfframetitleboxwidth=\skip116
\mdfframetitleboxtotalwidth=\skip117
\mdfframetitleboxheight=\skip118
\mdfframetitleboxdepth=\skip119
\mdfframetitleboxtotalheight=\skip120
\mdffootnoteboxwidth=\skip121
\mdffootnoteboxtotalwidth=\skip122
\mdffootnoteboxheight=\skip123
\mdffootnoteboxdepth=\skip124
\mdffootnoteboxtotalheight=\skip125
\mdftotallinewidth=\skip126
\mdfboundingboxwidth=\skip127
\mdfboundingboxtotalwidth=\skip128
\mdfboundingboxheight=\skip129
\mdfboundingboxdepth=\skip130
\mdfboundingboxtotalheight=\skip131
```

```
\mdf@freevspace@length=\skip132
\mdf@horizontalwidthofbox@length=\skip133
\mdf@verticalmarginwhole@length=\skip134
\mdf@horizontalspaceofbox=\skip135
\mdfsubtitleheight=\skip136
\mdfsubsubtitleheight=\skip137
\c@mdfcountframes=\count278

****** mdframed patching \endmdf@trivlist

****** -- success******

\mdf@envdepth=\count279
\c@mdf@env@i=\count280
\c@mdf@env@ii=\count281
\c@mdf@zref@counter=\count282
Package zref Info: New property: mdf@pagevalue on input line 895.
) (c:/texlive/2024/texmf-dist/tex/latex/titlesec/titlesec.sty
Package: titlesec 2023/10/27 v2.16 Sectioning titles
\ttl@box=\box66
\beforetitleunit=\skip138
\aftertitleunit=\skip139
\ttl@plus=\dimen154
\ttl@minus=\dimen155
\ttl@toksa=\toks34
\titlewidth=\dimen156
\titlewidthlast=\dimen157
\titlewidthfirst=\dimen158
) (c:/texlive/2024/texmf-dist/tex/latex/koma-script/scrextend.sty
Package: scrextend 2023/07/07 v3.41 KOMA-Script package (extend other
classes w
ith features of KOMA-Script classes)
(c:/texlive/2024/texmf-dist/tex/latex/koma-script/scrkbase.sty
Package: scrkbase 2023/07/07 v3.41 KOMA-Script package (KOMA-Script-
dependent b
asics and keyval usage)
(c:/texlive/2024/texmf-dist/tex/latex/koma-script/scrbase.sty
Package: scrbase 2023/07/07 v3.41 KOMA-Script package (KOMA-Script-
independent
basics and keyval usage)
(c:/texlive/2024/texmf-dist/tex/latex/koma-script/scrlfile.sty
Package: scrlfile 2023/07/07 v3.41 KOMA-Script package (file load hooks)
(c:/texlive/2024/texmf-dist/tex/latex/koma-script/scrlfile-hook.sty
Package: scrlfile-hook 2023/07/07 v3.41 KOMA-Script package (using LaTeX
hooks)

(c:/texlive/2024/texmf-dist/tex/latex/koma-script/scrlogo.sty
Package: scrlogo 2023/07/07 v3.41 KOMA-Script package (logo)
)))
Applying: [2021/05/01] Usage of raw or classic option list on input line
252.
Already applied: [0000/00/00] Usage of raw or classic option list on
input line
 368.
```

```
))
Package scrextend Info: unexpected definition of `\@makefnmark'.
(scrextend)             Trying to patch it on input line 1762.
Package scrextend Info: patch seems to be successfull on input line 1762.
)

LaTeX Font Warning: Font shape `T1/cmr/m/n' in size <7.5> not available
(Font)              size <7> substituted on input line 69.

(c:/texlive/2024/texmf-dist/tex/latex/tools/calc.sty
Package: calc 2023/07/08 v4.3 Infix arithmetic (KKT,FJ)
\calc@Acount=\count283
\calc@Bcount=\count284
\calc@Adimen=\dimen159
\calc@Bdimen=\dimen160
\calc@Askip=\skip140
\calc@Bskip=\skip141
LaTeX Info: Redefining \setlength on input line 80.
LaTeX Info: Redefining \addtolength on input line 81.
\calc@Ccount=\count285
\calc@Cskip=\skip142
) (c:/texlive/2024/texmf-dist/tex/latex/geometry/geometry.sty
Package: geometry 2020/01/02 v5.9 Page Geometry
(c:/texlive/2024/texmf-dist/tex/generic/iftex/ifvtex.sty
Package: ifvtex 2019/10/25 v1.7 ifvtex legacy package. Use iftex instead.
)
\Gm@cnth=\count286
\Gm@cntv=\count287
\c@Gm@tempcnt=\count288
\Gm@bindingoffset=\dimen161
\Gm@wd@mp=\dimen162
\Gm@odd@mp=\dimen163
\Gm@even@mp=\dimen164
\Gm@layoutwidth=\dimen165
\Gm@layoutheight=\dimen166
\Gm@layouthoffset=\dimen167
\Gm@layoutvoffset=\dimen168
\Gm@dimlist=\toks35
) (c:/texlive/2024/texmf-dist/tex/latex/preprint/authblk.sty
Package: authblk 2001/02/27 1.3 (PWD)
\affilsep=\skip143
\@affilsep=\skip144
\c@Maxaffil=\count289
\c@authors=\count290
\c@affil=\count291
) (c:/texlive/2024/texmf-dist/tex/latex/footmisc/footmisc.sty
Package: footmisc 2023/07/05 v6.0f a miscellany of footnote facilities
\FN@temptoken=\toks36
\footnotemargin=\dimen169
\@outputbox@depth=\dimen170
Package footmisc Info: Declaring symbol style bringhurst on input line
696.
Package footmisc Info: Declaring symbol style chicago on input line 704.
Package footmisc Info: Declaring symbol style wiley on input line 713.
```

```
Package footmisc Info: Declaring symbol style lamport-robust on input
line 724.

Package footmisc Info: Declaring symbol style lamport* on input line 744.
Package footmisc Info: Declaring symbol style lamport*-robust on input
line 765
.
) (c:/texlive/2024/texmf-dist/tex/latex/fancyhdr/fancyhdr.sty
Package: fancyhdr 2024/07/23 v4.3.1 Extensive control of page headers and
foote
rs
\f@nch@headwidth=\skip145
\f@nch@O@elh=\skip146
\f@nch@O@erh=\skip147
\f@nch@O@olh=\skip148
\f@nch@O@orh=\skip149
\f@nch@O@elf=\skip150
\f@nch@O@erf=\skip151
\f@nch@O@olf=\skip152
\f@nch@O@orf=\skip153
) (c:/texlive/2024/texmf-dist/tex/generic/alphalph/alphalph.sty
Package: alphalph 2019/12/09 v2.6 Convert numbers to letters (HO)
(c:/texlive/2024/texmf-dist/tex/generic/intcalc/intcalc.sty
Package: intcalc 2019/12/15 v1.3 Expandable calculations with integers
(HO)
))
\c@authorfn=\count292
(c:/texlive/2024/texmf-dist/tex/latex/abstract/abstract.sty
Package: abstract 2009/06/08 v1.2a configurable abstracts
\abstitleskip=\skip154
\absleftindent=\skip155
\absrightindent=\skip156
\absparindent=\skip157
\absparsep=\skip158
)
Package newfloat Info: New float `keypoints' with options
`placement=t!,name=kp
t' on input line 291.
\c@keypoints=\count293
\newfloat@ftype=\count294
Package newfloat Info: float type `keypoints'=8 on input line 291.
(c:/texlive/2024/texmf-dist/tex/latex/enumitem/enumitem.sty
Package: enumitem 2019/06/20 v3.9 Customized lists
\labelindent=\skip159
\enit@outerparindent=\dimen171
\enit@toks=\toks37
\enit@inbox=\box67
\enit@count@id=\count295
\enitdp@description=\count296
) (c:/texlive/2024/texmf-dist/tex/latex/quoting/quoting.sty
Package: quoting 2014/01/28 v0.1c Consolidated environment for displayed
text
\quo@toppartop=\skip160
) (c:/texlive/2024/texmf-dist/tex/latex/sttools/stfloats.sty
```

```
Package: stfloats 2017/03/27 v3.3 Improve float mechanism and
baselineskip sett
ings
\@dblbotnum=\count297
\c@dblbotnumber=\count298
) (c:/texlive/2024/texmf-dist/tex/latex/booktabs/booktabs.sty
Package: booktabs 2020/01/12 v1.61803398 Publication quality tables
\heavyrulewidth=\dimen172
\lightrulewidth=\dimen173
\cmidrulewidth=\dimen174
\belowrulesep=\dimen175
\belowbottomsep=\dimen176
\aboverulesep=\dimen177
\abovetopsep=\dimen178
\cmidrulesep=\dimen179
\cmidrulekern=\dimen180
\defaultaddspace=\dimen181
\@cmidla=\count299
\@cmidlb=\count300
\@aboverulesep=\dimen182
\@belowrulesep=\dimen183
\@thisruleclass=\count301
\@lastruleclass=\count302
\@thisrulewidth=\dimen184
) (c:/texlive/2024/texmf-dist/tex/latex/tools/tabularx.sty
Package: tabularx 2023/12/11 v2.12a `tabularx' package (DPC)
\TX@col@width=\dimen185
\TX@old@table=\dimen186
\TX@old@col=\dimen187
\TX@target=\dimen188
\TX@delta=\dimen189
\TX@cols=\count303
\TX@ftn=\toks38
)
\enitdp@tablenotes=\count304
(c:/texlive/2024/texmf-dist/tex/latex/caption/caption.sty
Package: caption 2023/08/05 v3.6o Customizing captions (AR)
(c:/texlive/2024/texmf-dist/tex/latex/caption/caption3.sty
Package: caption3 2023/07/31 v2.4d caption3 kernel (AR)
\caption@tempdima=\dimen190
\captionmargin=\dimen191
\caption@leftmargin=\dimen192
\caption@rightmargin=\dimen193
\caption@width=\dimen194
\caption@indent=\dimen195
\caption@parindent=\dimen196
\caption@hangindent=\dimen197
Package caption Info: Standard document class detected.
)
\c@caption@flags=\count305
\c@continuedfloat=\count306
Package caption Info: rotating package is loaded.
Package caption Info: scrextend package is loaded.
\caption@addmargin@hsize=\dimen198
```

```
\caption@addmargin@linewidth=\dimen199
) (c:/texlive/2024/texmf-dist/tex/latex/natbib/natbib.sty
Package: natbib 2010/09/13 8.31b (PWD, AO)
\bibhang=\skip161
\bibsep=\skip162
LaTeX Info: Redefining \cite on input line 694.
\c@NAT@ctr=\count307
)) (c:/texlive/2024/texmf-dist/tex/latex/siunitx/siunitx.sty
Package: siunitx 2024-06-24 v3.3.19 A comprehensive (SI) units package
\l__siunitx_number_uncert_offset_int=\count308
\l__siunitx_number_exponent_fixed_int=\count309
\l__siunitx_number_min_decimal_int=\count310
\l__siunitx_number_min_integer_int=\count311
\l__siunitx_number_round_precision_int=\count312
\l__siunitx_number_lower_threshold_int=\count313
\l__siunitx_number_upper_threshold_int=\count314
\l__siunitx_number_group_first_int=\count315
\l__siunitx_number_group_size_int=\count316
\l__siunitx_number_group_minimum_int=\count317
\l__siunitx_angle_tmp_dim=\dimen256
\l__siunitx_angle_marker_box=\box68
\l__siunitx_angle_unit_box=\box69
\l__siunitx_compound_count_int=\count318
(c:/texlive/2024/texmf-dist/tex/latex/translations/translations.sty
Package: translations 2022/02/05 v1.12 internationalization of LaTeX2e
packages
 (CN)
) (c:/texlive/2024/texmf-dist/tex/latex/amsmath/amstext.sty
Package: amstext 2021/08/26 v2.01 AMS text
(c:/texlive/2024/texmf-dist/tex/latex/amsmath/amsgen.sty
File: amsgen.sty 1999/11/30 v2.0 generic functions
\@emptytoks=\toks39
\ex@=\dimen257
))
\l__siunitx_table_tmp_box=\box70
\l__siunitx_table_tmp_dim=\dimen258
\l__siunitx_table_column_width_dim=\dimen259
\l__siunitx_table_integer_box=\box71
\l__siunitx_table_decimal_box=\box72
\l__siunitx_table_uncert_box=\box73
\l__siunitx_table_before_box=\box74
\l__siunitx_table_after_box=\box75
\l__siunitx_table_before_dim=\dimen260
\l__siunitx_table_carry_dim=\dimen261
\l__siunitx_unit_tmp_int=\count319
\l__siunitx_unit_position_int=\count320
\l__siunitx_unit_total_int=\count321
) (c:/texlive/2024/texmf-dist/tex/latex/listings/listings.sty
\lst@mode=\count322
\lst@gtempboxa=\box76
\lst@token=\toks40
\lst@length=\count323
\lst@currlwidth=\dimen262
\lst@column=\count324
```

```
\lst@pos=\count325
\lst@lostspace=\dimen263
\lst@width=\dimen264
\lst@newlines=\count326
\lst@lineno=\count327
\lst@maxwidth=\dimen265
(c:/texlive/2024/texmf-dist/tex/latex/listings/lstpatch.sty
File: lstpatch.sty 2024/05/25 1.10b (Carsten Heinz)
) (c:/texlive/2024/texmf-dist/tex/latex/listings/lstmisc.sty
File: lstmisc.sty 2024/05/25 1.10b (Carsten Heinz)
\c@lstnumber=\count328
\lst@skipnumbers=\count329
\lst@framebox=\box77
) (c:/texlive/2024/texmf-dist/tex/latex/listings/listings.cfg
File: listings.cfg 2024/05/25 1.10b listings configuration
))
Package: listings 2024/05/25 1.10b (Carsten Heinz)
(c:/texlive/2024/texmf-dist/tex/latex/tools/multicol.sty
Package: multicol 2024/05/23 v1.9h multicolumn formatting (FMi)
\c@tracingmulticols=\count330
\mult@box=\box78
\multicol@leftmargin=\dimen266
\c@unbalance=\count331
\c@collectmore=\count332
\doublecol@number=\count333
\multicoltolerance=\count334
\multicolpretolerance=\count335
\full@width=\dimen267
\page@free=\dimen268
\premulticols=\dimen269
\postmulticols=\dimen270
\multicolsep=\skip163
\multicolbaselineskip=\skip164
\partial@page=\box79
\last@line=\box80
\mc@boxedresult=\box81
\maxbalancingoverflow=\dimen271
\mult@rightbox=\box82
\mult@grightbox=\box83
\mult@firstbox=\box84
\mult@gfirstbox=\box85
\@tempa=\box86
\@tempa=\box87
\@tempa=\box88
\@tempa=\box89
\@tempa=\box90
\@tempa=\box91
\@tempa=\box92
\@tempa=\box93
\@tempa=\box94
\@tempa=\box95
\@tempa=\box96
\@tempa=\box97
\@tempa=\box98
```

```
\@tempa=\box99
\@tempa=\box100
\@tempa=\box101
\@tempa=\box102
\@tempa=\box103
\@tempa=\box104
\@tempa=\box105
\@tempa=\box106
\@tempa=\box107
\@tempa=\box108
\@tempa=\box109
\@tempa=\box110
\@tempa=\box111
\@tempa=\box112
\@tempa=\box113
\@tempa=\box114
\@tempa=\box115
\@tempa=\box116
\@tempa=\box117
\@tempa=\box118
\@tempa=\box119
\@tempa=\box120
\@tempa=\box121
\c@minrows=\count336
\c@columnbadness=\count337
\c@finalcolumnbadness=\count338
\last@try=\dimen272
\multicolovershoot=\dimen273
\multicolundershoot=\dimen274
\mult@nat@firstbox=\box122
\colbreak@box=\box123
\mc@col@check@num=\count339
) (c:/texlive/2024/texmf-dist/tex/latex/placeins/placeins.sty
Package: placeins 2005/04/18  v 2.2
) (c:/texlive/2024/texmf-dist/tex/latex/hyperref/hyperref.sty
Package: hyperref 2024-07-10 v7.01j Hypertext links for LaTeX
(c:/texlive/2024/texmf-dist/tex/generic/pdfescape/pdfescape.sty
Package: pdfescape 2019/12/09 v1.15 Implements pdfTeX's escape features
(HO)
) (c:/texlive/2024/texmf-dist/tex/latex/hycolor/hycolor.sty
Package: hycolor 2020-01-27 v1.10 Color options for hyperref/bookmark
(HO)
) (c:/texlive/2024/texmf-dist/tex/latex/hyperref/nameref.sty
Package: nameref 2023-11-26 v2.56 Cross-referencing by name of section
(c:/texlive/2024/texmf-dist/tex/latex/refcount/refcount.sty
Package: refcount 2019/12/15 v3.6 Data extraction from label references
(HO)
) (c:/texlive/2024/texmf-
dist/tex/generic/gettitlestring/gettitlestring.sty
Package: gettitlestring 2019/12/15 v1.6 Cleanup title references (HO)
)
\c@section@level=\count340
) (c:/texlive/2024/texmf-dist/tex/generic/stringenc/stringenc.sty
```

```
Package: stringenc 2019/11/29 v1.12 Convert strings between diff.
encodings (HO
)
)
\@linkdim=\dimen275
\Hy@linkcounter=\count341
\Hy@pagecounter=\count342
(c:/texlive/2024/texmf-dist/tex/latex/hyperref/pd1enc.def
File: pd1enc.def 2024-07-10 v7.01j Hyperref: PDFDocEncoding definition
(HO)
Now handling font encoding PD1 ...
... no UTF-8 mapping file for font encoding PD1
)
\Hy@SavedSpaceFactor=\count343
(c:/texlive/2024/texmf-dist/tex/latex/hyperref/puenc.def
File: puenc.def 2024-07-10 v7.01j Hyperref: PDF Unicode definition (HO)
Now handling font encoding PU ...
... no UTF-8 mapping file for font encoding PU
)
Package hyperref Info: Hyper figures OFF on input line 4157.
Package hyperref Info: Link nesting OFF on input line 4162.
Package hyperref Info: Hyper index ON on input line 4165.
Package hyperref Info: Plain pages OFF on input line 4172.
Package hyperref Info: Backreferencing OFF on input line 4177.
Package hyperref Info: Implicit mode ON; LaTeX internals redefined.
Package hyperref Info: Bookmarks ON on input line 4424.
\c@Hy@tempcnt=\count344
LaTeX Info: Redefining \url on input line 4763.
\XeTeXLinkMargin=\dimen276
(c:/texlive/2024/texmf-dist/tex/generic/bitset/bitset.sty
Package: bitset 2019/12/09 v1.3 Handle bit-vector datatype (HO)
(c:/texlive/2024/texmf-dist/tex/generic/bigintcalc/bigintcalc.sty
Package: bigintcalc 2019/12/15 v1.5 Expandable calculations on big
integers (HO
)
))
\Fld@menulength=\count345
\Field@Width=\dimen277
\Fld@charsize=\dimen278
Package hyperref Info: Hyper figures OFF on input line 6042.
Package hyperref Info: Link nesting OFF on input line 6047.
Package hyperref Info: Hyper index ON on input line 6050.
Package hyperref Info: backreferencing OFF on input line 6057.
Package hyperref Info: Link coloring OFF on input line 6062.
Package hyperref Info: Link coloring with OCG OFF on input line 6067.
Package hyperref Info: PDF/A mode OFF on input line 6072.
(c:/texlive/2024/texmf-dist/tex/latex/base/atbegshi-ltx.sty
Package: atbegshi-ltx 2021/01/10 v1.0c Emulation of the original atbegshi
package with kernel methods
)
\Hy@abspage=\count346
\c@Item=\count347
\c@Hfootnote=\count348
)
```

```
Package hyperref Info: Driver (autodetected): hpdftex.
(c:/texlive/2024/texmf-dist/tex/latex/hyperref/hpdftex.def
File: hpdftex.def 2024-07-10 v7.01j Hyperref driver for pdfTeX
(c:/texlive/2024/texmf-dist/tex/latex/base/atveryend-ltx.sty
Package: atveryend-ltx 2020/08/19 v1.0a Emulation of the original
atveryend pac
kage
with kernel methods
)
\HyAnn@Count=\count349
\Fld@listcount=\count350
\c@bookmark@seq@number=\count351
(c:/texlive/2024/texmf-dist/tex/latex/rerunfilecheck/rerunfilecheck.sty
Package: rerunfilecheck 2022-07-10 v1.10 Rerun checks for auxiliary files
(HO)
(c:/texlive/2024/texmf-dist/tex/generic/uniquecounter/uniquecounter.sty
Package: uniquecounter 2019/12/15 v1.4 Provide unlimited unique counter
(HO)
)
Package uniquecounter Info: New unique counter `rerunfilecheck' on input
line 2
85.
)
\Hy@SectionHShift=\skip165
) (c:/texlive/2024/texmf-dist/tex/latex/orcidlink/orcidlink.sty
Package: orcidlink 2024/06/26 v1.1.0 Support ORCID's three different ID
formats
.
(c:/texlive/2024/texmf-dist/tex/latex/pgf/frontendlayer/tikz.sty
(c:/texlive/20
24/texmf-dist/tex/latex/pgf/basiclayer/pgf.sty (c:/texlive/2024/texmf-
dist/tex/
latex/pgf/utilities/pgfrcs.sty (c:/texlive/2024/texmf-
dist/tex/generic/pgf/util
ities/pgfutil-common.tex
\pgfutil@everybye=\toks41
\pgfutil@tempdima=\dimen279
\pgfutil@tempdimb=\dimen280
) (c:/texlive/2024/texmf-dist/tex/generic/pgf/utilities/pgfutil-latex.def
\pgfutil@abb=\box124
) (c:/texlive/2024/texmf-dist/tex/generic/pgf/utilities/pgfrcs.code.tex
(c:/tex
live/2024/texmf-dist/tex/generic/pgf/pgf.revision.tex)
Package: pgfrcs 2023-01-15 v3.1.10 (3.1.10)
))
Package: pgf 2023-01-15 v3.1.10 (3.1.10)
(c:/texlive/2024/texmf-dist/tex/latex/pgf/basiclayer/pgfcore.sty
(c:/texlive/20
24/texmf-dist/tex/latex/pgf/systemlayer/pgfsys.sty
(c:/texlive/2024/texmf-dist/
tex/generic/pgf/systemlayer/pgfsys.code.tex
Package: pgfsys 2023-01-15 v3.1.10 (3.1.10)
(c:/texlive/2024/texmf-dist/tex/generic/pgf/utilities/pgfkeys.code.tex
\pgfkeys@pathtoks=\toks42
```

```
\pgfkeys@temptoks=\toks43

(c:/texlive/2024/texmf-
dist/tex/generic/pgf/utilities/pgfkeyslibraryfiltered.co
de.tex
\pgfkeys@tmptoks=\toks44
))
\pgf@x=\dimen281
\pgf@y=\dimen282
\pgf@xa=\dimen283
\pgf@ya=\dimen284
\pgf@xb=\dimen285
\pgf@yb=\dimen286
\pgf@xc=\dimen287
\pgf@yc=\dimen288
\pgf@xd=\dimen289
\pgf@yd=\dimen290
\w@pgf@writea=\write3
\r@pgf@reada=\read2
\c@pgf@counta=\count352
\c@pgf@countb=\count353
\c@pgf@countc=\count354
\c@pgf@countd=\count355
\t@pgf@toka=\toks45
\t@pgf@tokb=\toks46
\t@pgf@tokc=\toks47
\pgf@sys@id@count=\count356
(c:/texlive/2024/texmf-dist/tex/generic/pgf/systemlayer/pgf.cfg
File: pgf.cfg 2023-01-15 v3.1.10 (3.1.10)
)
Driver file for pgf: pgfsys-pdftex.def
(c:/texlive/2024/texmf-dist/tex/generic/pgf/systemlayer/pgfsys-pdftex.def
File: pgfsys-pdftex.def 2023-01-15 v3.1.10 (3.1.10)
(c:/texlive/2024/texmf-dist/tex/generic/pgf/systemlayer/pgfsys-common-
pdf.def
File: pgfsys-common-pdf.def 2023-01-15 v3.1.10 (3.1.10)
)))
(c:/texlive/2024/texmf-
dist/tex/generic/pgf/systemlayer/pgfsyssoftpath.code.tex
File: pgfsyssoftpath.code.tex 2023-01-15 v3.1.10 (3.1.10)
\pgfsyssoftpath@smallbuffer@items=\count357
\pgfsyssoftpath@bigbuffer@items=\count358
)
(c:/texlive/2024/texmf-
dist/tex/generic/pgf/systemlayer/pgfsysprotocol.code.tex
File: pgfsysprotocol.code.tex 2023-01-15 v3.1.10 (3.1.10)
)) (c:/texlive/2024/texmf-
dist/tex/generic/pgf/basiclayer/pgfcore.code.tex
Package: pgfcore 2023-01-15 v3.1.10 (3.1.10)
(c:/texlive/2024/texmf-dist/tex/generic/pgf/math/pgfmath.code.tex
(c:/texlive/2
024/texmf-dist/tex/generic/pgf/math/pgfmathutil.code.tex)
(c:/texlive/2024/texm
f-dist/tex/generic/pgf/math/pgfmathparser.code.tex
```

```
\pgfmath@dimen=\dimen291
\pgfmath@count=\count359
\pgfmath@box=\box125
\pgfmath@toks=\toks48
\pgfmath@stack@operand=\toks49
\pgfmath@stack@operation=\toks50
) (c:/texlive/2024/texmf-
dist/tex/generic/pgf/math/pgfmathfunctions.code.tex)
(c:/texlive/2024/texmf-
dist/tex/generic/pgf/math/pgfmathfunctions.basic.code.te
x)
(c:/texlive/2024/texmf-
dist/tex/generic/pgf/math/pgfmathfunctions.trigonometric
.code.tex)
(c:/texlive/2024/texmf-
dist/tex/generic/pgf/math/pgfmathfunctions.random.code.t
ex)
(c:/texlive/2024/texmf-
dist/tex/generic/pgf/math/pgfmathfunctions.comparison.co
de.tex)
(c:/texlive/2024/texmf-
dist/tex/generic/pgf/math/pgfmathfunctions.base.code.tex
)
(c:/texlive/2024/texmf-
dist/tex/generic/pgf/math/pgfmathfunctions.round.code.te
x)
(c:/texlive/2024/texmf-
dist/tex/generic/pgf/math/pgfmathfunctions.misc.code.tex
)
(c:/texlive/2024/texmf-
dist/tex/generic/pgf/math/pgfmathfunctions.integerarithm
etics.code.tex) (c:/texlive/2024/texmf-
dist/tex/generic/pgf/math/pgfmathcalc.co
de.tex) (c:/texlive/2024/texmf-
dist/tex/generic/pgf/math/pgfmathfloat.code.tex
\c@pgfmathroundto@lastzeros=\count360
)) (c:/texlive/2024/texmf-dist/tex/generic/pgf/math/pgfint.code.tex)
(c:/texliv
e/2024/texmf-dist/tex/generic/pgf/basiclayer/pgfcorepoints.code.tex
File: pgfcorepoints.code.tex 2023-01-15 v3.1.10 (3.1.10)
\pgf@picminx=\dimen292
\pgf@picmaxx=\dimen293
\pgf@picminy=\dimen294
\pgf@picmaxy=\dimen295
\pgf@pathminx=\dimen296
\pgf@pathmaxx=\dimen297
\pgf@pathminy=\dimen298
\pgf@pathmaxy=\dimen299
\pgf@xx=\dimen300
\pgf@xy=\dimen301
\pgf@yx=\dimen302
\pgf@yy=\dimen303
\pgf@zx=\dimen304
\pgf@zy=\dimen305
```

```
)
(c:/texlive/2024/texmf-
dist/tex/generic/pgf/basiclayer/pgfcorepathconstruct.cod
e.tex
File: pgfcorepathconstruct.code.tex 2023-01-15 v3.1.10 (3.1.10)
\pgf@path@lastx=\dimen306
\pgf@path@lasty=\dimen307
)
(c:/texlive/2024/texmf-
dist/tex/generic/pgf/basiclayer/pgfcorepathusage.code.te
x
File: pgfcorepathusage.code.tex 2023-01-15 v3.1.10 (3.1.10)
\pgf@shorten@end@additional=\dimen308
\pgf@shorten@start@additional=\dimen309
) (c:/texlive/2024/texmf-
dist/tex/generic/pgf/basiclayer/pgfcorescopes.code.tex
File: pgfcorescopes.code.tex 2023-01-15 v3.1.10 (3.1.10)
\pgfpic=\box126
\pgf@hbox=\box127
\pgf@layerbox@main=\box128
\pgf@picture@serial@count=\count361
)
(c:/texlive/2024/texmf-
dist/tex/generic/pgf/basiclayer/pgfcoregraphicstate.code
.tex
File: pgfcoregraphicstate.code.tex 2023-01-15 v3.1.10 (3.1.10)
\pgflinewidth=\dimen310
)
(c:/texlive/2024/texmf-
dist/tex/generic/pgf/basiclayer/pgfcoretransformations.c
ode.tex
File: pgfcoretransformations.code.tex 2023-01-15 v3.1.10 (3.1.10)
\pgf@pt@x=\dimen311
\pgf@pt@y=\dimen312
\pgf@pt@temp=\dimen313
) (c:/texlive/2024/texmf-
dist/tex/generic/pgf/basiclayer/pgfcorequick.code.tex
File: pgfcorequick.code.tex 2023-01-15 v3.1.10 (3.1.10)
) (c:/texlive/2024/texmf-
dist/tex/generic/pgf/basiclayer/pgfcoreobjects.code.te
x
File: pgfcoreobjects.code.tex 2023-01-15 v3.1.10 (3.1.10)
)
(c:/texlive/2024/texmf-
dist/tex/generic/pgf/basiclayer/pgfcorepathprocessing.co
de.tex
File: pgfcorepathprocessing.code.tex 2023-01-15 v3.1.10 (3.1.10)
) (c:/texlive/2024/texmf-
dist/tex/generic/pgf/basiclayer/pgfcorearrows.code.tex
File: pgfcorearrows.code.tex 2023-01-15 v3.1.10 (3.1.10)
\pgfarrowsep=\dimen314
) (c:/texlive/2024/texmf-
dist/tex/generic/pgf/basiclayer/pgfcoreshade.code.tex
File: pgfcoreshade.code.tex 2023-01-15 v3.1.10 (3.1.10)
```

```
\pgf@max=\dimen315
\pgf@sys@shading@range@num=\count362
\pgf@shadingcount=\count363
) (c:/texlive/2024/texmf-
dist/tex/generic/pgf/basiclayer/pgfcoreimage.code.tex
File: pgfcoreimage.code.tex 2023-01-15 v3.1.10 (3.1.10)
)
(c:/texlive/2024/texmf-
dist/tex/generic/pgf/basiclayer/pgfcoreexternal.code.tex
File: pgfcoreexternal.code.tex 2023-01-15 v3.1.10 (3.1.10)
\pgfexternal@startupbox=\box129
) (c:/texlive/2024/texmf-
dist/tex/generic/pgf/basiclayer/pgfcorelayers.code.tex
File: pgfcorelayers.code.tex 2023-01-15 v3.1.10 (3.1.10)
)
(c:/texlive/2024/texmf-
dist/tex/generic/pgf/basiclayer/pgfcoretransparency.code
.tex
File: pgfcoretransparency.code.tex 2023-01-15 v3.1.10 (3.1.10)
)
(c:/texlive/2024/texmf-
dist/tex/generic/pgf/basiclayer/pgfcorepatterns.code.tex
File: pgfcorepatterns.code.tex 2023-01-15 v3.1.10 (3.1.10)
) (c:/texlive/2024/texmf-
dist/tex/generic/pgf/basiclayer/pgfcorerdf.code.tex
File: pgfcorerdf.code.tex 2023-01-15 v3.1.10 (3.1.10)
))) (c:/texlive/2024/texmf-
dist/tex/generic/pgf/modules/pgfmoduleshapes.code.te
x
File: pgfmoduleshapes.code.tex 2023-01-15 v3.1.10 (3.1.10)
\pgfnodeparttextbox=\box130
) (c:/texlive/2024/texmf-
dist/tex/generic/pgf/modules/pgfmoduleplot.code.tex
File: pgfmoduleplot.code.tex 2023-01-15 v3.1.10 (3.1.10)
)
(c:/texlive/2024/texmf-dist/tex/latex/pgf/compatibility/pgfcomp-version-
0-65.st
y
Package: pgfcomp-version-0-65 2023-01-15 v3.1.10 (3.1.10)
\pgf@nodesepstart=\dimen316
\pgf@nodesepend=\dimen317
)
(c:/texlive/2024/texmf-dist/tex/latex/pgf/compatibility/pgfcomp-version-
1-18.st
y
Package: pgfcomp-version-1-18 2023-01-15 v3.1.10 (3.1.10)
)) (c:/texlive/2024/texmf-dist/tex/latex/pgf/utilities/pgffor.sty
(c:/texlive/2
024/texmf-dist/tex/latex/pgf/utilities/pgfkeys.sty
(c:/texlive/2024/texmf-dist/
tex/generic/pgf/utilities/pgfkeys.code.tex)) (c:/texlive/2024/texmf-
dist/tex/la
tex/pgf/math/pgfmath.sty (c:/texlive/2024/texmf-
dist/tex/generic/pgf/math/pgfma
```

```
th.code.tex)) (c:/texlive/2024/texmf-
dist/tex/generic/pgf/utilities/pgffor.code
.tex
Package: pgffor 2023-01-15 v3.1.10 (3.1.10)
\pgffor@iter=\dimen318
\pgffor@skip=\dimen319
\pgffor@stack=\toks51
\pgffor@toks=\toks52
)) (c:/texlive/2024/texmf-
dist/tex/generic/pgf/frontendlayer/tikz/tikz.code.tex
Package: tikz 2023-01-15 v3.1.10 (3.1.10)

(c:/texlive/2024/texmf-
dist/tex/generic/pgf/libraries/pgflibraryplothandlers.co
de.tex
File: pgflibraryplothandlers.code.tex 2023-01-15 v3.1.10 (3.1.10)
\pgf@plot@mark@count=\count364
\pgfplotmarksize=\dimen320
)
\tikz@lastx=\dimen321
\tikz@lasty=\dimen322
\tikz@lastxsaved=\dimen323
\tikz@lastysaved=\dimen324
\tikz@lastmovetox=\dimen325
\tikz@lastmovetoy=\dimen326
\tikzleveldistance=\dimen327
\tikzsiblingdistance=\dimen328
\tikz@figbox=\box131
\tikz@figbox@bg=\box132
\tikz@tempbox=\box133
\tikz@tempbox@bg=\box134
\tikztreelevel=\count365
\tikznumberofchildren=\count366
\tikznumberofcurrentchild=\count367
\tikz@fig@count=\count368
(c:/texlive/2024/texmf-
dist/tex/generic/pgf/modules/pgfmodulematrix.code.tex
File: pgfmodulematrix.code.tex 2023-01-15 v3.1.10 (3.1.10)
\pgfmatrixcurrentrow=\count369
\pgfmatrixcurrentcolumn=\count370
\pgf@matrix@numberofcolumns=\count371
)
\tikz@expandcount=\count372

(c:/texlive/2024/texmf-
dist/tex/generic/pgf/frontendlayer/tikz/libraries/tikzli
brarytopaths.code.tex
File: tikzlibrarytopaths.code.tex 2023-01-15 v3.1.10 (3.1.10)
)))
(c:/texlive/2024/texmf-
dist/tex/generic/pgf/frontendlayer/tikz/libraries/tikzli
brarysvg.path.code.tex
File: tikzlibrarysvg.path.code.tex 2023-01-15 v3.1.10 (3.1.10)
```

```
(c:/texlive/2024/texmf-
dist/tex/generic/pgf/libraries/pgflibrarysvg.path.code.t
ex
File: pgflibrarysvg.path.code.tex 2023-01-15 v3.1.10 (3.1.10)
(c:/texlive/2024/texmf-
dist/tex/generic/pgf/modules/pgfmoduleparser.code.tex
File: pgfmoduleparser.code.tex 2023-01-15 v3.1.10 (3.1.10)
\pgfparserdef@arg@count=\count373
)
\pgf@lib@svg@last@x=\dimen329
\pgf@lib@svg@last@y=\dimen330
\pgf@lib@svg@last@c@x=\dimen331
\pgf@lib@svg@last@c@y=\dimen332
\pgf@lib@svg@count=\count374
\pgf@lib@svg@max@num=\count375
))
\@curXheight=\skip166
)

! LaTeX Error: Option clash for package hyperref.

See the LaTeX manual or LaTeX Companion for explanation.
Type  H <return>  for immediate help.
 ...

l.87 \begin{document}

The package hyperref has already been loaded with options:
  []
There has now been an attempt to load it with options
  [colorlinks,allcolors=black,urlcolor=blue]
Adding the global options:
  ,colorlinks,allcolors=black,urlcolor=blue
to your \documentclass declaration may fix this.
Try typing  <return>  to proceed.

Package translations Info: No language package found. I am going to use
`englis
h' as default language. on input line 87.
LaTeX Font Info:    Trying to load font information for T1+Merriwthr-OsF
on inp
ut line 87.
(c:/texlive/2024/texmf-dist/tex/latex/merriweather/T1Merriwthr-OsF.fd
File: T1Merriwthr-OsF.fd 2020/08/30 (autoinst) Font definitions for
T1/Merriwth
r-OsF.
)
LaTeX Font Info:    Font shape `T1/Merriwthr-OsF/m/n' will be
(Font)              scaled to size 7.5pt on input line 87.
(./main.aux)
\openout1 = `main.aux'.

LaTeX Font Info:    Checking defaults for OML/cmm/m/it on input line 87.
LaTeX Font Info:    ... okay on input line 87.
```

```
LaTeX Font Info:    Checking defaults for OMS/cmsy/m/n on input line 87.
LaTeX Font Info:    ... okay on input line 87.
LaTeX Font Info:    Checking defaults for OT1/cmr/m/n on input line 87.
LaTeX Font Info:    ... okay on input line 87.
LaTeX Font Info:    Checking defaults for T1/cmr/m/n on input line 87.
LaTeX Font Info:    ... okay on input line 87.
LaTeX Font Info:    Checking defaults for TS1/cmr/m/n on input line 87.
LaTeX Font Info:    ... okay on input line 87.
LaTeX Font Info:    Checking defaults for OMX/cmex/m/n on input line 87.
LaTeX Font Info:    ... okay on input line 87.
LaTeX Font Info:    Checking defaults for U/cmr/m/n on input line 87.
LaTeX Font Info:    ... okay on input line 87.
LaTeX Font Info:    Checking defaults for PD1/pdf/m/n on input line 87.
LaTeX Font Info:    ... okay on input line 87.
LaTeX Font Info:    Checking defaults for PU/pdf/m/n on input line 87.
LaTeX Font Info:    ... okay on input line 87.
LaTeX Info: Redefining \microtypecontext on input line 87.
Package microtype Info: Applying patch `item' on input line 87.
Package microtype Info: Applying patch `toc' on input line 87.
Package microtype Info: Applying patch `eqnum' on input line 87.
Package microtype Info: Applying patch `footnote' on input line 87.
Package microtype Info: Applying patch `verbatim' on input line 87.
Package microtype Info: Generating PDF output.
Package microtype Info: Character protrusion enabled (level 2).
Package microtype Info: Using default protrusion set `alltext'.
Package microtype Info: Automatic font expansion enabled (level 2),
(microtype)             stretch: 20, shrink: 20, step: 1, non-selected.
Package microtype Info: Using default expansion set `alltext-nott'.
LaTeX Info: Redefining \showhyphens on input line 87.
Package microtype Info: No adjustment of tracking.
Package microtype Info: No adjustment of interword spacing.
Package microtype Info: No adjustment of character kerning.
Package microtype Info: Loading generic protrusion settings for font
family
(microtype)             `Merriwthr-OsF' (encoding: T1).
(microtype)             For optimal results, create family-specific
settings.
(microtype)             See the microtype manual for details.
LaTeX Font Info:    Redeclaring symbol font `operators' on input line 87.
LaTeX Font Info:    Encoding `OT1' has changed to `T1' for symbol font
(Font)              `operators' in the math version `normal' on input
line 87.
LaTeX Font Info:    Overwriting symbol font `operators' in version
`normal'
(Font)                  OT1/cmr/m/n --> T1/Merriwthr-OsF/m/up on input
line 87.

LaTeX Font Info:    Encoding `OT1' has changed to `T1' for symbol font
(Font)              `operators' in the math version `bold' on input line
87.
LaTeX Font Info:    Overwriting symbol font `operators' in version `bold'
(Font)                  OT1/cmr/bx/n --> T1/Merriwthr-OsF/m/up on input
line 87
.
```

```
LaTeX Font Info:    Overwriting symbol font `operators' in version `bold'
(Font)                  T1/Merriwthr-OsF/m/up --> T1/Merriwthr-OsF/b/up
on inpu
t line 87.
LaTeX Font Info:    Redeclaring math alphabet \mathbf on input line 87.
LaTeX Font Info:    Overwriting math alphabet `\mathbf' in version
`normal'
(Font)                  OT1/cmr/bx/n --> T1/Merriwthr-OsF/b/up on input
line 87
.
LaTeX Font Info:    Overwriting math alphabet `\mathbf' in version `bold'
(Font)                  OT1/cmr/bx/n --> T1/Merriwthr-OsF/b/up on input
line 87
.
LaTeX Font Info:    Redeclaring math alphabet \mathsf on input line 87.
LaTeX Font Info:    Overwriting math alphabet `\mathsf' in version
`normal'
(Font)                  OT1/cmss/m/n --> T1/MerriwthrSans-OsF/m/up on
input lin
e 87.
LaTeX Font Info:    Overwriting math alphabet `\mathsf' in version `bold'
(Font)                  OT1/cmss/bx/n --> T1/MerriwthrSans-OsF/m/up on
input li
ne 87.
LaTeX Font Info:    Redeclaring math alphabet \mathit on input line 87.
LaTeX Font Info:    Overwriting math alphabet `\mathit' in version
`normal'
(Font)                  OT1/cmr/m/it --> T1/Merriwthr-OsF/m/it on input
line 87
.
LaTeX Font Info:    Overwriting math alphabet `\mathit' in version `bold'
(Font)                  OT1/cmr/bx/it --> T1/Merriwthr-OsF/m/it on input
line 8
7.
LaTeX Font Info:    Redeclaring math alphabet \mathtt on input line 87.
LaTeX Font Info:    Overwriting math alphabet `\mathtt' in version
`normal'
(Font)                  OT1/cmtt/m/n --> T1/lmtt/m/up on input line 87.
LaTeX Font Info:    Overwriting math alphabet `\mathtt' in version `bold'
(Font)                  OT1/cmtt/m/n --> T1/lmtt/m/up on input line 87.
LaTeX Font Info:    Overwriting math alphabet `\mathsf' in version `bold'
(Font)                  T1/MerriwthrSans-OsF/m/up --> T1/MerriwthrSans-
OsF/b/up
 on input line 87.
LaTeX Font Info:    Overwriting math alphabet `\mathit' in version `bold'
(Font)                  T1/Merriwthr-OsF/m/it --> T1/Merriwthr-OsF/b/it
on inpu
t line 87.
\c@mv@tabular=\count376
\c@mv@boldtabular=\count377
(c:/texlive/2024/texmf-dist/tex/context/base/mkii/supp-pdf.mkii
[Loading MPS to PDF converter (version 2006.09.02).]
\scratchcounter=\count378
\scratchdimen=\dimen333
```

```
\scratchbox=\box135
\nofMPsegments=\count379
\nofMParguments=\count380
\everyMPshowfont=\toks53
\MPscratchCnt=\count381
\MPscratchDim=\dimen334
\MPnumerator=\count382
\makeMPintoPDFobject=\count383
\everyMPtoPDFconversion=\toks54
) (c:/texlive/2024/texmf-dist/tex/latex/epstopdf-pkg/epstopdf-base.sty
Package: epstopdf-base 2020-01-24 v2.11 Base part for package epstopdf
Package epstopdf-base Info: Redefining graphics rule for `.eps' on input line 4
85.
(c:/texlive/2024/texmf-dist/tex/latex/latexconfig/epstopdf-sys.cfg
File: epstopdf-sys.cfg 2010/07/13 v1.3 Configuration of (r)epstopdf for TeX Liv
e
))
*geometry* driver: auto-detecting
*geometry* detected driver: pdftex
*geometry* verbose mode - [ preamble ] result:
* driver: pdftex
* paper: a4paper
* layout: <same size as paper>
* layoutoffset:(h,v)=(0.0pt,0.0pt)
* modes: includefoot twoside
* h-part:(L,W,R)=(54.64pt, 488.22787pt, 54.64pt)
* v-part:(T,H,B)=(66.0pt, 745.04684pt, 34.0pt)
* \paperwidth=597.50787pt
* \paperheight=845.04684pt
* \textwidth=488.22787pt
* \textheight=715.04684pt
* \oddsidemargin=-17.62999pt
* \evensidemargin=-17.62999pt
* \topmargin=-47.76999pt
* \headheight=17.5pt
* \headsep=24.0pt
* \topskip=10.0pt
* \footskip=30.0pt
* \marginparwidth=48.0pt
* \marginparsep=10.0pt
* \columnsep=18.0pt
* \skip\footins=22.0pt plus 2.0pt
* \hoffset=0.0pt
* \voffset=0.0pt
* \mag=1000
* \@twocolumntrue
* \@twosidetrue
* \@mparswitchtrue
* \@reversemarginfalse
* (1in=72.27pt=25.4mm, 1cm=28.453pt)

Package caption Info: Begin \AtBeginDocument code.
```

```
Package caption Info: hyperref package is loaded.
Package caption Info: listings package is loaded.
Package caption Info: End \AtBeginDocument code.

(c:/texlive/2024/texmf-dist/tex/latex/translations/translations-basic-dictionar
y-english.trsl
File: translations-basic-dictionary-english.trsl (english translation file `tra
nslations-basic-dictionary')
)
Package translations Info: loading dictionary `translations-basic-dictionary' f
or `english'. on input line 87.
\c@lstlisting=\count384
Package hyperref Info: Link coloring OFF on input line 87.
(./main.out) (./main.out)
\@outlinefile=\write4
\openout4 = `main.out'.

\@gscitedetails=\box136
\@gscitedetailsheight=\skip167
\@gsheadbox=\box137
\@gsheadboxheight=\skip168
LaTeX Font Info:    Font shape `T1/Merriwthr-OsF/b/n' will be
(Font)              scaled to size 6.5pt on input line 87.
LaTeX Font Info:    Calculating math sizes for size <7.5> on input line 87.

LaTeX Font Warning: Font shape `T1/Merriwthr-OsF/m/up' undefined
(Font)              using `T1/Merriwthr-OsF/m/n' instead on input line 87.

LaTeX Font Info:    Font shape `T1/Merriwthr-OsF/m/up' will be
(Font)              scaled to size 6.24973pt on input line 87.
LaTeX Font Info:    Font shape `T1/Merriwthr-OsF/m/up' will be
(Font)              scaled to size 5.24997pt on input line 87.
LaTeX Font Info:    Trying to load font information for U+eur on input line 87.

(c:/texlive/2024/texmf-dist/tex/latex/amsfonts/ueur.fd
File: ueur.fd 2013/01/14 v3.01 Euler Roman
) (c:/texlive/2024/texmf-dist/tex/latex/microtype/mt-eur.cfg
File: mt-eur.cfg 2006/07/31 v1.1 microtype config. file: AMS Euler Roman (RS)
)

LaTeX Font Warning: Font shape `OMS/cmsy/m/n' in size <7.5> not available
(Font)              size <7> substituted on input line 87.

LaTeX Font Info:    External font `cmex10' loaded for size
(Font)              <7.5> on input line 87.
LaTeX Font Info:    External font `cmex10' loaded for size
(Font)              <6.24973> on input line 87.
```

```
LaTeX Font Info:    External font `cmex10' loaded for size
(Font)              <5.24997> on input line 87.
LaTeX Font Info:    Trying to load font information for U+euf on input
line 87.

(c:/texlive/2024/texmf-dist/tex/latex/amsfonts/ueuf.fd
File: ueuf.fd 2013/01/14 v3.01 Euler Fraktur
) (c:/texlive/2024/texmf-dist/tex/latex/microtype/mt-euf.cfg
File: mt-euf.cfg 2006/07/03 v1.1 microtype config. file: AMS Euler
Fraktur (RS)

)
LaTeX Font Info:    Trying to load font information for U+eus on input
line 87.

(c:/texlive/2024/texmf-dist/tex/latex/amsfonts/ueus.fd
File: ueus.fd 2013/01/14 v3.01 Euler Script
) (c:/texlive/2024/texmf-dist/tex/latex/microtype/mt-eus.cfg
File: mt-eus.cfg 2006/07/28 v1.2 microtype config. file: AMS Euler Script
(RS)
)
LaTeX Font Info:    Trying to load font information for U+euex on input
line 87
.
(c:/texlive/2024/texmf-dist/tex/latex/amsfonts/ueuex.fd
File: ueuex.fd 2013/01/14 v3.01 Euler extra symbols
)

LaTeX Font Warning: Font shape `OML/cmm/m/it' in size <7.5> not available
(Font)              size <7> substituted on input line 87.

LaTeX Font Info:    Font shape `T1/Merriwthr-OsF/m/n' will be
(Font)              scaled to size 6.24973pt on input line 87.
LaTeX Font Info:    Font shape `T1/Merriwthr-OsF/m/n' will be
(Font)              scaled to size 5.24997pt on input line 87.
LaTeX Font Info:    Font shape `T1/Merriwthr-OsF/m/it' will be
(Font)              scaled to size 7.5pt on input line 87.
LaTeX Font Info:    Font shape `T1/Merriwthr-OsF/m/it' will be
(Font)              scaled to size 6.24973pt on input line 87.
LaTeX Font Info:    Font shape `T1/Merriwthr-OsF/m/it' will be
(Font)              scaled to size 5.24997pt on input line 87.
LaTeX Font Info:    Font shape `T1/Merriwthr-OsF/m/n' will be
(Font)              scaled to size 8.0pt on input line 87.
LaTeX Font Info:    Font shape `T1/Merriwthr-OsF/m/it' will be
(Font)              scaled to size 8.0pt on input line 87.
LaTeX Font Info:    Font shape `T1/Merriwthr-OsF/b/it' will be
(Font)              scaled to size 8.0pt on input line 87.
LaTeX Font Info:    Font shape `T1/Merriwthr-OsF/b/n' will be
(Font)              scaled to size 8.0pt on input line 87.

LaTeX Warning: Reference `LastPage' on page 1 undefined on input line 87.

TextBlockOrigin set to 4pc+6.64pt x 4pc+6pt
```

```
Overfull \hbox (54.64pt too wide) in paragraph at lines 100--100
[][]
 []

LaTeX Font Info:    Font shape `T1/Merriwthr-OsF/m/n' will be
(Font)              scaled to size 14.0pt on input line 100.
LaTeX Font Info:    Font shape `T1/Merriwthr-OsF/m/n' will be
(Font)              scaled to size 8.99997pt on input line 100.
LaTeX Font Info:    Calculating math sizes for size <14> on input line
100.
LaTeX Font Info:    Font shape `T1/Merriwthr-OsF/m/up' will be
(Font)              scaled to size 14.0pt on input line 100.
LaTeX Font Info:    Font shape `T1/Merriwthr-OsF/m/up' will be
(Font)              scaled to size 11.66617pt on input line 100.
LaTeX Font Info:    Font shape `T1/Merriwthr-OsF/m/up' will be
(Font)              scaled to size 9.79996pt on input line 100.
LaTeX Font Info:    External font `cmex10' loaded for size
(Font)              <14> on input line 100.
LaTeX Font Info:    External font `cmex10' loaded for size
(Font)              <11.66617> on input line 100.
LaTeX Font Info:    External font `cmex10' loaded for size
(Font)              <9.79996> on input line 100.
LaTeX Font Info:    Font shape `T1/Merriwthr-OsF/m/n' will be
(Font)              scaled to size 11.66617pt on input line 100.
LaTeX Font Info:    Font shape `T1/Merriwthr-OsF/m/n' will be
(Font)              scaled to size 9.79996pt on input line 100.
LaTeX Font Info:    Font shape `T1/Merriwthr-OsF/m/it' will be
(Font)              scaled to size 14.0pt on input line 100.
LaTeX Font Info:    Font shape `T1/Merriwthr-OsF/m/it' will be
(Font)              scaled to size 11.66617pt on input line 100.
LaTeX Font Info:    Font shape `T1/Merriwthr-OsF/m/it' will be
(Font)              scaled to size 9.79996pt on input line 100.
LaTeX Font Info:    Font shape `T1/Merriwthr-OsF/b/n' will be
(Font)              scaled to size 18.0pt on input line 100.
LaTeX Font Info:    Font shape `T1/Merriwthr-OsF/m/n' will be
(Font)              scaled to size 13.0pt on input line 100.
LaTeX Font Info:    Calculating math sizes for size <13> on input line
100.
LaTeX Font Info:    Font shape `T1/Merriwthr-OsF/m/up' will be
(Font)              scaled to size 13.0pt on input line 100.
LaTeX Font Info:    Font shape `T1/Merriwthr-OsF/m/up' will be
(Font)              scaled to size 10.83287pt on input line 100.
LaTeX Font Info:    Font shape `T1/Merriwthr-OsF/m/up' will be
(Font)              scaled to size 9.09996pt on input line 100.

LaTeX Font Warning: Font shape `OMS/cmsy/m/n' in size <13> not available
(Font)              size <12> substituted on input line 100.

LaTeX Font Info:    External font `cmex10' loaded for size
(Font)              <13> on input line 100.
LaTeX Font Info:    External font `cmex10' loaded for size
(Font)              <10.83287> on input line 100.
LaTeX Font Info:    External font `cmex10' loaded for size
(Font)              <9.09996> on input line 100.
```

```
LaTeX Font Warning: Font shape `OML/cmm/m/it' in size <13> not available
(Font)              size <12> substituted on input line 100.

LaTeX Font Info:    Font shape `T1/Merriwthr-OsF/m/n' will be
(Font)              scaled to size 10.83287pt on input line 100.
LaTeX Font Info:    Font shape `T1/Merriwthr-OsF/m/n' will be
(Font)              scaled to size 9.09996pt on input line 100.
LaTeX Font Info:    Font shape `T1/Merriwthr-OsF/m/it' will be
(Font)              scaled to size 13.0pt on input line 100.
LaTeX Font Info:    Font shape `T1/Merriwthr-OsF/m/it' will be
(Font)              scaled to size 10.83287pt on input line 100.
LaTeX Font Info:    Font shape `T1/Merriwthr-OsF/m/it' will be
(Font)              scaled to size 9.09996pt on input line 100.
LaTeX Font Info:    Trying to load font information for TS1+Merriwthr-OsF
on in
put line 100.
(c:/texlive/2024/texmf-dist/tex/latex/merriweather/TS1Merriwthr-OsF.fd
File: TS1Merriwthr-OsF.fd 2020/08/30 (autoinst) Font definitions for
TS1/Merriw
thr-OsF.
)
LaTeX Font Info:    Font shape `TS1/Merriwthr-OsF/m/n' will be
(Font)              scaled to size 10.83287pt on input line 100.
Package microtype Info: Loading generic protrusion settings for font
family
(microtype)             `Merriwthr-OsF' (encoding: TS1).
(microtype)             For optimal results, create family-specific
settings.
(microtype)             See the microtype manual for details.
LaTeX Font Info:    Font shape `T1/Merriwthr-OsF/m/n' will be
(Font)              scaled to size 9.0pt on input line 100.
LaTeX Font Info:    Font shape `T1/Merriwthr-OsF/m/up' will be
(Font)              scaled to size 9.0pt on input line 100.
LaTeX Font Info:    Font shape `T1/Merriwthr-OsF/m/up' will be
(Font)              scaled to size 7.0pt on input line 100.
LaTeX Font Info:    Font shape `T1/Merriwthr-OsF/m/up' will be
(Font)              scaled to size 5.0pt on input line 100.
LaTeX Font Info:    External font `cmex10' loaded for size
(Font)              <9> on input line 100.
LaTeX Font Info:    External font `cmex10' loaded for size
(Font)              <7> on input line 100.
LaTeX Font Info:    External font `cmex10' loaded for size
(Font)              <5> on input line 100.
LaTeX Font Info:    Font shape `T1/Merriwthr-OsF/m/n' will be
(Font)              scaled to size 7.0pt on input line 100.
LaTeX Font Info:    Font shape `T1/Merriwthr-OsF/m/n' will be
(Font)              scaled to size 5.0pt on input line 100.
LaTeX Font Info:    Font shape `T1/Merriwthr-OsF/m/it' will be
(Font)              scaled to size 9.0pt on input line 100.
LaTeX Font Info:    Font shape `T1/Merriwthr-OsF/m/it' will be
(Font)              scaled to size 7.0pt on input line 100.
LaTeX Font Info:    Font shape `T1/Merriwthr-OsF/m/it' will be
(Font)              scaled to size 5.0pt on input line 100.
```

```
LaTeX Font Info:    Font shape `T1/Merriwthr-OsF/m/n' will be
(Font)              scaled to size 6.5pt on input line 100.
LaTeX Font Info:    Calculating math sizes for size <6.5> on input line
100.
LaTeX Font Info:    Font shape `T1/Merriwthr-OsF/m/up' will be
(Font)              scaled to size 6.5pt on input line 100.
LaTeX Font Info:    Font shape `T1/Merriwthr-OsF/m/up' will be
(Font)              scaled to size 5.41643pt on input line 100.
LaTeX Font Info:    Font shape `T1/Merriwthr-OsF/m/up' will be
(Font)              scaled to size 4.54997pt on input line 100.

LaTeX Font Warning: Font shape `OMS/cmsy/m/n' in size <6.5> not available
(Font)              size <6> substituted on input line 100.


LaTeX Font Warning: Font shape `OMS/cmsy/m/n' in size <5.41643> not
available
(Font)              size <5> substituted on input line 100.


LaTeX Font Warning: Font shape `OMS/cmsy/m/n' in size <4.54997> not
available
(Font)              size <5> substituted on input line 100.

LaTeX Font Info:    External font `cmex10' loaded for size
(Font)              <6.5> on input line 100.
LaTeX Font Info:    External font `cmex10' loaded for size
(Font)              <5.41643> on input line 100.
LaTeX Font Info:    External font `cmex10' loaded for size
(Font)              <4.54997> on input line 100.

LaTeX Font Warning: Font shape `OML/cmm/m/it' in size <6.5> not available
(Font)              size <6> substituted on input line 100.


LaTeX Font Warning: Font shape `OML/cmm/m/it' in size <5.41643> not
available
(Font)              size <5> substituted on input line 100.


LaTeX Font Warning: Font shape `OML/cmm/m/it' in size <4.54997> not
available
(Font)              size <5> substituted on input line 100.

LaTeX Font Info:    Font shape `T1/Merriwthr-OsF/m/n' will be
(Font)              scaled to size 5.41643pt on input line 100.
LaTeX Font Info:    Font shape `T1/Merriwthr-OsF/m/n' will be
(Font)              scaled to size 4.54997pt on input line 100.
LaTeX Font Info:    Font shape `T1/Merriwthr-OsF/m/it' will be
(Font)              scaled to size 6.5pt on input line 100.
LaTeX Font Info:    Font shape `T1/Merriwthr-OsF/m/it' will be
(Font)              scaled to size 5.41643pt on input line 100.
LaTeX Font Info:    Font shape `T1/Merriwthr-OsF/m/it' will be
(Font)              scaled to size 4.54997pt on input line 100.
```

```
LaTeX Font Info:    Font shape `TS1/Merriwthr-OsF/m/n' will be
(Font)              scaled to size 5.41643pt on input line 100.

Overfull \hbox (54.64pt too wide) in paragraph at lines 100--100
[][][]
 []

LaTeX Font Info:    Font shape `T1/Merriwthr-OsF/b/n' will be
(Font)              scaled to size 10.0pt on input line 100.

Overfull \hbox (54.64pt too wide) in paragraph at lines 100--100
[][][]
 []

LaTeX Font Info:    Font shape `T1/Merriwthr-OsF/b/n' will be
(Font)              scaled to size 7.5pt on input line 105.

Package natbib Warning: Citation `Climer2021-ch' on page 1 undefined on
input l
ine 105.


Package natbib Warning: Citation `Koutrouli2020-ak' on page 1 undefined
on inpu
t line 105.


Package natbib Warning: Citation `Camacho2018-qm' on page 1 undefined on
input
line 105.




Underfull \vbox (badness 10000) has occurred while \output is active []



LaTeX Font Info:    Font shape `T1/Merriwthr-OsF/m/n' will be
(Font)              scaled to size 7.8pt on input line 106.
LaTeX Font Info:    Font shape `T1/Merriwthr-OsF/b/n' will be
(Font)              scaled to size 7.8pt on input line 106.
[1{c:/texlive/2024/texmf-
var/fonts/map/pdftex/updmap/pdftex.map}{c:/texlive/202
4/texmf-
dist/fonts/enc/dvips/merriweather/merriwthr_posqbl.enc}{c:/texlive/2024
/texmf-dist/fonts/enc/dvips/merriweather/merriwthr_owzwzj.enc}


]

Package natbib Warning: Citation `Langfelder2008-sz' on page 2 undefined
on inp
ut line 107.
```

```
Package natbib Warning: Citation `Cliff2019-sp' on page 2 undefined on
input li
ne 107.


Package natbib Warning: Citation `Angelin-Bonnet2019-vo' on page 2
undefined on
 input line 109.


Package natbib Warning: Citation `Picard2021-xe' on page 2 undefined on
input l
ine 113.


Package natbib Warning: Citation `Furches2019-fy' on page 2 undefined on
input
line 113.


! LaTeX Error: File `figures/multiplexes.tex' not found.

Type X to quit or <RETURN> to proceed,
or enter new name. (Default extension: tex)

Enter file name:
! Emergency stop.
<read *>

l.115 \input{figures/multiplexes}
                                 ^^M
*** (cannot \read from terminal in nonstop modes)


Here is how much of TeX's memory you used:
 35592 strings out of 473583
 713289 string characters out of 5732343
 1984908 words of memory out of 5000000
 57563 multiletter control sequences out of 15000+600000
 1634059 words of font info for 341 fonts, out of 8000000 for 9000
 1141 hyphenation exceptions out of 8191
 123i,11n,131p,1398b,946s stack positions out of
10000i,1000n,20000p,200000b,200000s
!  ==> Fatal error occurred, no output PDF file produced!
```

supplemental

Click here to access/download;LaTeX - Main Document (TeX file);supplemental.tex

```
This is pdfTeX, Version 3.141592653-2.6-1.40.26 (TeX Live 2024)
(preloaded format=pdflatex 2024.8.2)  2 MAR 2025 20:34
entering extended mode
 restricted \write18 enabled.
 %&-line parsing enabled.
**supplemental.tex
(./supplemental.tex
LaTeX2e <2024-06-01> patch level 2
L3 programming layer <2024-05-27>

! LaTeX Error: No counter 'figure' defined.

See the LaTeX manual or LaTeX Companion for explanation.
Type  H <return>  for immediate help.
 ...

l.1 \setcounter{figure}{0}

Your command was ignored.
Type  I <command> <return>  to replace it with another command,
or  <return>  to continue without it.


! LaTeX Error: No counter 'table' defined.

See the LaTeX manual or LaTeX Companion for explanation.
Type  H <return>  for immediate help.
 ...

l.2 \setcounter{table}{0}

Your command was ignored.
Type  I <command> <return>  to replace it with another command,
or  <return>  to continue without it.

! Undefined control sequence.
l.4 \section
            *{Supplemental Material}
The control sequence at the end of the top line
of your error message was never \def'ed. If you have
misspelled it (e.g., `\hobx'), type `I' and the correct
spelling (e.g., `I\hbox'). Otherwise just continue,
and I'll forget about whatever was undefined.


! LaTeX Error: Missing \begin{document}.

See the LaTeX manual or LaTeX Companion for explanation.
Type  H <return>  for immediate help.
 ...

l.4 \section*
             {Supplemental Material}
You're in trouble here.  Try typing  <return>  to proceed.
```

```
If that doesn't work, type  X <return>  to quit.

Missing character: There is no * in font nullfont!
Missing character: There is no S in font nullfont!
Missing character: There is no u in font nullfont!
Missing character: There is no p in font nullfont!
Missing character: There is no p in font nullfont!
Missing character: There is no l in font nullfont!
Missing character: There is no e in font nullfont!
Missing character: There is no m in font nullfont!
Missing character: There is no e in font nullfont!
Missing character: There is no n in font nullfont!
Missing character: There is no t in font nullfont!
Missing character: There is no a in font nullfont!
Missing character: There is no l in font nullfont!
Missing character: There is no M in font nullfont!
Missing character: There is no a in font nullfont!
Missing character: There is no t in font nullfont!
Missing character: There is no e in font nullfont!
Missing character: There is no r in font nullfont!
Missing character: There is no i in font nullfont!
Missing character: There is no a in font nullfont!
Missing character: There is no l in font nullfont!

Overfull \hbox (20.0pt too wide) in paragraph at lines 4--5
[]
 []

! Undefined control sequence.
l.6 \subsection
               {Supplemental Figures}
The control sequence at the end of the top line
of your error message was never \def'ed. If you have
misspelled it (e.g., `\hobx'), type `I' and the correct
spelling (e.g., `I\hbox'). Otherwise just continue,
and I'll forget about whatever was undefined.


! LaTeX Error: Missing \begin{document}.

See the LaTeX manual or LaTeX Companion for explanation.
Type  H <return>  for immediate help.
 ...

l.6 \subsection{S
                 upplemental Figures}
You're in trouble here.  Try typing  <return>  to proceed.
If that doesn't work, type  X <return>  to quit.

Missing character: There is no S in font nullfont!
Missing character: There is no u in font nullfont!
Missing character: There is no p in font nullfont!
Missing character: There is no p in font nullfont!
Missing character: There is no l in font nullfont!
```

```
Missing character: There is no e in font nullfont!
Missing character: There is no m in font nullfont!
Missing character: There is no e in font nullfont!
Missing character: There is no n in font nullfont!
Missing character: There is no t in font nullfont!
Missing character: There is no a in font nullfont!
Missing character: There is no l in font nullfont!
Missing character: There is no F in font nullfont!
Missing character: There is no i in font nullfont!
Missing character: There is no g in font nullfont!
Missing character: There is no u in font nullfont!
Missing character: There is no r in font nullfont!
Missing character: There is no e in font nullfont!
Missing character: There is no s in font nullfont!
! Undefined control sequence.
l.7 \FloatBarrier

The control sequence at the end of the top line
of your error message was never \def'ed. If you have
misspelled it (e.g., `\hobx'), type `I' and the correct
spelling (e.g., `I\hbox'). Otherwise just continue,
and I'll forget about whatever was undefined.


! LaTeX Error: File `supplemental/figures/cv_differences.tex' not found.

Type X to quit or <RETURN> to proceed,
or enter new name. (Default extension: tex)

Enter file name:
! Emergency stop.
<read *>

l.8 \input{supplemental/figures/cv_differences}
                                               ^^M
*** (cannot \read from terminal in nonstop modes)


Here is how much of TeX's memory you used:
 16 strings out of 473583
 446 string characters out of 5732343
 1925908 words of memory out of 5000000
 23009 multiletter control sequences out of 15000+600000
 558069 words of font info for 36 fonts, out of 8000000 for 9000
 1141 hyphenation exceptions out of 8191
 18i,0n,26p,111b,18s stack positions out of
10000i,1000n,20000p,200000b,200000s
!  ==> Fatal error occurred, no output PDF file produced!
```

Manuscript
Click here to access/download;Manuscript;RWRtoolkit.pdf

(GIGA)^n SCIENCE

GigaScience, 2023, 1–13

doi: xx.xxxx/xxxx
Manuscript in Preparation
Paper

PAPER

RWRtoolkit: multi-omic network analysis using random walks on multiplex networks in any species

David Kainer [1,2,*], Matthew Lane [1,3,*], Kyle A. Sullivan [1], J. Izaak Miller [1], Mikaela Cashman [1,4], Mallory Morgan [1], Ashley Cliff [1,3], Jonathon Romero [1,3], Angelica Walker [1,3], D. Dakota Blair [5], Hari Chhetri [1], Yongqin Wang [5], Mirko Pavicic [1], Anna Furches [1,3], Jaclyn Noshay [1], Meghan Drake [1], AJ Ireland [6], Ali Missaoui [5], Yun Kang [7,8], John C. Sedbrook [9], Paramvir Dehal [6], Shane Canon [6] and Daniel Jacobson [1 †]

[1]Computational and Predictive Biology Group, Oak Ridge National Laboratory, Oak Ridge, TN, USA and [2]Centre of Excellence for Plant Success in Nature and Agriculture, University of Queensland, QLD, Australia and [3]The Bredesen Center for Interdisciplinary Research and Graduate Education, University of Tennessee Knoxville, Knoxville, TN, USA and [4]Environmental Genomics and Systems Biology Division, Lawrence Berkeley National Laboratory Berkeley, California, USA and [5]Computational Science Initiative, Brookhaven National Laboratory, Upton, NY, USA and [6]Department of Crop and Soil Sciences, University of Georgia, Athens, GA, USA and [7]Noble Research Institute, Ardmore, OK, USA and [8]Driscoll's Inc., Watsonville, CA, USA and [9]School of Biological Sciences, Illinois State University, Normal, IL, United States

[*]Contributed equally.
[†]Corresponding Author: jacobsonda@ornl.gov

Abstract

We introduce RWRtoolkit, a multiplex generation, exploration, and statistical package built for R and command line users. RWRtoolkit enables the efficient exploration of large and highly complex biological networks generated from custom experimental data and/or from publicly available datasets, and is species agnostic. A range of functions can be used to find topological distances between biological entities, determine relationships within sets of interest, search for topological context around sets of interest, and statistically evaluate the strength of relationships within and between sets. The command-line interface is designed for parallelisation on high performance cluster systems, which enables high throughput analysis such as permutation testing. Several tools in the package have also been made available for use in reproducible workflows via the KBase web application.

Key words: Multiplex network; Systems biology; Random Walk with Restart; Multi-Omic; Software Package

Background

Biological studies are increasingly pursuing and obtaining data on larger scales and at multiple levels in the molecular hierarchy of the study system. One approach for dealing with the multiplicity of data in modern biology is to represent the relationships in the data as a network [1]. Each entity in a dataset (e.g., each gene) becomes a node, and an edge between two nodes represents a relationship that has been measured or predicted between those nodes (e.g.,

Compiled on: February 28, 2025.
Draft manuscript prepared by the author.

their co-expression in a population, sharing of common protein domains, similarity of methylation state, etc.). Once in network form, a great variety of network analysis methods become available [2, 3]. Genes that are strongly connected to each other are topologically more likely to be functionally relevant to each other than more distal or loosely connected genes in the network. Machine learning algorithms can be used to efficiently explore entire networks and find such relationships with respect to a set of starting genes, often called seeds or anchors. This approach is particularly useful for exploring the functional context around sets of genes, such as those produced from Genome Wide Association Studies (GWAS), Quantitative Trait Loci (QTL) mapping, differential expression analysis or case/control proteomics.

To analyze a set of genes in a network context, a network and an algorithm to traverse that network are required. The underlying network may be as simple as a single-layer of nodes and edges generated from one experimental dataset that predicts relationships between genes, such as co/predictive expression relationship determined from RNA-seq results [4, 5].

There exist many types of relationships in biological systems as many types of measurements exist, such as proteomic assays, bulk and single-cell RNAseq transcription, metabolomic profiling, and more. These different omics "layers" offer distinct insights into the mechanisms within that layer of questioning. Relationships within biological systems, however, are not isolated within the layer in which their data were measured. The internal mechanisms of the cell interact not only with similar layer elements, but also these mechanisms are heavily involved in inter-layer interaction as well [6].

Combining these differing omics layers for downstream analysis can be a difficult process. Commonly, the multiple layers of input networks are aggregated into a single layer by summing or averaging multiple edges between the same pair of nodes into one composite edge. The result is a single adjacency matrix representation of the data, also known as a monoplex network. The aggregated network is a summarization of the input layers, and as such has lost the unique topological information carried by each layer.

A more robust approach to combining these heterogeneous data layers is to incorporate these differing lines of evidence into a multigraph, which are networks that can contain multiple edges between two vertices. [7]. A multigraph network potentially fills in relationship gaps that exist in any given single layer and enables simultaneous exploration of multi-omic data since one can traverse the network from node to node using edges from all layers. When relationships between specific nodes are present in multiple layers, a multi-layer network presents multiple lines-of-evidence (LOE) that those genes are functionally related [8].

Multiplex networks are types of multigraphs that are structured such that, for all possible types of connections, there exists a corresponding layer for those types of edges to exist and connections between all nodes of the same kind between layers [9]. This multiplex structure is achieved through the incorporation of individual layers' adjacency matrices along the diagonal of the "supra-adjacency" matrix, with inter-layer edges connecting corresponding nodes across layers (**Fig. 1**) . Multiplex network representations of multiomic systems have been demonstrated to outperform other methods of network aggregation [10, 11, 12, 13]. Though the predictive capability of the multiplex networks does exceed that of monoplex networks, the supra-adjacency matrix is more difficult to build and adds an extra level of complexity to network exploration algorithms.

A wide variety of algorithms exist for ranking genes according to their topological connectivity to the seed (candidate) genes in the underlying network. One simple approach, known as neighbor voting [14], scores each gene by counting their outgoing edges that directly connect to the seeds. However, by only looking at immediate connections to the seeds, the influence and importance of genes farther away is ignored. Other traditional algorithms for exploring biological networks include calculating clustering coefficients,

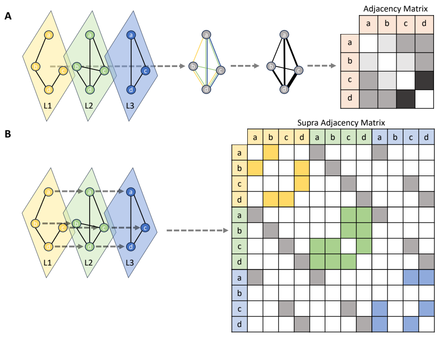

Figure 1. Aggregated monoplex network vs Multiplex network. In this example there are three small input network layers (L1, L2, L3), with a union set of nodes of size n=4, with which to generate a multi-layer network. **A.** In the aggregated approach the layers are merged into one. If multiple edges occur between any pair of nodes, their weights are aggregated to produce the final adjacency matrix of size n x n. **B.** In the multiplex approach each layer is kept separate via a supra-adjacency matrix of size (n x L) X (n x L) where L is the number of layers. Nodes that are common across layers are connected by virtual edges (gray arrows). The diagonal blocks of the supra-adjacency matrix represent the standard adjacency matrices within each individual layer. Connectivity between layers is represented in the off-diagonal blocks, with virtual edges coloured in gray. Note that layer L3 (blue) does not contain node 'b', so there are no inter-layer virtual edges from L1-L3 or L2-L3 for node 'b'.

centrality metrics and network density [15]. More advanced propagation approaches, such as diffusion and random walk with restart (RWR) use the entire network topology to score and rank every node, and have been shown to be generally superior in their ability to find true positive relationships [16, 17, 18]. A Random Walk can be described conceptually as a "walker" which proceeds to wander outwards from a starting seed gene, choosing which edge to take with a probability equal to 1/d where d is the degree of the current gene (d is the out-degree for directed networks). Over multiple iterations, the walker explores the network in this manner so the proportion of time spent at each gene forms a probability distribution that represents how accessible every gene in the network is when starting from one or more seed gene(s). With RWR, at each iteration the walker teleports back to the starting point with restart probability r to prevent the walker from wandering too far in the global topology, or getting stuck in various topological structures.

The R package RandomWalkRestartMH provides the essential functionality for implementing RWR on multiplex networks, but it focuses primarily on executing the algorithm itself [13]. Therefore it lacks many of the higher-level features often needed in sophisticated biological analyses, leaving researchers to piece together additional tools for tasks such as multiplex network construction, network validation, parallelized permutation testing, and neighborhood analysis. The use of the RandomWalkRestartMH package additionally assumes a passing knowledge of the R environment for users.

Here we introduce RWRtoolkit [19], an R package that builds on RandomWalkRestartMH and fills these gaps through functionalities with a corresponding set of command line tools designed for ease of use, enabling the easy construction of multiplex networks from any set of data layers, followed by analysis of candidate gene sets within the networks using the Random Walk with Restart (RWR) algorithm. We have additionally integrated RWRtoolkit with a graphical user interface (GUI) into the Department of Energy's (DOE) Knowledge Base (KBase) system [20].

RWRtoolkit extends and updates the RandomWalkRestartMH R package [13], which provides the core functionality to generate multiplex networks from a set of input network layers, and implements the Random Walk Restart algorithm on a supra-adjacency matrix. Once a multiplex network has been generated, the RWRtoolkit provides commands to rank all genes in the overall network according

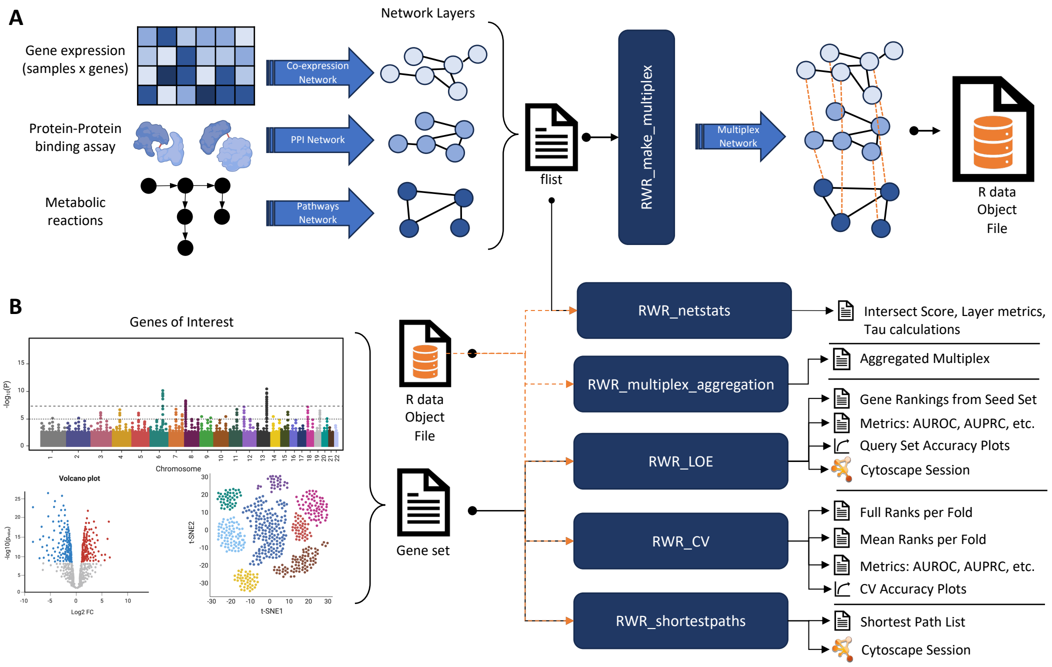

Figure 2. A general workflow for using the RWRtoolkit. A. Illustration of how a user can generate several network layers from different omics data sources, which become input to the RWRtoolkit workflow. Once the user has networks in the correct format, they can then refer to them via a flist file and use RWR_make_multiplex to turn them into a homogeneous multiplex network (e.g., multiple layers of gene-to-gene relationships). This multiplex is wrapped in an RData object that is saved for future use. **B.** A demonstration of how the user can now execute a variety of multi-omic analyses, most of which require the RData object as input. A set of genes of interest (gene set) from discovery studies such as GWAS or differential expression analysis can be used as input to multiple tools. These tools output a variety of files that show how functionally connected the genes in the gene set are to each other, or to a second gene set of interest, or to all the other genes in the multiplex. Some resulting networks can be automatically visualized in Cytoscape via the RCy3 R package [23]. Figure uses illustrations created with BioRender.com.

to their connectivity to a set of seed genes, use cross-validation to assess the network's predictive ability or determine the topological similarity of a set of genes, and find shortest paths between sets of seed genes. The RWRtoolkit R package outputs detailed tables of ranked genes as well as statistics of predictive accuracy (AUROC, AUPRC, etc.), plots, and network visualizations of the multi-omic neighborhood around the seed genes. Furthermore, RWRtoolkit commands can be run from the command-line interface, which enables high throughput parallelized analysis (such as permutation testing) on compute clusters.

To date, multi-omic networks have been made publicly available in a range of model species: AraNet, PopGenie, StringDB, YeastNet. However these networks are often aggregated into a single layer rather than multiplexed, and it is difficult or impossible for the user to customize their choice of input layers or include custom layers generated from their own experimental results or algorithms. RWRtoolkit enables network analysis of multi-omic data for any species, allowing researchers to use their own datasets and networks and/or pre-existing networks. We demonstrate this by generating a custom Arabidopsis thaliana multiplex network and using it to analyze gene sets from a novel GWAS study and a published gene knockout study [21] (see **Supplemental Case Studies**). RWRtoolkit is available with installation instructions, user guide, and sample data at http://github.com/dkainer/RWRtoolkit [22].

Data Description

The RWRtoolkit codebase provides functions for multiplex network generation, running random walks starting from given seed sets, validation functions for seed sets as well as network layers, and general multiplex network statistics. These functions are made available via a cross-platform R package with command-line interface commands and a web GUI. We have provided multiple tutorials in the R vignette format for users to explore at their own leisure. Our methods were generated with gene relationships as a focus, but these random walk methods can be applicable to any data type in network format. Example methods can be seen in Table 1 and example input and output can be seen in **Supplemental Tables 1-18**.

We created the RWRtoolkit R package as a collection of functions designed with ease of use in mind, particularly for users who are not familiar with the R environment as all input values for the functions take file paths as input (**Fig. 2**). We have additionally implemented updates to the RandomWalkRestartMH package.

RandomWalkRestartMH Updates

For broad installation purposes, we have removed the DNET package from our implementation of the package, as it is no longer supported by CRAN. This ensures ongoing compatibility with CRAN and helps maintain the stability of our software. In our updated RandomWalkRestartMH codebase, the code relies on parallel computing constructs rather than a straightforward sequential approach. Instead of iterating through multiplex network layers in nested loops, multiple workers are spawned, and updates to the layers within the supra-adjacency matrix are implemented in parallel. This shifts the logic flow by segmenting operations across concurrent processes, which can speed up large computations. The added parallel step does introduce new system demands, notably requiring additional libraries and configuration for managing distributed tasks. Despite this overhead, the updated code can handle large multiplex networks more effectively. In addition, the previous iteration of the RandomWalkRestartMH package suggested that the parameter Tau exerted little influence on the final output. We found that the original codebase implementations for calculating

Table 1. RWRtoolkit function calls from either an R environment or command line.

Command	R	Shell
Make Multiplex	RWR_make_multiplex(<br>flist='./example_data/flist.txt'<br>)	Rscript ./scripts/run_make_multiplex.R \\<br>--flist ./example_data/flist.txt
Netstats	RWR_netstats(<br>data = './example_data/string_interactions.Rdata',<br>outdir = './netstats_output',<br>network_1 =<br>'./example_data/netstat/combined_score-random-gold.tsv',<br>network_2 =<br>'./example_data/netstat/combined_score-random-test.tsv',<br>basic_statistics = T,<br>scoring_metric = "both",<br>pairwise_between_mpo_layer = T,<br>multiplex_layers_to_refnet = T,<br>net_to_net_similarity = T,<br>calculate_tau_for_mpo = T ,<br>calculate_exclusivity_for_mpo = T<br>)	Rscript ./scripts/run_netstats.R \\<br>--data ./example_data/string_interactions.Rdata \\<br>--outdir ./netstats_output \\<br>--network_1 \\<br>./example_data/netstat/combined_score-random-gold.tsv \\<br>--network_2 \\<br>./example_data/netstat/combined_score-random-test.tsv \\<br>--basic_statistics \\<br>--pairwise_between_mpo_layer \\<br>--multiplex_layers_to_refnet \\<br>--net_to_net_similarity \\<br>--calculate_tau_for_mpo \\<br>--calculate_exclusivity_for_mpo \\<br>--verbose
Network Aggregation	RWR_network_aggregation(<br>data = './example_data/string_interactions.Rdata',<br>outdir = './netstats_networks',<br>merged_with_all_edges = T,<br>merged_with_edgecounts = T<br>)	Rscript ./scripts/run_network_aggregation.R \\<br>--data ./example_data/string_interactions.Rdata \\<br>--outdir ./netstats_networks \\<br>--merged_with_all_edges \\<br>--merged_with_edgecounts
LOE	RWR_LOE(<br>data = './example_data/string_interactions.Rdata',<br>seed_geneset= './example_data/geneset1.tsv',<br>outdir = './loe_output_dir'<br>)	Rscript ./scripts/run_loe.R \\<br>--data ./example_data/string_interactions.Rdata \\<br>--seed_geneset ./example_data/geneset1.tsv \\<br>--outdir ./loe_output_dir
CV	RWR_CV(<br>data = './example_data/string_interactions.Rdata',<br>geneset_path= './example_data/geneset1.tsv',<br>method='kfold',<br>folds=3,<br>outdir = './cv_kfold_output_dir'<br>)	Rscript ./scripts/run_cv.R \\<br>--data ./example_data/string_interactions.Rdata \\<br>--geneset ./example_data/geneset1.tsv \\<br>--method kfold \\<br>--folds 3 \\<br>--outdir ./cv_kfold_output_dir
Shortest Paths	RWR_ShortestPaths(<br>data = './example_data/string_interactions.Rdata',<br>source_geneset= './example_data/geneset1.tsv',<br>target_geneset='./example_data/geneset2.tsv',<br>outdir='./shortest_paths_output'<br>)	Rscript ./scripts/run_shortestpaths.R \\<br>--data ./example_data/string_interactions.Rdata \\<br>--source_geneset ./example_data/geneset1.tsv \\<br>--target_geneset ./example_data/geneset2.tsv \\<br>--outdir ./shortest_paths_output

Tau across the layers contained an error and have implemented a fix. Our findings indicate adjusting Tau can indeed affect the multiplex network's overall performance, resulting in notable changes to output scores and rank ordering.

Multiplex Generation

RWRtoolkit workflows typically start with the RWR_make_multiplex command, which handles the creation of the multiplex network. It requires a descriptor file (known as an "flist") that lists the full path to each network layer to be included in the multiplex. Each network layer's file must be formatted as a simple delimited edge list with a column for the source genes, a column for the target genes, and an optional weight column (see **Supplemental Tables 1**, **2**, and **3** for file examples). This function combines the creation of the multiplex object, the supra-adjacency matrix, as well as the normalized supra-adjacency matrix. The generation of the multiplex object and supra-adjacency matrix use updated RandomWalkRestartMH functionality that parallelizes the multiplex object creation and supra-adjacency matrix generation. This updated methodology employs a task mapping strategy to directly assign values of 1 to the corresponding i, j edges across all network layers in the supra-adjacency matrix, in contrast to the previous approach that embedded complete adjacency matrices along the diagonal of the supra-adjacency matrix.

The generated multiplex network is automatically saved as an Rdata object containing the individual layers as igraph [24, 25] networks, the multiplex supra-adjacency matrix, transition matrix, and network metadata. This Rdata object is used as input for most downstream commands.

Multiplex RWR Applications

Network Layer and Multiplex Statistics

The contents of a multiplex network affect the outcomes of RWR analyses. The RWR_netstats command lets the user evaluate individual network layers or the contents of an entire multiplex network containing many layers. Basic statistics (basic_statistics) for individual layers can be calculated, as well as more complex relationships such as jaccard or overlap scores for inter-layer similarities (pairwise_between_mpo_layer), multiplex layer to reference network (multiplex_layers_to_refnet), and single network to network similarities (net_to_net_similarity). Additionally, the RWR tau parameter affects the probability of the walker visiting each specific layer, allowing the user to bias the walk to certain layers of higher

importance. Users can supply their own tau values, or use the calculate_tau function to return a tau value for each layer based on each layer's overlap with a gold-standard network.

Evaluating Multiplex Networks and Gene Sets Using Cross Validation

The predictive ability of a multiplex network can be determined using cross validation of gold standard or reference gene sets with the RWR_CV command. A gold standard gene set typically contains genes that are known to be functionally related (e.g., all are members of one biosynthetic pathway, all are annotated with the same GO/KEGG term, etc.). The hypothesis is that gold standard genes purposely left out from the seed set should be found with relatively high precision (i.e., highly ranked by RWR) if the underlying networks are indeed functionally predictive. The RWR_CV command allows the user to provide a gold standard gene set and use k-fold, leave-one-out, or singleton cross validation to score the ability to find the left out gene(s).

RWR_CV generates output files that include the RWR score and rank of each gene in the multiplex (as detailed by RWR_LOE for each fold), the mean rank of each gene across all folds, evaluation metrics based on the ranks of seed genes for each fold, and a comprehensive evaluation summary file. Users can select from three methods to test gene sets against networks: KFold, where each seed gene N is ranked K-1 times across K folds, yielding K-1 RWR scores per gene (**Supplemental Figure 1 A**); Leave One Out (LOO), where each seed gene N is ranked across N-1 folds, with one gene left out from the seed set per fold; and Singletons, where each seed gene N is individually ranked N-1 times across N folds, with only one single seed gene per fold. Metric calculations for determining precision and recall with respect to ranked data per fold are derived from Järvelin and Kekäläinen [26]. A more in depth discussion of metric calculation can be found in the supplemental material. File descriptions and examples can be found in **Supplemental Tables 14-16**.

Ranking Genes Using Multiple Lines of Evidence (LOE)

The RWR_LOE command uses RWR to rank all genes in the multiplex network with respect to a gene-set of interest (seed genes), which provides multi-omic biological context for the seeds. The ranks and scores of all genes can be output to a file. A second gene set can be provided in order to evaluate the topological relationship between two sets of genes. When a second gene set is provided, those genes are flagged within the ranked output. The network context around the top N ranked genes can be easily visualized via an integrated connection to Cytoscape [27] using the RCy3 [23] R package with the –cyto=N flag.

Extracting the Shortest Paths Across the Multiplex Between Genes

In a network there can exist many unique paths between two particular nodes. Obtaining the shortest paths between any two given nodes within a network can provide crucial insight to a network's topology or the relationship between those nodes. RWR_ShortestPaths calculates the pairwise shortest paths between source and target gene sets, and returns them as a series of edges that form the shortest path, the layers in which those edges exist, edge weights, and normalized edge weights. Shortest paths between seed and target sets can additionally be automatically visualized in Cytoscape using the –cyto flag.

Network Aggregation Functions

Two methods of network aggregation are provided to merge the layers of a multiplex network into a single monoplex network. The merged_with_all_layers function aggregates all layers maintaining multiple edges between nodes. The merged_with_all_edgecounts function aggregates all layers of the multiplex, but instead edge weight is calculated as the sum of all shared edges within the multiplex network.

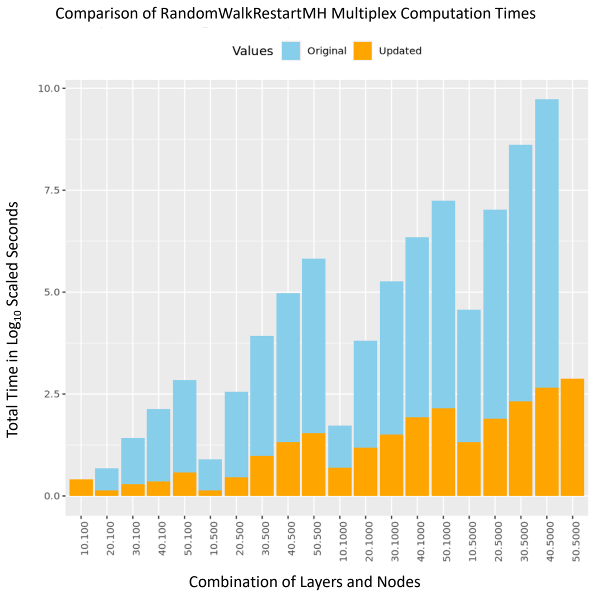

Figure 3. Multiplex Generation Timing: Comparing the compute time for two separate implementations of the generation of a multiplex object, supra-adjacency matrix, and transition matrix in log scale. The generation of multiplex networks in the original implementation (blue) takes significantly longer to process compared to its updated counterpart (orange). The speedup is due to the parallel processing of edge updating within the supra-adjacency matrix across 32 cores.

Results

RandomWalkRestartMH Multiplex Generation Updates

Comparing the multiplex generation methods for creating the multiplex network object, generating the supra-adjacency matrix, and the transition matrix of the previous iteration of RandomWalkRestartMH and the current, parallelized version, our parallelized method for calculating multiplex networks at scale surpasses the previous implementation of multiplex generation. **Figure 3** demonstrates that as node count as well as layer count increase, the amount of time required for multiplex generation increases accordingly. Our updated implementation significantly improves performance, particularly for larger networks with greater numbers of nodes and layers. It is particularly noteworthy that the network of 50 layers timed out after 6 hours of construction whereas the updated parallelized method constructed the network in 17.69 seconds.

RandomWalkRestartMH Tau Updates

Testing with a two-layer multiplex illustrates that the original implementation of get.seed.scoresMultiplex produced incorrect seed weight adjustments, while the updated version reflects seed weights weighted by layer (i.e. higher scores for layer 1, lower scores for layer 2) (**Fig. 4B**). Similarly, the revised Random.Walk.Restart.Multiplex function behaves as expected when the tau parameter decreases, causing proportional reductions in non-seed nodes (C and D) in layer 2. In contrast the original implementation shows no changes in these scores as tau decreases (**Fig. 4C**).

RWRtoolkit Applications

Multiplex Construction

We constructed our comprehensive Arabidopsis multiplex with a total of 9 layers ranging from 789 to 19,975 nodes. The full mul-

tiplex took 59 seconds to construct and contains 26605 unique nodes and 918,640 edges (see Data availability for multiplex links). We used the RWR_netstats function to calculate basic statistics and pairwise jaccard comparison between all layers in the multiplex, producing 2 output files: base_stats.tsv and pairwise_between_mpo_layer_jaccard.tsv are illustrated in **Table 2** and **Figure 5**, respectively.

Table 2. RWR Netstats Basic statistics for the Comprehensive Arabidopsis thaliana Multiplex.

Network Name	Number of Nodes	Number of Edges	Diameter
DU	2283	13514	13
GA	7683	84959	22
KS	1841	94952	6
PP	19191	317787	16
PX	19975	145407	12
PY	13314	71287	9
RE	789	1359	16
RP	16014	167851	6
RX	2857	21524	19

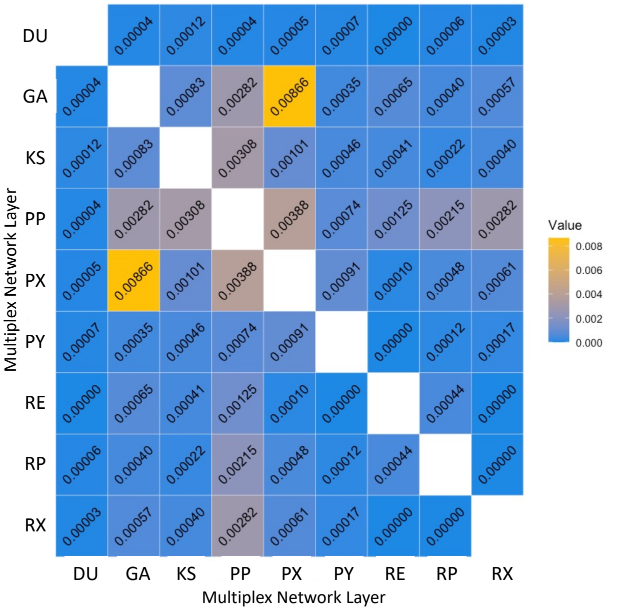

Figure 5. Pairwise Between MPO Layer Jaccard Netstat Jaccard similarity coefficients for the edges of each network with respect to all other networks in the multiplex network. Overall, there is little overlap in edges between network layers, illustrating the heterogeneity of information encoded within each layer. The layers that contain the greatest amount of jaccard similarity are PP and PX, Protein Protein Interaction and Predictive Expression.

Network Validation

We ran RWR_CV using k-fold (k=5) on each of 25 MAPMAN-derived [28] gene sets to validate the predictive ability of the multiplex network for gene function. This resulted in an average AUROC of 0.91 across all gene sets and CV folds, indicating a strong overall ability to find the left-out genes from a functional group and rank them highly. When we performed the same analysis on 1000 randomly rewired multiplexes using command-line RWR_CV, the overall average AUROC was 0.49 (where 0.50 is considered the equivalent of random). A comparison of AUROC densities for individual MAPMAN gene sets are illustrated in **Figures 6A** and **6B** and the aggregate of all MAPMAN gene set AUROC densities is illustrated in **Figure 6C**. Individual RWR_CV comparison statistics for each MAPMAN gene set can be found in **Table 3**.

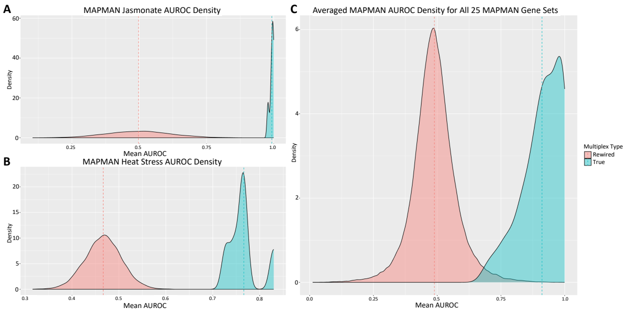

Figure 6. A comparison of the mean AUROC scores from the kfold output of RWR_CV using the true Comprehensive Multiplex (blue) and 1000 randomly rewired multiplex networks (red). A. Illustration of a comparison of AUROC density across 5 folds using an individual set of genes curated for Jasmonate signaling obtained from MAPMAN. The true comprehensive multiplex (blue) has a mean AUROC across 5 folds of 0.993 whereas the 1000 rewired multiplexes have an average mean AUROC across 5 folds of 0.498. **B.** Depiction of a comparison of AUROC density across 5 folds using an individual set of genes curated for Heat Stress signaling obtained from MAPMAN. The True comprehensive multiplex has a mean AUROC across 5 folds of 0.766. The average mean AUROC across 5 folds for the 1000 rewired multiplexes is 0.467. **C.** Illustration of a comparison of the average AUROC density across 25 gold standard gene sets generated from shared MAPMAN terms including Jasmonate and Heat Stress signaling. The true Comprehensive Multiplex has an overall average mean AUROC of 0.91 across all 25 gold standard gene sets, whereas the 1000 rewired multiplex networks have an overall average AUROC of 0.489 across all 25 gold standard gene sets, illustrating that the true Comprehensive Multiplex has meaningful biological connections compared to the completely random connections found across the 1000 rewired multiplex networks.

Running a Random Walk on a Multiplex

Using the RWRtoolkit software application to run RWR_LOE, we used AT2G44810 and AT1G17420 as seeds with cyto parameter set

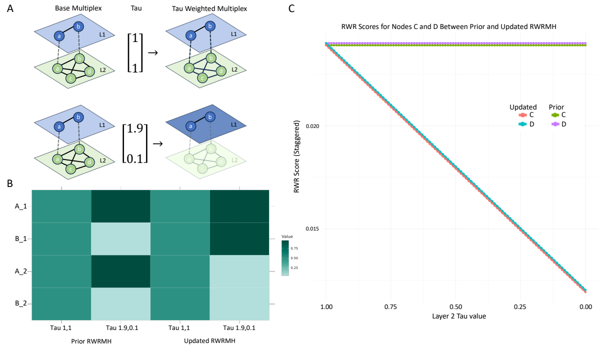

Figure 4. Tau Comparison: **A**. For a multiplex, setting each layer's Tau to a different value should alter the weight of the seed nodes within those layers, causing RWR to be biased towards exploring those layers with higher Tau values. **B**. Comparing the original RandomWalkRestartMH functionality for updating seed vectors with Tau to the updated version illustrates that both versions work properly with respect to equal weighting. When given a Tau to weigh layer 1 with 1.9 and layer 2 with 0.1, the original implementation weighs seed A with 1.9 and seed B with 0.1 across layers. Conversely, the updated implementation weighs the layers with Tau accordingly. **C**. Given random walks with seeds A and B, the RWR scores of nodes C and D do not change as Tau for layer 2 is decreased to 0 with respect to the original implementation. In the updated implementation, as tau for layer 2 decreases, the RWR scores of C and D decrease accordingly.

to 50 to visualize the subnetwork of the top 50 ranked genes and all lines of evidence connecting those genes (**Fig. 7A**). The full score and rankings for all genes in the multiplex were reported to file. The top 50 subnetwork was aggregated and sent to Cytoscape via the RCy3 API with all edges colored within the visualization by layer name. Conversely, the web-based KBase implementation visualized the top 50 selected genes within a separate window with interactive

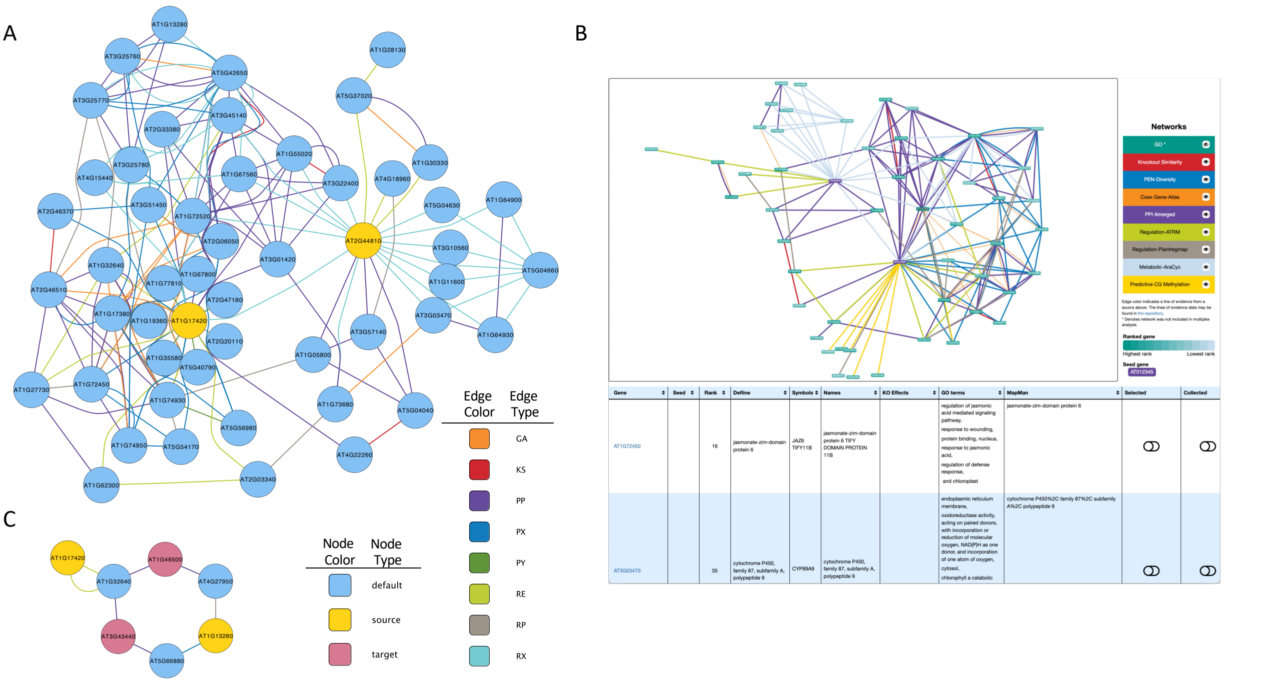

Figure 7. RWRtoolkit Multiplex Visualizations: **A**. Using the RWR_LOE function with the '–cyto' flag, users can specify the total number of nodes they wish to see in the Cytoscape visualization. The total multiplex is aggregated for visualization purposes with all edge types maintained for visual styling. **B**. The RWR_LOE function within the KBase application automatically visualizes the network output, illustrating the layers from which each edge came on the same screen as the RWR_LOE node score output. **C**. RWR_ShortestPaths finds the shortest paths between any two sets of nodes and plots the subnetwork into Cytoscape.

javascript functionality(**Fig. 7B**).

Evaluating the Connectivity of a Set of Genes

Using 5-fold RWR_CV we ranked 30 seed genes (27 MAPMAN Jasmonate genes and 3 from differing ontology terms). Average rank per gene is denoted in **Table 4**. Almost all (23/27) of the jasmonate genes were ranked in the top 100, while the remaining 4 jasmonate genes, AT5G20900 AT3G17860 AT3G43440 and AT1G48500, had relatively high ranks ranks of 128, 246, 1517, 1947, respectively. The 3 non-jasmonate genes, AT4G33360, AT5G23010, AT1G08380, were ranked considerably lower at 2600, 9426, and 9707. The KBase application reports the same files and information.

Extracting the Shortest Paths

Extracting the shortest paths from the source genes AT1G13280 and AT1G17420 and target genes AT1G48500 and AT3G43440, all 4 paths had a length of 3 and are reported in **Table 5**. The cyto flag exports a visualization of all nodes within the shortest paths in cytoscape (**Fig. 7C**).

Discussion

Using the RWRtoolkit package, users can easily create and evaluate multiplex biological networks encoding multiple lines of interaction evidence. Importantly, RWRtoolkit is agnostic to organism, tissue or condition. The user may apply these tools to non-model organisms by using orthologs of available networks from model organisms, or by building custom networks from experimental data as demonstrated here, or a combination of both.

RWRtoolkit uses RWR to explore and score topological connectivity from multiple lines of evidence between seed genes and other genes. In doing so, RWRtoolkit facilitates interpretation of a gene set beyond gene set enrichment analysis. It allows the user to expand the biological context of a gene or gene set using all lines available of evidence jointly. Moreover, the biological context and relationships within a gene set is explainable and quantifiable. Users can further explore the lines of evidence supporting gene-to-gene interactions (e.g., co-expression, protein-protein interactions, etc.) using Cytoscape or the KBase implementation.

RWRtoolkit was designed with ease of use in mind for researchers familiar with the R software environment and those familiar with only command line interfaces. Users who want to generate custom multiplex networks and use the entire suite of functions in RWRtoolkit can find the open-source code and vignettes on GitHub. For users who prefer a point-and-click graphical user interface or have limited bioinformatic experience, we have included RWR_LOE and RWR_CV as applications within KBase and provided pre-assembled Arabidopsis thaliana multiplex networks.

Addressing Limitations in the RandomWalkRestartMH Package

In addition to developing this suite of tools, we have updated the base underlying functionality of RandomWalkRestartMH. While RandomWalkRestartMH provides core functionality for constructing multiplex networks and implementing random walk algorithms, the construction of the multiplex was not optimized. As the size of networks and the total number of layers increases, the computational load for constructing the multiplex increases exponentially. With RWRtoolkit we parallelized this process to dramatically reduce the time to construct a large multiplex network from hours to seconds, thus enabling genome-wide multiplexes in higher organisms with tens of thousands of genes.

With respect to the tau parameter for weighting layers within the multiplex, the implementation in the RandomWalkRestartMH package was not correctly weighting the seeds within the multiplex network, leading Valdeolivas et al. [13] to conclude that tau had a minimal effect on output rankings. In that implementation tau incorrectly affects each seed's weight equally across all layers rather than differently per layer. The impact is demonstrated in **Figure 4B**

where changing tau fails to affect the RWR scores for nodes C and D which are present only in layer 2.

Comparatively, in our updated implementation, as we decrease tau for a given layer, we see a commensurate decrease in score for nodes existing only in that layer, illustrating that the tau parameter can play a measurable role with respect to node scoring. The corrected implementation gives users more flexibility to usefully weight the layers within their multiplexes.

RWRtoolkit

Multiplex Generation and Netstats

The construction time of the multiplex network with 26,605 unique nodes and 918,640 edges demonstrates the computational efficiency and scalability of this parallelized method. This is especially relevant for research using larger, more complex networks.

The netstats functionality allows for users to concisely generate multiplex metadata and statistics and output those data to file. The basic statistics data provides a file output of useful context for interpreting the structural diversity of the layers within the multiplex, while the pairwise between layer jaccard similarity functionality offers a unique look at edge set comparisons. **Figure 5** illustrates that very few edges intersect between layers with respect to any two layers. This illustrates the heterogeneity of information encoded within the multiplex network, indicating that each layer is offering distinct biological aspects not encoded within the other layers.

Evaluation of Multiplex Networks Using MAPMAN Gene Sets

One of the standout capabilities of RWRtoolkit is its ability to evaluate multiplex networks using gene sets known to have known biological connections and related topology, such as genes with shared annotation from GO or MAPMAN. These manually curated genes represent functionally cohesive sets derived from independent experimentation and databases, and provide a framework for testing a multiplex network's predictive capabilities. The example

Table 4. RWR CV Leave One Out cross validation to explore how well connected each node is with respect to all remaining nodes within the seed set of interest.

NodeNames	meanrank	rerank
AT1G13280	1	1
AT1G17420	1	1
AT1G72520	1	1
AT3G25760	1	1
AT5G42650	1	1
AT2G06050	2	6
AT3G22400	2	6
AT3G25770	2	6
AT3G25780	2	6
AT3G45140	2	6
AT1G17990	3	11
AT1G18020	3	11
AT1G55020	3	11
AT1G67560	5	14
AT1G17380	7	16
AT1G72450	8	17
AT2G44810	9	18
AT1G74950	15	23
AT1G09400	23	28
AT1G76690	26	29
AT1G76680	27	30
AT1G19180	29	31
AT1G70700	45	44
AT5G20900	277	128
AT3G17860	519	246
AT3G43440	2063	1517
AT1G48500	2607	1947
AT4G33360	3280	2600
AT5G23010	9382	9425
AT1G08380	9644	9707

Table 3. Mean and Standard Deviation of K-Fold AUROC Scores for Comprehensive and Rewired Multiplexes.

Geneset	Rewired 1000 Average AUROC	Rewired 1000 std AUROC	Comprehensive net average AUROC	Comprehensive net std AUROC
Abscisic acid	4.99E-01	7.73E-02	8.71E-01	6.95E-02
Auxin	4.81E-01	4.30E-02	8.42E-01	3.98E-02
Brassinosteroids	4.89E-01	9.98E-02	9.39E-01	4.64E-02
Cell Wall Synthesis	5.01E-01	5.55E-02	9.82E-01	1.27E-02
CHO metabolism	4.97E-01	4.08E-02	9.39E-01	3.07E-02
Coldstress	5.01E-01	1.75E-01	9.81E-01	1.87E-02
Cytokinin	4.95E-01	1.27E-01	9.06E-01	7.55E-02
Drought salt	4.98E-01	8.28E-02	8.69E-01	3.91E-02
Ethylene	4.80E-01	6.08E-02	8.77E-01	4.81E-02
Ethylene and EREBP	4.93E-01	4.05E-02	9.25E-01	7.18E-03
Fatty Acid	4.91E-01	5.46E-02	9.32E-01	4.25E-02
Flavonoids	5.02E-01	7.09E-02	9.48E-01	2.88E-02
Gibberelin	4.78E-01	9.54E-02	8.50E-01	9.14E-02
Glucosinolates	4.99E-01	7.31E-02	9.39E-01	3.61E-02
Heat stress	4.67E-01	3.90E-02	7.66E-01	3.48E-02
HeatshockTFs	4.66E-01	3.86E-02	7.69E-01	5.85E-02
Isoprenoids	4.99E-01	5.62E-02	9.85E-01	1.51E-02
Jasmonate	4.98E-01	1.21E-01	9.93E-01	7.94E-03
Lignin Biosynthesis	5.03E-01	1.10E-01	9.99E-01	3.11E-04
Major CHO	4.90E-01	5.87E-02	9.14E-01	4.16E-02
Phenylpropanoids	5.01E-01	7.70E-02	9.97E-01	2.33E-03
PS Light Reaction	4.53E-01	4.63E-02	8.41E-01	3.27E-02
PS Light Reaction II	4.58E-01	7.55E-02	9.02E-01	1.36E-02
Salicylic Acid	5.01E-01	1.50E-01	8.99E-01	1.08E-01

This table contains the mean values and standard deviation of AUROC for RWR_CV Kfold cross validation for 25 curated gene sets which all share the same MAPMAN term. The average and standard deviation values for the rewired networks are averages and standard deviations across 1000 iterations of the RWR_CV Kfold Cross Validation in which the edges of each network were rewired for each iteration. Conversely, the average and standard deviation values for the Comprehensive network alone are those pertaining only to the average values across the single RWR_CV Kfold Cross Validation (k=5).

Table 5. RWR Shortest Paths from the source AT1G13280 and AT1G17420 to the targets AT1G48500 and AT3G43440.

From	To	Weight	Type	Weight Norm	Path Name	Path Length	Path Elements
AT1G48500	AT4G27950	1	PP	3.15E-06	AT1G13280_AT1G48500	3	AT1G13280→AT4G27950→AT1G48500
AT4G27950	AT1G13280	1	RP	5.96E-06	AT1G13280_AT1G48500	3	AT1G13280→AT4G27950→AT1G48500
AT5G66880	AT3G43440	1	PP	3.15E-06	AT1G13280_AT3G43440	3	AT1G13280→AT5G66880→AT3G43440
AT5G66880	AT1G13280	1	PX	6.88E-06	AT1G13280_AT3G43440	3	AT1G13280→AT5G66880→AT3G43440
AT1G32640	AT1G17420	1	RE	0.00073584	AT1G17420_AT1G48500	3	AT1G17420→AT1G32640→AT1G48500
AT1G32640	AT1G48500	1	PP	3.15E-06	AT1G17420_AT1G48500	3	AT1G17420→AT1G32640→AT1G48500
AT1G32640	AT1G17420	1	RE	0.00073584	AT1G17420_AT3G43440	3	AT1G17420→AT1G32640→AT3G43440
AT1G32640	AT3G43440	1	PP	3.15E-06	AT1G17420_AT3G43440	3	AT1G17420→AT1G32640→AT3G43440

cross-validation experiment conducted on our 9-layer Comprehensive Arabidopsis multiplex demonstrates that functionally related groups of genes have strong topological similarity within this multiplex such that a subset of a functional gene set can be used to reliably predict functionally related genes with few relatively false positives. For comparison, the same analysis on rewired multiplex networks achieve no apparent predictive ability, illustrating that the networks are poorly constructed with respect to Arabidopsis genetic interactions.

Testing the Connectivity of a Set of Genes

It is often the case in biological research that a list of genes will be generated from a statistical test or experiment such as GWAS or differential expression analysis. Using tools such as RWR_CV, we can explore how well these genes rank each other with respect to all others in the set, illustrating how topologically related those genes are given the layers in the multiplex network. Using a set of genes sharing the Jasmonate signalling term from the MAPMAN database, we see a small example of the power of RWR_CV's leave one out (loo) method. Given that there are 30 genes within the set, 30 individual tests of RWR were run, leaving only one gene out each time. By then considering the mean rank and updated (rerank) ranks of all genes in the gene set, we can begin to understand how topologically related all genes in the gene set are to each other.

Twenty seven of the 30 genes were annotated as jasmonate-related, and 23 of those achieved mean ranks in the top 100 demonstrating their close functional roles and topological similarity. Unsurprisingly, the three genes from non-jasmonate MAPMAN terms (AT4G33360, AT5G23010, and AT1G08380) ranked poorly with mean ranks of 2600, 9425, and 9707 since the gene set was dominated by Jasmonate functionality. Such RWR_CV rankings can therefore be used to parse gene sets apart by function or detect false positive genes in a gene set [29]. Two biological case studies using RWR_CV functionality, one using the RWRtoolkit R package installation and the other using the KBase web application GUI can be found as supplemental material.

RWRtoolkit was developed for applications to biological networks, but given that networks are domain agnostic and can signify any entity to entity relationship (including social networks, transportation networks, etc.), RWRtoolkit's algorithms could be applied to any network data to identify highly ranked nodes using random walk with restart.

Visualizing Output

The web of connections within the multiplex network is overwhelming for meaningful interpretation of gene-to-gene relationships and biological context. RWRtoolkit, through commands like RWR_CV, RWR_LOE and shortest_paths, reduce the network down to a context of interest around seed genes and their high ranking connections. The software offers multiple routes for visualizing these subnetsworks via the RCy3 API to Cytoscape or the KBase web app. We see this in **Figure 7A**, where the top 50 genes from the run are plotted into Cytoscape and styled. This immediately allows for visual inspection of the seed genes, their topologically similar genes, and their connecting lines of evidence. For users leveraging the KBase GUI (**Figure 7B**), web-based visualization tools allow for a seamless exploration of the gene scores, rankings, inter and intra-layer connections.

Methods

All RWRtoolkit and RandomWalkRestartMH R software was run on the Andes compute system at the Oak Ridge Leadership Computing Facility (OLCF) at Oak Ridge National Laboratory (ORNL). The computations were run on a single compute node, with 2 AMD EPYC 7302 3 GHz 16-Core processors with 256 GB Memory.

RandomWalkRestartMH Multiplex Generation Updates

We installed two separate instances of RandomWalkRestartMH: the original implementation defined by [13] installed as "RandomWalkRestartMHOriginal", and our updated version. Both implementations of RandomWalkRestartMH define the same core functions for multiplex network processing: create.multiplex, which constructs the multiplex network list of igraph objects; compute.adjacency.matrix, which generates the supra-adjacency matrix for the multiplex; and compute.transition.matrix, which normalizes the adjacency matrix to create a transition matrix. We implemented a custom framework for evaluating the speed of these functions in multiplex generation.

We implemented a grid computation for generating multiplex networks of varying layer count $L \in (10, 20, 30, 40, \text{and } 50)$ and node number per layer $N \in (50, 100, 500, 1000, \text{and } 5000)$.

For each combination (L,N), we generated L random networks of size N using the Erdős-Rényi model in the igraph package [30, 25]. These networks were then used to generate multiplex networks using both the previous implementation of RandomWalkRestartMH as well as our updated implementation. The updated implementation of RandomWalkRestartMH used 32 cores to compute the supra-adjacency matrix while the original implementation is single threaded.

RandomWalkRestartMH Tau Updates

Valdeolivas et al. [13] noted that changing the tau parameter had minimal influence on RWR scores. Our investigation revealed an error within the original codebase so we updated our codebase to enable tau to weight layers appropriately.

To illustrate the impact of correctly operating tau we constructed a toy multiplex network consisting of 2 network layers - one layer a two-node path with nodes A and B and the other layer a clique of 4 nodes A,B,C, and D. We ran 100 iterations of Random.Walk.Restart.Multiplex function from RandomWalkRestartMH with nodes A and B as seeds while varying the values of tau for the second layer from 2 (maximum importance for this layer) to 0 (no importance for this layer).

RWRtoolkit Applications

Multiplex Construction and Layers

For our base application and public distribution, several Arabidopsis thaliana multiplex networks were generated, including a comprehensive network with 9 layers. Network layer descriptions, sources and sizes are provided in **Table 6**. All input networks were converted to be unweighted, and the multiplexes were constructed with a delta value of 0.5, where delta defines the global probability of the random walker transitioning between layers during an RWR iteration.

For the comprehensive multiplex network, we used the RWR_netstats function to extract basic statistics using the option basic_statistics, and to determine the edge-wise overlap between layers using the pairwise_between_layers option and the default jaccard similarity score.

Network Evaluation

To ensure the integrity of the multiplex network construction, we employed Random Walk with Restart Cross-Validation (RWR_CV) using a series of carefully curated gene sets known to exhibit high functional relatedness. These gene sets were based on shared MAPMAN annotations [28] that are curated by experts, and therefore represent functionally cohesive and biologically meaningful gene clusters. In a well-constructed multiplex network these genes are expected to be more topologically similar to each other than random genes would be, reflecting the robustness of the network's structural integrity and its biological accuracy. We performed 5-fold (method="kfold") cross-validation (k=5) for each MAPMAN-derived gene set with a restart value of 0.7 to evaluate the constructed multiplex network. The results from these evaluations were compared to those obtained from the same analysis conducted on 1000 randomly rewired multiplex networks, each preserving the same number of nodes and edges across all layers.

Running a Random Walk on a Multiplex

Users can get the Random Walk scores and ranks of all other genes within a multiplex network with respect to the seed gene of interest. We used the RWR_LOE function with a seed set file containing the seed genes of AT2G44810 and AT1G17420. These genes are part of a gene set with a shared MAPMAN ontology of Jasmonate and were selected arbitrarily for the purposes of illustrating RWR_LOE functionality. The analysis was run with a restart value of 0.7 and cyto as 50 using the RWRtoolkit software package. Using the Kbase application, we loaded the Arabidopsis_thaliana genome in the Data section. The Arabidopsis_thaliana genome was loaded via the public database tab, using the NCBI RefSeq Genomes. We next selected the "Build FeatureSet from Genome" from the Comparative Genomics dropdown from the "Apps" section to add the cell into the Kbase Narrative. In the "Build FeatureSet from Genome" cell, we selected the Arabidopsis_thaliana genome, searched and selected AT2G44810 and AT1G17420 in the Protein-Encoding Gene Feature text box. The output feature set was named and the cell was run. To run the random walk, the RWR_LOE cell was selected from the Comparative Genomics drop down within the Apps window. Within the RWR_LOE Cell, we selected the generated seed gene set from the "Seed Gene Keys" dropdown, selected the "Comprehensive Network" from the multiplex dropdown, chose 50 nodes as the maximum node rank to visualize, and named the output file object.

Testing the Connectivity of a Set of Genes

It is possible that some genes within a gene set are more functionally related than others. For the sake of illustration, we selected a set of 27 genes with the shared MAPMAN ontology term "Jasmonate". We also added genes AT1G08380, AT4G33360, and AT5G23010, from the MAPMAN ontology terms "Photosynthesis light reaction", "Flavonoids" and "Glucosinolates" respectively. We then used RWR_CV to score how connected these 30 genes are

Table 6. A list of all network layers within the Comprehensive Multiplex Network.

Network Layer	Description	Nodes	Edges
CoEvolution-DUO	Gene A connects to Gene B if a SNP in or near Gene A is correlated with a SNP in or near Gene B using the DUO metric. [31]	2283	13514
Coexpression	Gene-Atlas Coexpression network obtained from AtGenie.org. [32]	7683	84959
Knockout Similarity	Gene A connects to Gene B if the phenotypic effect of knocking out GeneA is simila to the phenotypic effect of knocking out GeneB. [33]	1841	94952
PPI-6merged	GeneA connects to GeneB if their protein products have been shown to bind to interact with each other, typically through experimental evidence. The PPI-6merged network is the union of 6 different A.thaliana PPI networks: AraNet2 LC, AraNet2 HT [34], AraPPInet2 0.60 [35], BIOGRID 4.3.194 physical [36], AtPIN [37], and Mentha. [38]	19191	317787
PEN-Diversity	Gene A connects to Gene B if the expression vector of Gene A is an important predictor of the expression vector of Gene B in an iRF model, where all other genes' expression are included as covariates. [5, 39]	19975	145407
Predictive CG Methylation	Gene A connects to Gene B if the CG methylation vector of Gene A is an important predictor of the CG methylation vector of Gene B in an iRF model, where all other genes' CG methylation states are included as covariates. [5, 39]	13314	71287
Regulation-ATRM	Gene A connects to Gene B if Gene A is a Transcription Factor (TF) that is show to interact with Gene B (which may or may not be a TF). This dataset contains literature mined and manually curated TF regulatory interactions for A.thaliana [40]	789	1359
Regulation-Plantregmap	This network contains computationally predicted TF-Target relationships based on motifs, binding sites, ChipSeq data [41]	16014	167851
Metabolic-AraCyc	Gene A connects to Gene B if they are both enzymatic and are linked by a common substrate or product. [42]	2857	21524

to each other within the topology of the comprehensive multiplex network, using a restart value of 0.7 and the loo (leave one out) CV option.

RWR_CV functionality and several pre-generated Arabidopsis multiplexes are also integrated into KBase as a GUI within the Comparative Genomics Apps. To define the gene set in KBase we used the "Build FeatureSet from Genome" option in the Comparative Genomics Apps section, selected the Arabidopsis_thaliana genome, and selected all 30 genes defined above. We then selected "Find Gene Set Interconnectivity using Cross Validation with RWRtoolsCV" from the Comparative Genomics dropdown and the comprehensive multiplex. From advanced parameters, we selected "loo" as the method of choice and chose 30 folds (as there are 30 genes within the input set).

Extracting the Shortest Paths

To see how those gene sets are connected within the multiplex network, all possible shortest paths between all seed and target genes were extracted using the RWRtoolkit application. We selected two genes as source genes AT1G13280 and AT1G17420 and two genes as target genes: AT1G48500 and AT3G43440. We ran RWR_ShortestPaths with the source and target gene sets as parameters.

Availability of source code and requirements

- Project Name: RWRtoolkit
- Project home page: `https://github.com/dkainer/rwrtoolkit`
- Operating system(s): Platform independent
- Programming language: R
- Other requirements: R 4.1.0 or higher
- License: GPL-3.0 license
- Software Heritage PID: swh:1:snp:0f97744911eaa2098a614537ee7249bdcc513b3f

Data availability

The data sets supporting the results of this article are available:

- Well-Watered Shoot Biomass GWAS Results from Supplemental Case Study can be found in Supplemental Table 19.
- KBase Narrative is publicly available at: [43]
- Snapshots of the code are available in Software Heritage [44]
- Snapshots of the Pre-Assembled Arabidopsis Networks and MAPMAN derived gene sets available at GitHub repository [45] and in Software Heritage [46]

Declarations

List of abbreviations

- AIG1: AVIRULENCE INDUCED GENE
- AS2: ASYMMETRIC LEAVES 2
- BLUPS: Best Linear Unbiased Predictors
- BP: Biological Process
- CV: Cross Validation
- EMB3113: EMBRYO DEFECTIVE 3113
- FAD2: FATTY ACID DESATURASE 2
- FAE1: FATTY ACID ELONGATION1
- GO: Gene Ontology
- GWAS: Genome Wide Association Study
- HD-ZIP III: Homeodomain Leucine-Zipper
- JA: Jasmonic Acid.
- KAN1: KANADI1
- LCBs: Long-Chain Bases
- LOE: Lines of Evidence
- PIN1: PIN-FORMED 1
- PHB: PHABULOSA
- PHV: PHAVOLUTA
- PPI: Protein-Protein Interactions
- ROD1: REDUCED OLEATE DESATURATION 1
- RWR: Random Walk with Restart
- SLD1: SPHINGOID LCB DESATURASE 1
- SLD2: SPHINGOID LCB DESATURASE 2
- SNP: Single Nucleotide Polymorphism
- TF: Transcription Factor
- QTL: Quantitative Trait Locus
- WOX9: WUSCHEL-RELATED HOMEOBOX 9

Ethical Approval

Not applicable

Consent for publication

Not applicable

Competing Interests

The authors have no competing interests.

Funding

This work is supported as part of the Genomic Sciences Program DOE Systems Biology Knowledgebase (KBase) funded by the Office of Biological and Environmental Research's Genomic Science program within the US Department of Energy Office of Science under Award Number DE-AC02-05CH11231. This work was also funded by The Center for Bioenergy Innovation (CBI), which is a U.S. Department of Energy Bioenergy Research Center supported by the Office of Biological and Environmental Research in the DOE Office of Science. Support was also provided by the Integrated Pennycress Resilience Project (IPReP), funded by the Office of Biological and Environmental Research's Genomic Science program within the US Department of Energy Office of Science. This research is additionally supported by the U.S. Department of Energy, Office of Science, Office of Biological and Environmental Research, Genomic Science Program grant number DE-SC0021286. Oak Ridge National Laboratory is managed by UT-Battelle, LLC for the US DOE under Contract Number DE-AC05-00OR22725.

Author's Contributions

The individual contributions of authors to the manuscript are specified following the CASRAI CRediT Contributer Roles Taxonomy:

- Conceptualization and Resources: David Kainer and Dan Jacobson
- Data curation: David Kainer, Matthew Lane, Kyle A. Sullivan, J. Izaak Miller, Mikaela Cashman, Yongqin Wang, John Sedbrook
- Formal Analysis: David Kainer, Matthew Lane, Kyle A. Sullivan, Mallory Morgan, Mirko Pavicic
- Funding acquisition: David Kainer, Ali Missaoui, Yun Kang, Dan Jacobson
- Investigation: David Kainer, Matthew Lane, Kyle A. Sullivan, Mallory Morgan
- Methodology: David Kainer, Matthew Lane, Kyle A. Sullivan, Ashley Cliff, Jonathon Romero, Angelica Walker, Hari Chhetri, Anna Furches
- Project administration: Meghan Drake

- Software: David Kainer, Matthew Lane, Kyle A. Sullivan, J. Izaak Miller, Mikaela Cashman, D. Dakota Blair, AJ Ireland
- Supervision: David Kainer, Yun Kang, Paramvir Dehal, Shane Canon
- Validation: Matthew Lane, Kyle A. Sullivan, J. Izaak Miller, Mikaela Cashman
- Visualization: David Kainer, Matthew Lane
- Writing – original draft: David Kainer, Matthew Lane, Kyle A. Sullivan, Mallory Morgan, Mirko Pavicic, Anna Furches
- Writing – review & editing: David Kainer, Matthew Lane, Kyle A. Sullivan, Mallory Morgan, Anna Furches, Meghan Drake

Acknowledgements

This manuscript has been authored by UT-Battelle, LLC under Contract No. DE-AC05-00OR22725 with the U.S. Department of Energy. The United States Government retains and the publisher, by accepting the article for publication, acknowledges that the United States Government retains a non-exclusive, paid-up, irrevocable, world-wide license to publish or reproduce the published form of this manuscript, or allow others to do so, for United States Government purposes. The Department of Energy will provide public access to these results of federally sponsored research in accordance with the DOE Public Access Plan (http://energy.gov/downloads/doe-public-access-plan). This research used resources of the Oak Ridge Leadership Computing Facility at the Oak Ridge National Laboratory, which is supported by the Office of Science of the U.S. Department of Energy under Contract No. DE-AC05-00OR22725. The switchgrass resequencing data were produced by the US Department of Energy Joint Genome Institute (https://ror.org/04xm1d337; operated under Contract No. DE-AC02-05CH11231) in collaboration with the user community. Sujan Mamidi performed sequence alignments and called the SNP variants, and Jeremy Schmutz leads the HudsonAlpha sequencing efforts.

References

1. Climer S. Connecting the dots: The boons and banes of network modeling. Patterns (N Y) 2021 Dec;2(12):100374.
2. Koutrouli M, Karatzas E, Paez-Espino D, Pavlopoulos GA. A Guide to Conquer the Biological Network Era Using Graph Theory. Front Bioeng Biotechnol 2020 Jan;8:34.
3. Camacho DM, Collins KM, Powers RK, Costello JC, Collins JJ. Next-Generation Machine Learning for Biological Networks. Cell 2018 Jun;173(7):1581–1592.
4. Langfelder P, Horvath S. WGCNA: an R package for weighted correlation network analysis. BMC Bioinformatics 2008 Dec;9:559.
5. Cliff A, Romero J, Kainer D, Walker A, Furches A, Jacobson D. A High-Performance Computing Implementation of Iterative Random Forest for the Creation of Predictive Expression Networks. Genes 2019 Dec;10(12).
6. Angelin-Bonnet O, Biggs PJ, Vignes M. Gene regulatory networks: A primer in biological processes and statistical modelling. Methods Mol Biol 2019;1883:347–383.
7. Picard M, Scott-Boyer MP, Bodein A, Périn O, Droit A. Integration strategies of multi-omics data for machine learning analysis. Comput Struct Biotechnol J 2021 Jun;19:3735–3746.
8. Furches A, Kainer D, Weighill D, Large A, Jones P, Walker AM, et al. Finding New Cell Wall Regulatory Genes in Populus trichocarpa Using Multiple Lines of Evidence. Front Plant Sci 2019 Oct;10:1249.
9. Kivela M, Arenas A, Barthelemy M, Gleeson JP, Moreno Y, Porter MA. Multilayer networks. J Complex Netw 2014 Sep;2(3):203–271.
10. Didier G, Brun C, Baudot A. Identifying communities from multiplex biological networks. PeerJ 2015 Dec;3(e1525):e1525.
11. Didier G, Valdeolivas A, Baudot A. Identifying communities from multiplex biological networks by randomized optimization of modularity. F1000Res 2018 Nov;7:1042.
12. Battiston S, Caldarelli G, Garas A. Multiplex and Multilevel Networks. Oxford University Press, USA; 2018.
13. Valdeolivas A, Tichit L, Navarro C, Perrin S, Odelin G, Levy N, et al. Random walk with restart on multiplex and heterogeneous biological networks. Bioinformatics 2019;35(3):497–505.
14. Weighill D, Jones P, Shah M, Ranjan P, Muchero W, Schmutz J, et al. Pleiotropic and Epistatic Network-Based Discovery: Integrated Networks for Target Gene Discovery. Frontiers in Energy Research 2018;6.
15. Erciyes K. Graph-theoretical analysis of biological networks: A survey. Computation (Basel) 2023 Sep;11(10):188.
16. Cowen L, Ideker T, Raphael BJ, Sharan R. Network propagation: a universal amplifier of genetic associations. Nat Rev Genet 2017 Sep;18(9):551–562.
17. Di Nanni N, Bersanelli M, Milanesi L, Mosca E. Network Diffusion Promotes the Integrative Analysis of Multiple Omics. Front Genet 2020 Feb;11:106.
18. Picart-Armada S, Barrett SJ, Willé DR, Perera-Lluna A, Gutteridge A, Dessailly BH. Benchmarking network propagation methods for disease gene identification. PLoS Comput Biol 2019 Sep;15(9):e1007276.
19. DOE CODE: Project Metadata for Code ID 74677;. Accessed: 2024-7-30. https://doi.org/10.11578/dc.20220607.1.
20. Arkin AP, Cottingham RW, Henry CS, Harris NL, Stevens RL, Maslov S, et al. KBase: The United States Department of Energy Systems Biology Knowledgebase. Nat Biotechnol 2018 Jul;36(7):566–569.
21. Jarvis BA, Romsdahl TB, McGinn MG, Nazarenus TJ, Cahoon EB, Chapman KD, et al. CRISPR/Cas9-Induced and Mutations Stacked With Confer High Oleic Acid Seed Oil in Pennycress (L.). Front Plant Sci 2021 Apr;12:652319.
22. Kainer, D, Lane, M, Sullivan, KA, Cashman, M, Miller, JI, dkainer/RWRtoolkit;. Accessed: 2024-1-30. https://doi.org/10.11578/dc.20220607.1.
23. Gustavsen JA, Pai S, Isserlin R, Demchak B, Pico AR. RCy3: Network biology using Cytoscape from within R. F1000Res 2019 Oct;8:1774.
24. Csárdi G, Nepusz T, Horvát S, Traag V, Zanini F, Noom D, igraph. Zenodo; 2024. https://doi.org/10.5281/zenodo.8315832.
25. Csárdi G, Nepusz T, Müller K, Horvát S, Traag V, Zanini F, et al., igraph for R: R interface of the igraph library for graph theory and network analysis. Zenodo; 2024. https://doi.org/10.5281/zenodo.13964143.
26. Järvelin K, Kekäläinen J. IR evaluation methods for retrieving highly relevant documents. SIGIR Forum 2017 Aug;51(2):243–250.
27. Shannon P, Markiel A, Ozier O, Baliga NS, Wang JT, Ramage D, et al. Cytoscape: a software environment for integrated models of biomolecular interaction networks. Genome Res 2003 Nov;13(11):2498–2504.
28. Thimm O, Bläsing O, Gibon Y, Nagel A, Meyer S, Krüger P, et al. MAPMAN: a user-driven tool to display genomics data sets onto diagrams of metabolic pathways and other biological processes. Plant J 2004 Mar;37(6):914–939.
29. Sullivan KA, Lane M, Cashman M, Miller JI, Pavicic M, Walker AM, et al. Analyses of GWAS signal using GRIN identify additional genes contributing to suicidal behavior. Commun Biol 2024 Oct;7(1):1360.
30. Csárdi G, Nepusz T, Traag V, Horvát S, Zanini F, Noom D, et al., Igraph: Network analysis and visualization. The R Foundation; 2006. http://dx.doi.org/10.32614/cran.package.igraph.
31. Climer S, Templeton AR, Garvin M, Jacobson D, Lane M, Hulver S, et al. Synchronized genetic activities in Alzheimer's

brains revealed by heterogeneity-capturing network analysis. bioRxiv 2020 Jan;p. 2020.01.28.923730. https://doi.org/10.1101/2020.01.28.923730.

32. Sundell D, Mannapperuma C, Netotea S, Delhomme N, Lin YC, Sjödin A, et al. The Plant Genome Integrative Explorer Resource: PlantGenIE.org. New Phytol 2015 Dec;208(4):1149–1156.
33. Oellrich A, Walls RL, Cannon EK, Cannon SB, Cooper L, Gardiner J, et al. An ontology approach to comparative phenomics in plants. Plant Methods 2015 Feb;11:10.
34. Lee T, Yang S, Kim E, Ko Y, Hwang S, Shin J, et al. AraNet v2: an improved database of co-functional gene networks for the study of Arabidopsis thaliana and 27 other nonmodel plant species. Nucleic Acids Res 2015 Jan;43(Database issue):D996–1002.
35. Zhang F, Liu S, Li L, Zuo K, Zhao L, Zhang L. Genome-Wide Inference of Protein-Protein Interaction Networks Identifies Crosstalk in Abscisic Acid Signaling. Plant Physiol 2016 Jun;171(2):1511–1522.
36. Oughtred R, Rust J, Chang C, Breitkreutz BJ, Stark C, Willems A, et al. The BioGRID database: A comprehensive biomedical resource of curated protein, genetic, and chemical interactions. Protein Sci 2021 Jan;30(1):187–200.
37. Brandão MM, Dantas LL, Silva-Filho MC. AtPIN: Arabidopsis thaliana protein interaction network. BMC Bioinformatics 2009 Dec;10:454.
38. Calderone A, Castagnoli L, Cesareni G. mentha: a resource for browsing integrated protein-interaction networks. Nat Methods 2013 Aug;10(8):690–691.
39. Kawakatsu T, Huang SSC, Jupe F, Sasaki E, Schmitz RJ, Urich MA, et al. Epigenomic Diversity in a Global Collection of Arabidopsis thaliana Accessions. Cell 2016 Jul;166(2):492–505.
40. Jin J, He K, Tang X, Li Z, Lv L, Zhao Y, et al. An Arabidopsis Transcriptional Regulatory Map Reveals Distinct Functional and Evolutionary Features of Novel Transcription Factors. Mol Biol Evol 2015 Jul;32(7):1767–1773.
41. Tian F, Yang DC, Meng YQ, Jin J, Gao G. PlantRegMap: charting functional regulatory maps in plants. Nucleic Acids Res 2020 Jan;48(D1):D1104–D1113.
42. Mueller LA, Zhang P, Rhee SY. AraCyc: a biochemical pathway database for Arabidopsis. Plant Physiol 2003 Jun;132(2):453–460.
43. Kainer D, Lane M, Sullivan K, Miller J, Cashman M, Morgan M, et al., RWRtoolkit Narrative. Oak Ridge National Laboratory (ORNL), Oak Ridge, TN (United States); 2024. https://www.osti.gov/servlets/purl/2519695/.
44. Kainer D, Lane M, Sullivan KA, Miller JI, Cashman M, Morgan M, et al., RWRtoolkit: a set of command-line and R tools for performing Random-walk with Restart analyses on multiplex networks in any species (Version 1). [Computer software]. Software Heritage; 2025. https://archive.softwareheritage.org/swh:1:snp:d6a688cb39bba99c6401346872649d2efea88dfb;origin=https://github.com/dkainer/RWRtoolkit.
45. RWRtoolkit-data (2025). Comprehensive_Network_AT_d0.5_v02.RData.; 2025. https://github.com/dkainer/RWRtoolkit-data.
46. Kainer D, Lane M, Sullivan KA, Miller JI, Cashman M, Morgan M, et al., RWRtoolkit-data: Multiplex network (Rdata) objects for use with the RWRtools KBase app (Version 1); 2025. Computer software. Available from: https://archive.softwareheritage.org/swh:1:snp:0f97744911eaa2098a614537ee7249bdcc513b3f;origin=https://github.com/dkainer/RWRtoolkit-data.

Click here to access/download
Supplementary Material
RWRtoolkit_GigaScience_SI_Revision.pdf

Click here to access/download
Supplementary Material
Response to Reviewers RWRtoolkit.docx

Personal Cover
Click here to access/download;Personal Cover;Resubmission_GigaScience_CoverLetter.docx

PO Box 2008
Oak Ridge, TN 37830
(865) 574-6134

Dr. Scott Edmunds
Editor in Chief
GigaScience Press
Room A 26F Kings Wing Plaza 2
No. 1 On Kwan Street
Shek Mun, Shatin, N.T., Hong Kong

January 10, 2025

Dear Dr. Edmunds,

Thank you for considering our manuscript for publication in *GigaScience* entitled “RWRtoolkit: multi-omic network analysis using random walks on multiplex networks in any species”.

We have thoroughly updated the manuscript in response to the reviewers’ insightful comments. In particular, we have focused the scope of our manuscript on the improvements and innovations of the RWRtoolkit package compared to the RandomWalkRestartMH package. We present new results demonstrating the improved time efficiency of building multiplex networks using RWRtoolkit, as we have parallelized the generation of the supra-adjacency matrix in order to build multiplex networks with larger numbers of layers and nodes that would be time-prohibitive when using RandomWalkRestartMH. Furthermore, we demonstrate an updated function for weighting the probability of restarting a random walk within a given multiplex layer (tau), which was incorrectly implemented in the RandomWalkRestartMH package, and demonstrate the strong effects that tau weights have on results of random walk exploration using RWRtoolkit. We then include biological use cases in the Supplementary Information in order to illustrate the utility of RWRtoolkit, while keeping the emphasis of the manuscript narrative on the improvements made by this package.

We hope the revised work will be of interest to the readership of *GigaScience*. With the improvements made by the helpful comments of the reviewers, we hope that the research community will benefit from RWRtoolkit as an open-source software package to enhance biological discovery by building increasingly complex multiplex networks at larger scales using multiple lines of biological evidence.

Thank you for your time and consideration,

Daniel A. Jacobson, Ph.D.
Distinguished Research Scientist
Biosciences
Oak Ridge National Laboratory
jacobsonda@ornl.gov
