# Supplementary material for: RWRtoolkit: multi-omic network analysis using random walks on multiplex networks in any species
Source: Gigascience. 2025 Apr 24;14:giaf028. doi: 10.1093/gigascience/giaf028 (PMC12020474; doi:10.1093/gigascience/giaf028)
Supplement: giaf028_Response_to_Reviewer_Comments_Original_Submission [file giaf028_response_to_reviewer_comments_original_submission.pdf]

Author's Response To Reviewer Comments

We appreciate the thoughtful comments provided by both reviewers. We have implemented their feedback which has considerably strengthened the present manuscript. Below are point-by-point responses to each reviewer comment:

Reviewer #1:

Kainer and Lane et al. present a software package, RWRToolkit, for generating and analyzing integrated biological networks based on the Random Walk with Restart (RWR) algorithm. The key deliverable of this work is R software that executes and expands upon an existing RWR package. More specifically, the introduced functionality focuses on identifying genes within the integrated network that are connected to user-inputted seed genes, nominating these candidates as functionally related. But the bulk of the manuscript is actually presenting the results of two genetic analyses in plants, which are also used as examples of using RWR.

Response: We greatly thank the reviewer for pointing this out. We acknowledge that the manuscript originally placed significant emphasis on the case studies. This was done to illustrate the practical applications and robustness of the software across diverse biological scenarios. To address this, we have moved the case studies to the supplemental and have instead focused primarily on descriptions of the software's unique functionalities and emphasized its user-friend design and integration with KBase. We believe this should make the purpose of the paper clearer and simplify the message overall.

The authors have confused their message by presenting novel data and results in a manuscript that is ostensibly about software. Furthermore, the software itself is, by the authors' own description, a "wrap(per)" for RandomWalkRestartMH - the algorithm has been published and is described and evaluated in other papers (that are cited). There is additional functionality, but by focusing so much on evaluating the method and on novel results, the manuscript opens up the door to questions about those specific analyses and data, which I did not find compelling, as well as the RWR method itself, which is also not compelling as presented. This manuscript ultimately reads more like it is an application of RWR inference on the authors' biological problem of interest, rather than an objective assessment of the functionality of the software, much less of RWR itself.

Response: The reviewer's point is well made and we have worked to improve the narrative of the manuscript accordingly. We have revised our manuscript to better distinguish between our case studies and the software itself. We have illustrated the updates made to the RandomWalkRestartMH package codebase (speed improvements that allow for much larger networks, fixed the tau parameter etc), our extended feature implementation, and have left the interpretation of the biological narratives as case studies for readers to browse at their own leisure. The changes can be found in the Data Description, Discussion, and Methods section.

On the positive side, the software offers what seems to be convenient functionality to build integrated networks and facilitate analyses. The Github is well organized and documented, with an extensive test suite. The authors further make their implementation as well as an Arabidopsis integrated network available on a public data repository. The introduction is well-written and efficiently contextualizes the generation of integrated biological networks.

Response: We appreciate the Reviewer's positive opinion of the software's added capabilities in multiplex network building, our GitHub documentation and Arabidopsis multiplex networks, and the Introduction to the manuscript.

However, the manuscript does not provide a sufficient sense for what functionality is missing or underdeveloped in the RandomWalkRestartMH package. That is, what exact problem is solved by RWRToolkit - if someone wanted to do cross-validation using RandomWalkRestartMH for example, what would be involved without RWRToolkit? Based on the vignettes for the RandomWalkRestartMH package, it seems designed for the kind of analyses that the authors here do on the plant genes, is RWRToolkit really necessary to do that? Perhaps RWRToolkit makes it easier? The authors should make the advantages of using RWRToolkit vs. the underlying wrapped package much clearer. Perhaps this can all be addressed by the authors, but the other major problem I have with the manuscript is with the evaluation and applications.

Response: We thank the reviewers for noting that we did not make clear the advantages of using the RWRtoolkit functionality compared to RandomWalkRestartMH. We have updated the manuscript to illustrate why users would wish to use the RWRtoolkit compared to RandomWalkRestartMH, focusing on the software's usability with its file based input and command line interface options as well as KBase integration. Additionally, our updated RandomWalkRestartMH codebase drastically reduces the compute time required for generation, and we have fixed the Tau parameter to properly weigh the seeds across layers, and we have been sure to note these within the "RandomWalkRestartMH Updates" subsections within the Data Description, Results, Discussion, and Methods section.

The evaluation of the RWR algorithm presented in figure 3 is underdeveloped and unconvincing. No context/comparison is provided for how the analyzed multiplex network performs relative to other approaches, other than a shuffled null. One reason I find this unconvincing is that the groups of genes to be tested were selected by the authors because they were, according to the methods section (Network Validation), already known to be highly connected. I have to assume that "highly connected" refers to information that is known to be captured in the networks used. So it seems they have chosen examples that are known, ahead of time, to be likely to "work", but I also expect they will "work" with just about any network analysis method.

Response: We apologize for our lack of clarity here. It was, indeed, incorrect of us to describe the selected groups of genes as "highly connected" as this would imply that we selected genes that were already known to be connected in the multiplex. In reality, the gene sets were selected solely based on shared MAPMAN annotations, meaning the genes in a set are known to have similar functionality/role according to curated evidence, but the multiplex itself was never used for selection. We also recognise that if a network containing connections derived from MAPMAN were included in the multiplex, using gene sets from that database for validation would create a circular argument. We have updated the text to explain the gene set selection more carefully.

If they want to turn this into an algorithm evaluation paper (which I rather advise against), the authors must contrast other algorithms and test the performance of the Arabidopsis multiplex network to a simple "monoplex" network generated from the same input layers, and reconsider what gene sets they use for testing.

Response: We agree with the reviewer's advice here. We do not intend this paper to be an algorithm evaluation.

Additional comparisons of the performance of the multiplex layer relative to each individual layer would also be valuable, given that the entire work is predicated on the superior ability of multi-modal, multiplex networks to predict functionally related genes. But I have to emphasize that embarking on an evaluation of RWR should have been out of scope for a simple "wrapper": the authors are the ones who have opened the door to questioning the efficacy of the method by presenting this evaluation. The only reason I can think they felt the need to include an evaluation was because they then go on to apply RWR to the same network for discovery - that is, perhaps their intention is to evaluate the network, not the algorithm. But since the

evaluation is unconvincing, it doesn't do that too well either.
Response: Thank you for the reviewer's thoughtful suggestion. We agree that while additional comparisons of multiplex network performance relative to individual layers could be valuable, the primary focus of this manuscript is on extending and updating the RandomWalkRestartMH package. As such, conducting comprehensive evaluations of all layers to validate the superiority of multiplex networks is beyond the scope of this work.

The application to the genetics studies doesn't add all that much beyond "the software can be run." Despite this, discussion of the genetic studies takes up a lot of this manuscript - even without the RWR results. This means that I have to evaluate these genetics studies, and to the extent I have done so, they are unimpressive and I am not sure they would stand alone. This starts with the weak results from the GWAS, with four methods applied using a very lenient FDR of 0.2, two of which reporting no convincing associations. The remaining two methods identify 22 SNPs, with only one SNP being shared. I have no sense that this isn't just a false discovery. Despite this, the authors implicate a gene and use its ortholog a seed for the RWR_LOE method to find connected genes in the multiplex Arabidopsis network. All of this can be criticized and questioned - and perhaps choosing a random gene would have been just as interesting as what happens next. The authors use the candidate connected genes to suggest a functional connection to shoot biomass development. This line of speculation might be acceptable in the context of a study generating gene candidates involved in shoot biomass, but it is not a sufficient demonstration of the method's ability to retrieve validated functionally related genes - as the finding themselves are not validated. Further it is not clear that the same candidates would not have been readily identified by examining the top ranked partners of individual networks (i.e., coexpression) or a simplistic/monoplex network, or even just doing GO enrichment. The same sentiment applies to the other analysis of the knockouts, both as an evaluation of the RWR method, and in terms of being a meaningful discovery, or even that compelling of an exploratory analysis. But again, this really seems beside the point for describing the software.'

Response: We acknowledge the reviewer's concern about the extent and emphasis placed on the genetic studies. The primary purpose of these studies was to demonstrate the practical utility and application of the software rather than present standalone biological discoveries. We have revised the manuscript to focus on illustrating RWRtoolkit's functionalities and software updates to RandomWalkRestartMH and shifted the case studies to supplementary material.

Minor comments:

* Multiple figures suffer from poor legibility, such as the legends and axis labels in Fig. 3 and the text of Figs. 4 and 5.
Response: We have updated our figures to have a greater font size with respect to the legends and axes. Particularly, we have additionally updated our illustrations to primarily be pdfs, to ensure the vector graphics of the fonts translate to the document.

* The section "Multiplex Generation" would benefit from a concise overview of how the Multiplex network is generated in the first place in the context of RandomWalkRestartMH, even if this is just a reference to Fig.1. For example, the methods state that "the multiplex constructed with a delta value of 0.5" with no context provided for what this parameter means.
Response: We thank the reviewer for catching our assumptions. We have since included a description of the parameter within the construction section. We have included a concise description of how the multiplex networks were constructed by the RandomWalkRestartMH and how they are now constructed with respect to our updated codebase within the data description RandomWalkRestartMH Multiplex Generation section.

* Fig. 5A seems unnecessary, or at least underdeveloped. There is no need to add text of the status of each set within the Venn diagram; consider instead basic information such as the count of genes in each set. It's also very difficult to read and make sense of the hairballs.
Response: We thank the reviewer for commenting on the illustration. As we thoroughly discuss the processing of the data in the text, we have instead opted to illustrate the major findings with respect to the GO enrichment of the FAE1/FAD2 specific top ranked genes.

* Part of the results (including the main reference to Fig. 5) are in the Discussion, rather than the Analyses section.
Response: We deeply thank the reviewer for catching this mistake. We have since ensured that our results are properly within the results sections of our manuscript.

* The authors claim to "show" that the software is "easy-to-use" (end of the Discussion). I do not see where they have evaluated this, which would require comparing user experience to some alternative, and I don't expect them to do that - I mean, I agree it doesn't seem particularly difficult to use, but it would be more appropriate to say it was designed with ease-of-use in mind. If they mean "easier than the underlying wrapped package", this needs to be described better as referred to under my major points.

Response: We thank the reviewer for nothing that we have not “shown” that the software is “easy-to-use”. We have made it a point to note in our manuscript that we have instead designed the software with “ease of use” in mind.

Reviewer #2:

The paper introduces a species agnostic random walk with restart toolkit built for R and command line users. The tool enables constructions of multiplex networks from any set of data layers and enables the discovery of gene-to-gene relationships. The tool offers a collection of functions for network analysis. Overall, the tool is a significant contribution to network analysis.
Response: We thank the reviewer for their positive feedback and for acknowledging the significance of RWRtoolkit as a contribution to network analysis. We are pleased to hear that you find the toolkit’s features and its potential for multiplex network analysis valuable.

Major Comments

The manuscript's background section should provide a more comprehensive overview of the rationale behind the development of RWRtoolkit. It should clearly outline the existing RWR implementation tools, identify the gaps in these tools, and explain how RWRtoolkit addresses these limitations or offers a new approach.

Response: We very much appreciate this suggestion to expand the background section to better contextualize the development of RWRtoolkit. As the RWRtoolkit fixes and extends the RandomWalkRestartMH package, we have primarily focused on comparisons with the RandomWalkRestartMH package itself.

To demonstrate the effectiveness of RWRtoolkit, the authors could evaluate the ranking performance against other established random walk with restart algorithms that can handle heterogeneous multiplex networks. Additionally, a detailed explanation of the scoring approach implemented in RWRtoolkit is necessary to justify its choice and potential advantages.
Response: Thank you for your thoughtful suggestion. We would like to clarify that the primary goal of this manuscript is to update and extend the original RandomWalkRestartMH package, focusing on enhancing its functionality and usability. While comprehensive algorithm evaluation is beyond the scope of this work, we have included an evaluation of our updated methods with respect to the RandomWalkRestartMH methods. Additionally, we have included a detailed description of the evaluation and validation metric calculations within the supplemental data section. This ensures that users have the necessary context to assess the performance of the software in relevant biological scenarios. We hope this approach strikes an appropriate balance between extending the original package and providing meaningful validation insights.

The authors have indicated in the section "network layer and multiplex statistics" that the tau parameter affects the probability of the walker visiting each specific layer. To address potential bias issues in the network exploration, it would be beneficial to provide an exploration of the parameter space and indicate how it informs the stability of the RWR output scores under variations of the various algorithm parameters.
Response: We agree that an exploration of the tau parameter space is essential for understanding its impact on network exploration. In the revised manuscript, we have included an in-depth analysis of the parameter space with respect to our updated version as well as the prior implementation of RandomWalkRestartMH, focusing on the tau parameter’s effect on the walker’s behavior across layers. This includes visualizations and metrics to illustrate how variations in tau influence RWR output scores for the updated and previous implementations.
