# Supplementary material for: RWRtoolkit: multi-omic network analysis using random walks on multiplex networks in any species
Source: Gigascience. 2025 Apr 24;14:giaf028. doi: 10.1093/gigascience/giaf028 (PMC12020474; doi:10.1093/gigascience/giaf028)
Supplement: giaf028_Reviewer_1_Report_Original_Submission — Paul Pavlidis, Ph.D. -- 9/20/2024 [file giaf028_reviewer_1_report_original_submission.pdf]

Reviewer Report

Title: RWRtoolkit: multi-omic network analysis using random walks on multiplex networks in any species

Version: Original Submission **Date:** 9/20/2024

Reviewer name: Paul Pavlidis, Ph.D.

Reviewer Comments to Author:

Kainer and Lane et al. present a software package, RWRToolkit, for generating and analyzing integrated biological networks based on the Random Walk with Restart (RWR) algorithm. The key deliverable of this work is R software that executes and expands upon an existing RWR package. More specifically, the introduced functionality focuses on identifying genes within the integrated network that are connected to user-inputted seed genes, nominating these candidates as functionally related. But the bulk of the manuscript is actually presenting the results of two genetic analyses in plants, which are also used as examples of using RWR.

The authors have confused their message by presenting novel data and results in a manuscript that is ostensibly about software. Furthermore, the software itself is, by the authors' own description, a "wrap(per)" for RandomWalkRestartMH - the algorithm has been published and is described and evaluated in other papers (that are cited). There is additional functionality, but by focusing so much on evaluating the method and on novel results, the manuscript opens up the door to questions about those specific analyses and data, which I did not find compelling, as well as the RWR method itself, which is also not compelling as presented. This manuscript ultimately reads more like it is an application of RWR inference on the authors' biological problem of interest, rather than an objective assessment of the functionality of the software, much less of RWR itself.

On the positive side, the software offers what seems to be convenient functionality to build integrated networks and facilitate analyses. The Github is well organized and documented, with an extensive test suite. The authors further make their implementation as well as an Arabidopsis integrated network available on a public data repository. The introduction is well-written and efficiently contextualizes the generation of integrated biological networks.

However, the manuscript does not provide a sufficient sense for what functionality is missing or underdeveloped in the RandomWalkRestartMH package. That is, what exact problem is solved by RWRToolkit - if someone wanted to do cross-validation using RandomWalkRestartMH for example, what would be involved without RWRToolkit? Based on the vignettes for the RandomWalkRestartMH package, it seems designed for the kind of analyses that the authors here do on the plant genes, is RWRToolkit really necessary to do that? Perhaps RWRToolkit makes it easier? The authors should make the advantages of using RWRToolkit vs. the underlying wrapped package much clearer. Perhaps this can all be addressed by the authors, but the other major problem I have with the manuscript is with the evaluation and applications.

The evaluation of the RWR algorithm presented in figure 3 is underdeveloped and unconvincing. No context/comparison is provided for how the analyzed multiplex network performs relative to other

approaches, other than a shuffled null. One reason I find this unconvincing is that the groups of genes to be tested were selected by the authors because they were, according to the methods section (Network Validation), already known to be highly connected. I have to assume that "highly connected" refers to information that is known to be captured in the networks used. So it seems they have chosen examples that are known, ahead of time, to be likely to "work", but I also expect they will "work" with just about any network analysis method. If they want to turn this into an algorithm evaluation paper (which I rather advise against), the authors must contrast other algorithms and test the performance of the Arabidopsis multiplex network to a simple "monoplex" network generated from the same input layers, and reconsider what gene sets they use for testing. Additional comparisons of the performance of the multiplex layer relative to each individual layer would also be valuable, given that the entire work is predicated on the superior ability of multi-modal, multiplex networks to predict functionally related genes. But I have to emphasize that embarking on an evaluation of RWR should have been out of scope for a simple "wrapper": the authors are the ones who have opened the door to questioning the efficacy of the method by presenting this evaluation. The only reason I can think they felt the need to include an evaluation was because they then go on to apply RWR to the same network for discovery - that is, perhaps their intention is to evaluate the network, not the algorithm. But since the evaluation is unconvincing, it doesn't do that too well either.
The application to the genetics studies doesn't add all that much beyond "the software can be run." Despite this, discussion of the genetic studies takes up a lot of this manuscript - even without the RWR results. This means that I have to evaluate these genetics studies, and to the extent I have done so, they are unimpressive and I am not sure they would stand alone. This starts with the weak results from the GWAS, with four methods applied using a very lenient FDR of 0.2, two of which reporting no convincing associations. The remaining two methods identify 22 SNPs, with only one SNP being shared. I have no sense that this isn't just a false discovery. Despite this, the authors implicate a gene and use its ortholog a seed for the RWR_LOE method to find connected genes in the multiplex Arabidopsis network. All of this can be criticized and questioned - and perhaps choosing a random gene would have been just as interesting as what happens next. The authors use the candidate connected genes to suggest a functional connection to shoot biomass development. This line of speculation might be okay acceptable in the context of a study generating gene candidates involved in shoot biomass, but it is not a sufficient demonstration of the method's ability to retrieve validated functionally related genes - as the finding themselves are not validated. Further it is not clear that the same candidates would not have been readily identified by examining the top ranked partners of individual networks (i.e., coexpression) or a simplistic/monoplex network, or even just doing GO enrichment. The same sentiment applies to the other analysis of the knockouts, both as an evaluation of the RWR method, and in terms of being a meaningful discovery, or even that compelling of an exploratory analysis. But again, this really seems beside the point for describing the software.
Minor comments:
* Multiple figures suffer from poor legibility, such as the legends and axis labels in Fig. 3 and the text of Figs. 4 and 5.
* The section "Multiplex Generation" would benefit from a concise overview of how the Multiplex network is generated in the first place in the context of RandomWalkRestartMH, even if this is just a reference to Fig.1. For example, the methods state that "the multiplex constructed with a delta value of

0.5" with no context provided for what this parameter means.
* Fig. 5A seems unnecessary, or at least underdeveloped. There is no need to add text of the status of each set within the Venn diagram; consider instead basic information such as the count of genes in each set. It's also very difficult to read and make sense of the hairballs.
* Part of the results (including the main reference to Fig. 5) are in the Discussion, rather than the Analyses section.
* The authors claim to "show" that the software is "easy-to-use" (end of the Discussion). I do not see where they have evaluated this, which would require comparing user experience to some alternative, and I don't expect them to do that - I mean, I agree it doesn't seem particularly difficult to use, but it would be more appropriate to say it was designed with ease-of-use in mind. If they mean "easier than the underlying wrapped package", this needs to be described better as referred to under my major points.

Methods

Are the methods appropriate to the aims of the study, are they well described, and are necessary controls included? Choose an item.

Conclusions

Are the conclusions adequately supported by the data shown? Choose an item.

Reporting Standards

Does the manuscript adhere to the journal's guidelines on minimum standards of reporting? Choose an item.

Choose an item.

Statistics

Are you able to assess all statistics in the manuscript, including the appropriateness of statistical tests used? Choose an item.

Quality of Written English

Please indicate the quality of language in the manuscript: Choose an item.

Declaration of Competing Interests

Please complete a declaration of competing interests, considering the following questions:

- Have you in the past five years received reimbursements, fees, funding, or salary from an organisation that may in any way gain or lose financially from the publication of this manuscript, either now or in the future?
- Do you hold any stocks or shares in an organisation that may in any way gain or lose financially from the publication of this manuscript, either now or in the future?
- Do you hold or are you currently applying for any patents relating to the content of the manuscript?
- Have you received reimbursements, fees, funding, or salary from an organization that holds or has applied for patents relating to the content of the manuscript?
- Do you have any other financial competing interests?
- Do you have any non-financial competing interests in relation to this paper?

If you can answer no to all of the above, write 'I declare that I have no competing interests' below. If your reply is yes to any, please give details below.

I declare that I have no competing interests

I agree to the open peer review policy of the journal. I understand that my name will be included on my report to the authors and, if the manuscript is accepted for publication, my named report including any attachments I upload will be posted on the website along with the authors' responses. I agree for my report to be made available under an Open Access Creative Commons CC-BY license (http://creativecommons.org/licenses/by/4.0/). I understand that any comments which I do not wish to be included in my named report can be included as confidential comments to the editors, which will not be published.

Choose an item.

To further support our reviewers, we have joined with Publons, where you can gain additional credit to further highlight your hard work (see: https://publons.com/journal/530/gigascience). On publication of this paper, your review will be automatically added to Publons, you can then choose whether or not to claim your Publons credit. I understand this statement.

Yes Choose an item.
